# PAK2 is an effector of TSC1/2 signaling independent of mTOR and a potential therapeutic target for Tuberous Sclerosis Complex

**DOI:** 10.1038/srep14534

**Published:** 2015-09-28

**Authors:** Maria M. Alves, Gwenny M. Fuhler, Karla C.S. Queiroz, Jetse Scholma, Susan Goorden, Jasper Anink, C. Arnold Spek, Marianne Hoogeveen-Westerveld, Marco J. Bruno, Mark Nellist, Ype Elgersma, Eleonora Aronica, Maikel P. Peppelenbosch

**Affiliations:** 1 Department of Gastroenterology and Hepatology, Erasmus MC, Erasmus University of Rotterdam, PO Box 2040, NL-3000 CA Rotterdam, The Netherlands; 2 Center for Experimental and Molecular Medicine Academic Medical Center, University of Amsterdam, Meibergdreef 9, NL-1105 AZ Amsterdam, The Netherlands; 3 Department of Neuroscience, Erasmus MC, Erasmus University of Rotterdam, PO Box 2040, NL-3000 CA Rotterdam, The Netherlands; 4 Department of Neuropathology, Academic Medical Center and Swammerdam Institute for Life Sciences, Center for Neuroscience University of Amsterdam, Amsterdam, The Netherlands; 5 Department of Clinical Genetics, Erasmus MC, Erasmus University of Rotterdam, PO Box 2040, NL-3000 CA Rotterdam, The Netherlands; 6 SEIN—Stichting Epilepsie Instellingen Nederland, Heemstede, The Netherlands

## Abstract

Tuberous sclerosis complex (TSC) is caused by inactivating mutations in either *TSC1* or *TSC2* and is characterized by uncontrolled mTORC1 activation. Drugs that reduce mTOR activity are only partially successful in the treatment of TSC, suggesting that mTOR-independent pathways play a role in disease development. Here, kinome profiles of wild-type and *Tsc2*^*−/−*^ mouse embryonic fibroblasts (MEFs) were generated, revealing a prominent role for PAK2 in signal transduction downstream of TSC1/2. Further investigation showed that the effect of the TSC1/2 complex on PAK2 is mediated through RHEB, but is independent of mTOR and p21RAC. We also demonstrated that PAK2 over-activation is likely responsible for the migratory and cell cycle abnormalities observed in *Tsc2*^*−*/*−*^ MEFs. Finally, we detected high levels of PAK2 activation in giant cells in the brains of TSC patients. These results show that PAK2 is a direct effector of TSC1-TSC2-RHEB signaling and a new target for rational drug therapy in TSC.

Tuberous sclerosis complex (TSC) is an autosomal dominant genetic disorder characterized by the presence of benign tumours in the brain and in other vital organs such as the kidneys, heart, eyes, lungs, and skin, and is associated with severe neurological and behavioral symptoms. Up to 90% of patients suffer from epilepsy, and almost 50% have intellectual disabilities^1^. TSC is caused by a heterozygous mutation in either *TSC1*^2^,^3^ or *TSC2*^4^,^5^ which encode for hamartin (TSC1) and tuberin (TSC2), respectively^6^. These two proteins interact with each other^7^ to form a functional heterodimeric complex that controls different aspects of cellular metabolism by its GTPase activating function towards the Ras homolog enriched in brain protein (RHEB)^8^. In a normal situation, the TSC1-TSC2 complex inactivates RHEB by stimulating the conversion of RHEB-GTP to RHEB-GDP, which subsequently downregulates the mammallian target of rapamycin (mTOR) signaling pathway^9^. In the presence of a mutation in either *TSC1* or *TSC2*, the TSC1-TSC2 complex is unable to form and RHEB is kept in a permanently active, GTP-bound state, leading to constitutive activation of the mTOR pathway and, as a consequence, to uncontrolled cell growth. The importance of mTOR signaling downstream of the TSC genes is further illustrated by the observation that upregulation of TSC1 following ischaemia induces protective autophagia via inhibition of the mTOR pathway and can protect neurons from ischaemia-induced cell death^10^.

mTOR is an important regulator of cellular metabolism and has multiple downstream targets including p70S6 kinase and the eukaryotic initiation factor 4E (eIF4E)-binding proteins 1 and 2 (4E-BP1 and 4E-BP2)^11^. Various molecules are potent inhibitors of mTOR and are used clinically to combat disease characterised by excessive mTOR activity, including TSC. Rapamycin and its analogues such as sirolimus and everolimus, play a prominent role in this respect. Rapamycin is a macrolide antibiotic that blocks mTOR signaling by forming an inhibitory complex with the cytosolic FK-binding protein-12 (FKBP-12)^12^. The Rapamycin-FKBP12 complex directly binds and inhibits mTOR when present in a protein complex consisting of mTOR, Raptor, LST8, PRAS and DEPTOR, together known as mTORC1, but not when present in the protein complex mTORC2 (consisting of mTOR, Rictor, LST8, mSIN1, DEPTOR and Protor). mTORC1 inhibition by rapamycin is now firmly established as a therapeutic option in various diseases, especially transplantation medicine and various forms of cancer. Indeed, in two prospective studies sirolimus proved to be safe and effective for the treatment of TSC-associated renal angiomyolipoma^13^,^14^. Furthermore, everolimus substantially and significantly reduced the volume of subependymal giant cell astrocytomas in an open-label phase 1/2 study^15^, while in a larger double-blind placebo-controlled phase III study, it was shown to reduce tumour size and seizure frequency in a significant fraction of patients with TSC and associated subependymal giant cell astrocytoma^16^. Unfortunately, some clinical manifestations of TSC, especially lung function^14^ and neurological symptoms, do not or only partially react to prolonged treatment with rapamycin analogues. Although none of the everolimus-treated patients progressed, only 27 (35%) of the 78 patients in the everolimus-treated group of the phase III study (*versus* none of the placebo group) was considered to have a response in terms of a reduction in the total volume of all subependymal giant cell astrocytomas^16^. Thus mTOR inhibition was only partially effective in these patients. Furthermore, TSC associated tumours are highly autophagia dependent, and as rapamycin analogues are well-known to stimulate autophagia, their administration will provide a pro-survival stimulus that will limit their own efficacy, contributing to the partial clinical response seen for human angiomyolipomas and subependymal giant cell astrocytomas to mTORC1 inhibition^17^. Thus, alternative treatments are urgently called for.

The potential of prolonged rapamycin treatment to cause full mTOR inhibition is undisputed, and hence the failure of mTOR inhibition to counteract all manifestations of TSC indicates that mTOR may well not be the sole molecular mediator of the effects of TSC1/2 deficiency on the cellular phenotype^14^. Obviously, knowledge of TSC1-TSC2-regulated pathways acting in parallel with mTOR activation should prove exceedingly useful for designing superior clinical treatments. These considerations prompted us to contrast signal transduction in *Tsc2*^*−/−*^ and wild-type mouse embryonic fibroblasts (MEFs), employing a peptide array approach that provides a comprehensive description of cellular kinase activity by comparing the phosphorylation of 1024 different kinase substrates^18^,^19^. We have previously shown that kinome profiling is a powerful tool to define therapeutically interesting kinases without *a priori* knowledge of the pathways affected in different pathogeneses, since it allows the generation of complete descriptions of cellular kinase activity in a single experiment^20^,^21^. Our results reveal the role of TSC2 in cellular signal transduction and uncover a previously unrecognised action of this protein in the down regulation of PAK2-dependent signaling, which is independent of mTOR. Thus PAK2 constitutes a new downstream target of the TSC1-TSC2 complex and provides a potential novel avenue for treatment of TSC.

## Results

### The influence of genetic deletion of *Tsc2* on the cellular kinome in MEFs

**Figure 1:**
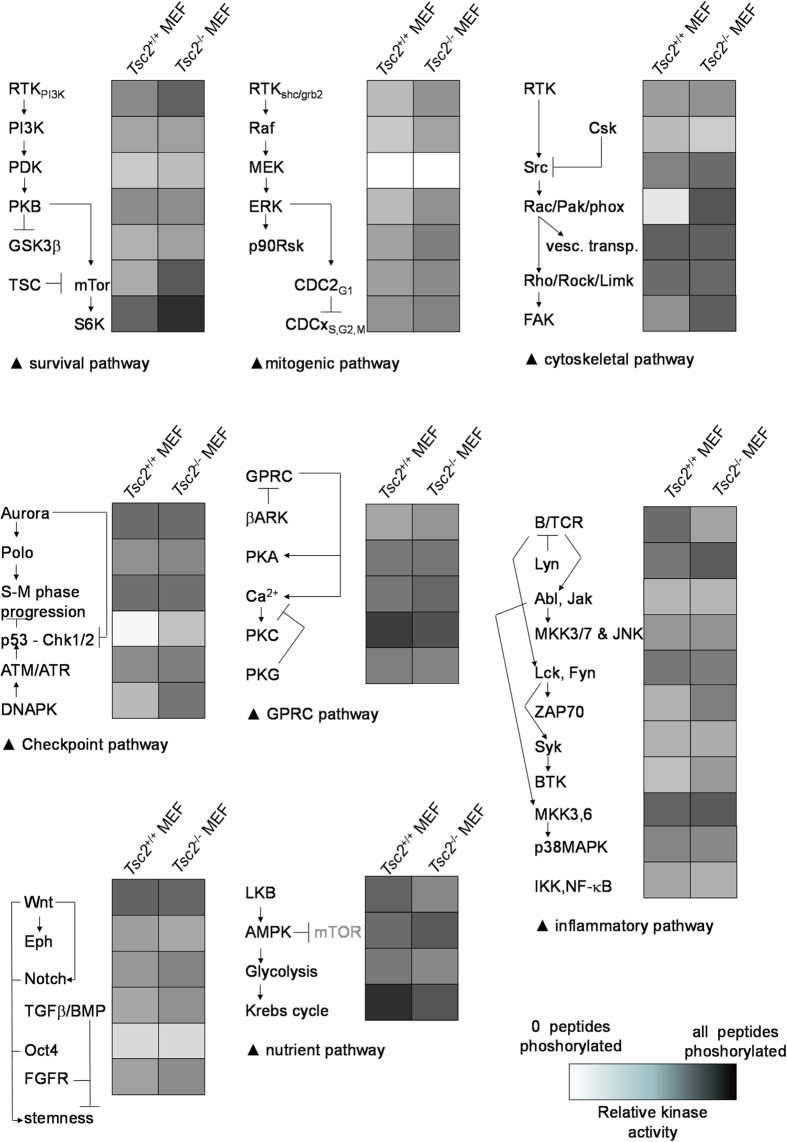
Effect of genetic deletion of *Tsc2* on signal transduction in mouse embryonic fibroblasts (MEFs) as determined by the phosphorylation of peptide arrays containing 1024 kinase substrates. Graphical impression of the results obtained, representing three technical replicas performed on two biological replicates. Individual results for each technical replicate were first subjected to Markov state analysis. (i.e. is the peptide significantly more phosphorylated than the background?) and the number of reactions in which this is the case (an integer between zero [in none of the 6 reactions was significant radioactivity incorporated into the substrate peptide] and six [in all 6 reactions significant radioactivity was incorporated into the substrate peptide]) were determined per peptide for each of the experimental conditions (listed as Markov score, Supplementary Table 1). The Markov scores obtained were collapsed on signal categories that provide insight into the signaling differences between the *Tsc2* proficient and deficient cells and are expressed in grey scale (representing the number of peptides phosphorylated as a fraction of all peptides in that category). Note the difference in mTOR signaling, PAK signaling and Chk1/2-related signaling.

To gain insight into the influence of genetic deletion of *Tsc2* on cellular signal transduction, we compared the kinome of *Tsc2*^*−/−*^ MEFs to that of wild-type MEFs. To this end, cells were serum-starved for two hours before lysates were prepared, as this would be expected to increase signaling of the AMPK signal transduction cassette^22^, and thus accentuate differences in signal transduction between the two different genetic backgrounds. Kinome profiles were then generated by incubating each cell lysate with a peptide array containing different kinase substrates in the presence of ^33^P-γ-ATP. Three technical replicates of two biological replicates of each sample were performed. The arrays incorporated substantial amounts of radioactivity (scans not shown). The technical quality of the profiles was good as the average Pearson product moment was well in excess of 0.85 between both the technical and the biological replicas for all cell types analysed for each condition. From a qualitative comparison, it appeared that the profile of the *Tsc2*^*−/−*^ MEFs was substantially similar to the wild-type control (Supplementary figure S1). However, specific differences were also observed between the profiles of the two conditions (Fig. 1). As expected, the canonical effect of *Tsc2* deletion, an upregulation of mTOR-dependent signaling, is clearly manifest: mTOR substrate peptides as well as substrates for p70S6 kinase, a canonical target for mTOR-dependent signal transduction^12^,^23^,^24^, showed increased phosphorylation when incubated with lysates obtained from *Tsc2*^*−/−*^ MEFs (Fig. 1). Strikingly, AMPK activity, which negatively regulates mTOR through the TSC1-TSC2 complex^25^, is much stronger in the *Tsc2*^*−/−*^ MEFs as well (Fig. 1), possibly reflecting a compensatory response to mTOR activation or a reflection of ATP depletion by over-activation of mTOR dependent signaling by this ADP/AMP-stimulated kinase. There is also a substantial activation of the Chk1/Chk2 cell cycle checkpoint signaling network in the *Tsc2*^*−/−*^ MEFs (Fig. 1), which may represent a p53-dependent endogenous feed-back mechanism activated by loss of *Tsc2* to limit cell cycle progression, and may explain why in experimental animals genetic loss of *Tsc2* enhances tumorigenesis in a p53-deficient context^26^. (Of note, while some *Tsc2*^*−/−*^ mouse models described are p53 deficient^27^, the *Tsc*^*−/−*^ MEFS used in the current study retain p53 expression—see supplementary Figure S2). Furthermore, this observation corresponds well with the fact that the AMPK activator AICAR, which acts upstream of the TSC1-TSC2 complex^11^, activates the p53 pathway in a rapamycin-sensitive fashion^28^. Most interestingly, an up-regulation of the Ras-related C3 botulinum toxin substrate 1/p21 activated kinase 2 (RAC/PAK2) pathway was observed in *Tsc2*^*−/−*^ MEFs. This was evident from increased phosphorylation of substrates directly measuring the activity of this kinase (Fig. 1), but also from increased activity of possible indirect effectors of this kinase, like p125FAK, MKK3/6 and c-Src^29^, in the lysates of *Tsc2*^*−/−*^ MEFs (Supplementary table 2).

Taking into account that PAKs represent a pleiotropic family of kinases implicated in a variety of cell physiological effects, especially in neuronal migration^30^; that TSC is often considered to be a disease of abnormal neuronal migration^31^; and that PAKs are highly druggable^32^, these results suggest the tantalising possibility that PAKs may be involved in the development of TSC. Further investigation was initiated to test this notion.

### Increased activity of Pak2 upon genetic inactivation of the TSC1-TSC2 complex is independent of mTor in MEFs

**Figure 2:**
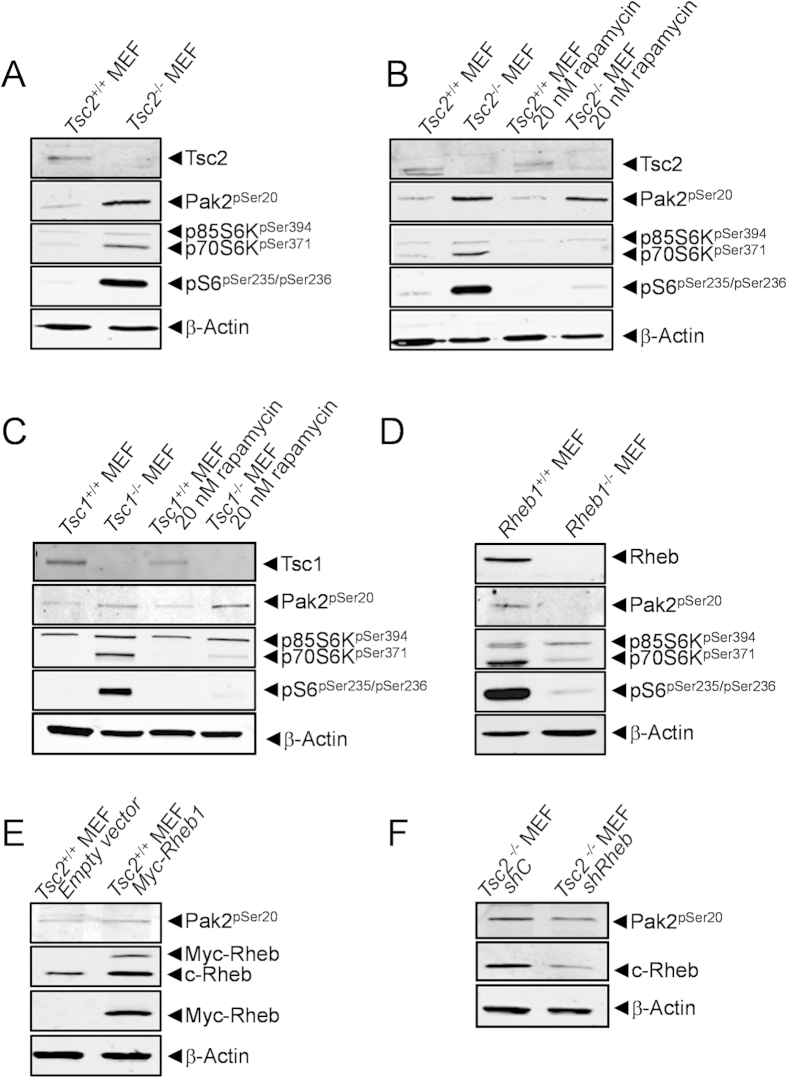
Pak2 phosphorylation is resistant to rapamycin treatment and is dependent of Rheb. (**A)** Western blot analysis of wild-type and *Tsc2*^*−/−*^ MEFs confirms the *Tsc2*-negative status of the *Tsc2*^*−/−*^ MEFS and shows that the increased activity of Pak2 observed in the kinome profile of *Tsc2*^*−/−*^ MEFs corresponds to increase autophosphorylation of Pak2 and increased phosphorylation of p70S6K and S6. (**B**) Treatment of MEFs for 1 hour with 20 nM rapamycin efficiently inhibits increased phosphorylation of p70S6K and S6 following deletion of *Tsc2*, but does not affect the increase in Pak2 autophosphorylation. (**C**) Western blot analysis of wild-type and *Tsc1*^*−/−*^ MEFs shows that loss of *Tsc1* provokes autophosphorylation of Pak2 and phosphorylation of p70S6K and S6. The latter two events are sensitive to a 1 hour treatment of the cells with 20 nM rapamycin, but Pak2 autophosphorylation is unaffected by this treatment. (**D**) Western blot analysis of wild-type and *Rheb1*^*−/−*^ MEFs confirms the Rheb-negative status of the *Rheb1*^*−/−*^ MEFS and shows decreased phosphorylation of Pak2, p70S6K and S6. (**E**) Over-expression of a *Myc*-tagged variant of Rheb in wild-type MEFs induces Pak2 activation. (**F**) Rheb knock down using a short hairpin RNA targeting Rheb, abrogates activation of Pak2 autophosphorylation in *Tsc2*^*−/−*^ MEFs. A control hairpin shows no effect. All blots were run under the same conditions and analysed in a similar way.

The kinome profiles revealed higher Pak2 enzymatic activity in MEFs genetically deficient in *Tsc2.* Western blot analysis of wild-type and *Tsc2*^*−/−*^ MEFS confirmed the Tsc2-negative status of the *Tsc2*^*−/−*^ MEFs, as well as the increased phosphorylation of the canonical mTor targets p70S6K and S6. In addition, the observed increase in Pak2 enzymatic activity in *Tsc2*^*−/−*^ MEFs corresponded to an increased autophosphorylation of Pak2, detected by using an antibody against phospho-serine 20, an autophosphorylation site associated with the activation of this kinase (Fig. 2A). To investigate the mTor-dependency of Pak2 activation in this genetic model, *Tsc2*^*−/−*^ and wild-type MEFs were incubated with rapamycin (Fig. 2B). As expected, rapamycin caused a strong down-regulation of p70S6K and S6 phosphorylation and almost completely abrogated the effects of *Tsc2* deficiency on this phosphorylation. Strikingly, however, Pak2 autophosphorylation measured in parallel was unaffected. Similarly, in *Tsc1*^*−/−*^ MEFs, we observed a clear, rapamycin-sensitive up-regulation of p70S6K and S6 phosphorylation, whereas a strong, rapamycin-insensitive Pak2 auto-phosphorylation was seen in these cells, when compared to wild-type MEFs (Fig. 2C). Thus, our results show that the TSC1-TSC2 complex acts as a negative upstream regulator of Pak2 and that activation of this kinase following *Tsc1* or *Tsc2* deletion occurs in a mTor-independent fashion.

### Rheb regulates Pak2 activation in MEFs

In canonical signal transduction, loss of the TSC1-TSC2 complex influences cellular biochemistry through the subsequent constitutive activation of RHEB and mTOR. The observation that PAK2 activation as a consequence of the genetic loss of one of the components of the TSC1-TSC2 complex is independent of mTOR raises questions as to the role of RHEB in the TSC1-TSC2 dependent control of PAK2 activity. To answer this question we examined Pak2 autophosphorylation levels in *Rheb1*^*−/−*^ MEFs (Fig. 2D). These cells displayed very low levels of autophosphorylated Pak2, demonstrating that constitutive Pak2 activity requires functional Rheb. Furthermore, exogenous expression of a *Myc*-tagged version of Rheb in wild-type MEFs was sufficient to cause increased Pak2 autophosphorylation (Fig. 2E). Finally, when *Tsc2*^*−/−*^ MEFs were transfected with a short hairpin RNA targeting *Rheb1*, the stimulatory effect of genetic deletion of *Tsc2* on Pak2 autophosphorylation was lost. A control short hairpin RNA did not effect either Rheb levels or Pak2 autophosphorylation (Fig. 2F). Taken together, these results indicate that Rheb is a major regulator of the activation status of Pak2.

### Regulation of Pak2 by the TSC1/2-Rheb signaling module is independent of Rac

**Figure 3:**
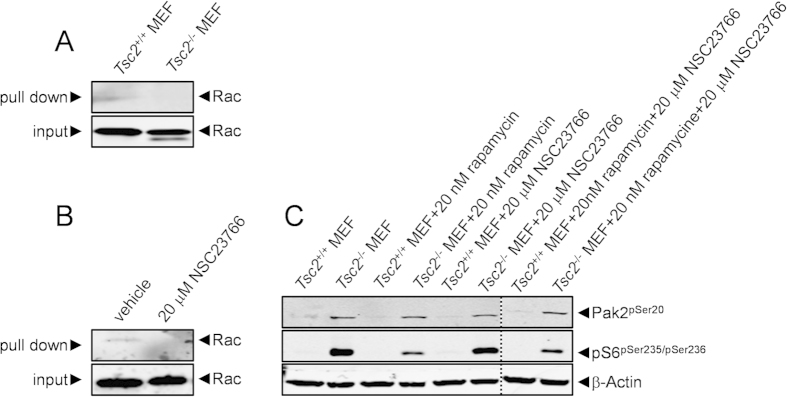
Pak2 autophosphorylation in *Tsc2*^*−*^/^*−*^ MEFs is independent of Rac activity. (**A**) The activation status of Rac was compared in wild-type and *Tsc2*^*−/−*^ MEFs by pull down of Rac-GTP using recombinantly-produced PAK-Crib proteins. *Tsc2*^*−/−*^ MEFs are not characterised by excessive Rac activity. (**B**) Rac-GTP pull down with recombinantly-produced PAK-Crib proteins was used to investigate the effect of a 30 min treatment of 20 μM NSC23766 on Rac activity. The treatment efficiently blocks GTP loading of Rac. (**C**) The effects of a 1 hour treatment with 20 nM rapamycin and a 30 min treatment with 20 μM NSC23766 on Pak2 autophosphorylation and S6 phosphorylation were investigated in wild-type and *Tsc2*^*−/−*^ MEFs. Rapamycin efficiently inhibits S6 phosphorylation but has no effect on Pak2 autophosphorylation. NSC23766 affects neither Pak2 autophosphorylation nor S6 phosphorylation. The combination of rapamycin and NSC23766 (lanes cropped from the same blot) does also not influence Pak2 phosphorylation, nor is it more effective on S6 phosphorylation as compared to rapamycin alone. All blots were run under the same conditions and analysed in a similar way.

As the the canonical regulator of activity of the family of p21-activated kinases is the RAC GTPase^33^, we next investigated the activity of this protein in *Tsc2^−/−^* MEFs. Interestingly, the genetic loss of *Tsc2* in MEFs was not accompanied by increased Rac-GTP loading (Fig. 3A). If anything Rac-GTP levels were lower in *Tsc2*^*−/−*^ MEFs, hinting at the existence of Pak2 negative feedback mechanisms on Rac activation. Thus, we decided to investigate whether loss of *Tsc2* stimulated Pak2 autophosphorylation in the presence of NSC23766, an inhibitor of RAC GDP→GTP exchange^34^. Indeed treatment of wild-type MEFs for 30 min with 20 μM of NSC23766 efficiently impaired GTP loading of Rac (Fig. 3B). Importantly, NSC23766 treatment did not markedly affect the upregulation of Pak2 autophosphorylation in *Tsc2*^*−/−*^ MEFs (Fig. 3C). Furthermore, treating cells with a combination of rapamycin and NSC23766 did not affect Pak2 autophosphorylation (although phosphorylation of S6 was highly sensitive to this treatment). Hence, the TSC1/2-Rheb signaling module controls Pak2 activity in a Rac and mTOR-independent fashion and represent a specific output of this signaling cascade.

### Pak2 affects the migration of *Tsc2* ^*−/−*^ MEFs

**Figure 4:**
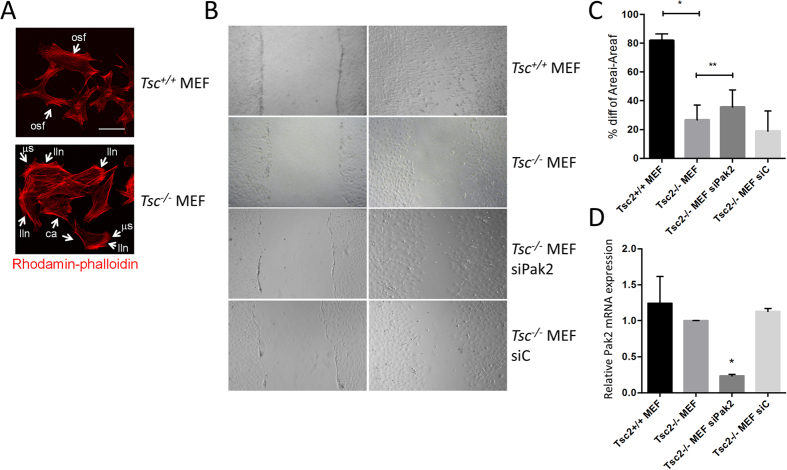
Loss of *Tsc2* results in Pak2 dependent wound healing defects. (**A)** Filamentous actin was visualised by performing rhodamin-phalloidin stainings. Wild-type MEFs are characterised by a relatively modest amount of filamentous actin and highly organised stess fibers (osf; an example is indicated in the figure). Loss of *Tsc2* coincides with robust cytoskeletal rearrangements, including the formation of numerous lamellipodium-like networks (lln), increased and disorganised cytoskeletal actin, the appearance of prominent cortical actin structures (ca) and the formation of many small microspikes (μs). Scale bars 20 μm. (**B**) Scratch wound assays performed in *Tsc2*^*−/−*^ and wild-type MEFs shows that *Tsc2*^*−/−*^ cells migrate slower than wild-type MEFs. Reducing Pak2 by siRNA results in partial restoration of migration in *Tsc2*^*−/−*^ MEFs, whereas control siRNA does not. (**C**) Quantification of the difference in lebesgue measure between the initial (0 hours) and final (24 hours) of the apparent scratch area confirms impaired migration in *Tsc2*^*−/−*^ MEFs (p < 0.001) and restoration thereof by Pak2 siRNA (p = 0.0137). (**D**) Quantitative PCR results demonstrating that levels of Pak2 mRNA expression are not affected in Tsc2^−/−^ MEFs, and are reduced upon siPAK2 treatment, but not control siRNA (*<0.0005).

Predicted consequences of exagerated PAK2 activation are cytoskeletal abnormalities and consequently impaired migratory responses. We visualised filamentous actin by performing rhodamin-phalloidin stainings. Wild-type MEFs were characterised by relatively modest amounts of filamentous actin and highly organised stess fibers (Fig. 4A). In contrast *Tsc2*^*−/−*^ MEFs exhibited robust cytoskeletal reangerangements, including the formation of numerous lamellipodium-like networks, increased disorganisation of cytoskeletal actin, the appearance of prominent cortical actin structures, and the formation of many small microspikes (Fig. 4A). Furthermore, in scratch assays, wild-type MEFs polarised at the wound edge and migrated into the wound space, whereas the *Tsc2*^*−/−*^ MEFS were much less proficient in this respect (Fig. 4B,C). In order to prove that the migratory defect obseved on the *Tsc2*^−/−^ MEFs is induced by Pak2, we reduced *Pak2* expression in these MEFs using a specific siRNA. We also used a non-targeting siRNA as control. We observed that in the *Pak2* siRNA transfected MEFs cell migration was partly restored, while the control siRNA transfected MEFs showed no improvement (Fig. 4B,C). Efficient downmodulation of *Pak2* upon siRNA treatment of *Tsc*^*−/−*^ MEFs was shown by qPCR (Fig. 4D). This experiment furthermore demonstrates that the increased phosphorylation of Pak2 observed (Fig. 2A–C and Fig. 3C) in *Tsc2*^*−/−*^ MEFs as compared to *Tsc2*^*+/+*^ MEFs is independent on Pak2 mRNA expression levels (lanes 1 and 2 in Fig. 4D) and thus relies on posttranslational modification of the Pak2 protein. *In toto*, these experiments show that *Tsc2*^*−/−*^ MEFs demonstrate loss of migratory response which is partially dependent on Pak2.

### Lesions from TSC patients show increased PAK2 autophosphorylation

**Figure 5:**
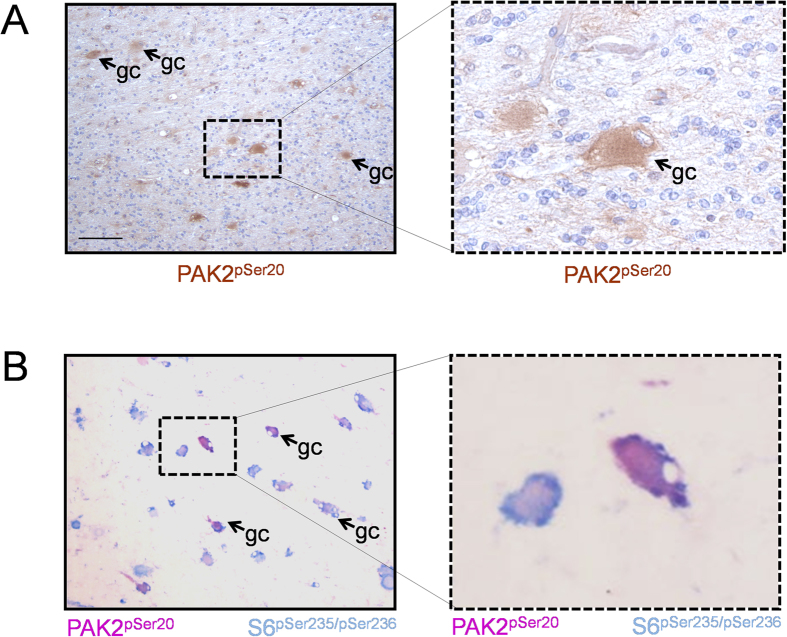
PAK2 phosphorylation levels are increased in brain lesions from TSC patients. (**A**) Immunohistochemistry data showing high levels of PAK2 autophosphorylation in giant cells of TSC patients (representative example of 5 patients). Scale Bars 1 cm; A, B: 80 μM. (**B**) Double-staining in lesions from TSC patients shows co-localization of p-PAK2 and p-S6 in a significant fraction of the giant cells (representative immunohistochemistry analysis of 3 patients; same magnification used as in panel A).

Independent confirmation of PAK2 activation as a downstream effect of loss of the TSC1-TSC2 complex came from experiments in which PAK2 autophosphorylation was investigated in the brains of TSC patients. Pathologically, TSC is characterized by the occurrence of multiple cortical tubers in the cerebral cortex which are thought to be responsible for many neuropsychiatric symptoms of TSC. Histologically, the most conspicuous feature of cortical tubers is the presence of giant cells, which show abnormal size and differentiation markers^35^. Strikingly, strong immunoreactivity for autophosphorylated PAK2 was observed in brain lesions from 5 patients carrying a *TSC1* or *TSC2* mutation (1 *TSC1*: 4 *TSC2*). The staining was restricted to the giant cell compartment and was cytoplasmic, as expected based on the normal localization of this kinase (Fig. 5A). Double-staining for autophosporylated PAK2 and phosphorylated S6 was also performed in three lesions (all patients carrying *TSC2* mutations). The majority of giant cells showed high levels of phosphorylated S6, the golden standard for positive identification of giant cells with loss of heterozygosity for *TSC1* or *TSC2*^36^, and were strongly positive for PAK2 autophosphorylation (Fig. 5B). Thus PAK2 activation accompanies TSC loss in human disease.

## Discusion

**Figure 6:**
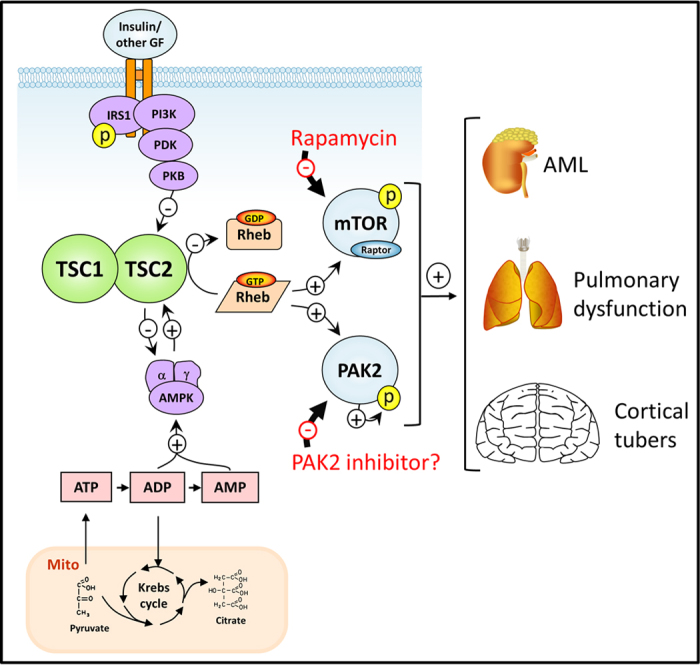
Schematic representation of the TSC1-TSC2-RHEB network showing PAK2 as a new downstream target of RHEB. AML; angiomyolipoma, Mito; mitochondrion, GF; growth factor. Figure was designed by the authors, with no copyright owed elsewhere.

The search for new and better therapeutical targets is a constant pursuit in the treatment of different disorders. This is especially the case for Tuberous Sclerosis Complex (TSC), as TSC symptoms are only partially resolved by current therapy. In particular, the treatment of the neuropsychiatric manifestations is as yet inadequate. So far, most attention in the search for therapeutics has been given to the mTORC1 complex as it constitutes the canonical target of the TSC1-TSC2 complex. Here, we provide evidence that PAK2 signaling constitutes a second signal transduction module downstream of the TSC1-TSC2-RHEB signal transduction complex, whose inhibition may be potentially useful, as strong PAK2 activation appears to be a characteristic of human disease, at least in the brain. A proposed possible diagram as to how PAK2 activation might be implicated in TSC is provided in Fig. 6.

PAK2 is a serine/threonine kinase involved in cytoskeletal reorganization originally identified by its role in chemotactic signal transduction^37^, and is now well-established to be a mediator of directed cell migration in a variety of settings in the body^38^. In the context of TSC, to our knowledge no information has been reported hitherto, but it is well recognised that many of the psychoneurological manifestations of the disease involve aberrant migration of neuronal precursors^39^. In addition, loss of *Tsc1* in mouse subventricular zone neural stem/progenitor cells leads to migration deficits that result in the development of nodular protusions and small tumours that present many features of the brain lesions found in TSC patients^40^. Thus, it is tempting to propose that at least certain aspects of TSC, especially those related to aberrant migration, may involve superactivation of PAK2, rather than that of mTOR. Intriguingly, LKB1, a canonical upstream element in TSC1-TSC2-RHEB-mTOR signaling^41^, is well-established to regulate cell motility and axon growth^42^, and thus it is possible that TSC1-TSC2 complex-regulated PAK2 activation may be involved in the pathophysiological effects of this important tumour suppressor as well. Indeed, a correlation between increased PAK phosphorylation and biallelic *LKB1* inactivation has been reported both in non-small cell lung cancer^43^ and breast cancer^44^. Thus, the observation that PAK2 acts as a mTOR-independent effector of TSC1-TSC2-RHEB signaling is not only highly novel, but it also fits well with the body of available biomedical literature.

Interestingly, the PAKs constitute a family of highly druggable kinases and many inhibitors, including those that target PAK2, have been developed^45^. These compounds have yet to be tested in humans, but the relation between loss of functional TSC1/TSC2 and PAK2 hyperactivation makes it tempting to suggest that TSC could constitute a good candidate disease for such clinical trials. In any case, the current study has shown that the PAK2 kinase constitutes a *bona fide* mTOR-independent effector of the TSC1-TSC2 pathway and thus provides an important starting point for novel thinking on the development of new therapeutic avenues for dealing with TSC.

## Materials and Methods

### Kinome array analysis

The protocol of the kinome array has been provided before in extensive detail^46^. Cell lysates were prepared with m-PER lysis buffer (Thermo-Scientific) containing protease (Roche) and phosphatase inhibitors (Thermo-Scientific). Protein quantification was done using Bradford analysis (Bio-Rad) and 100 μg of protein was used for kinome analysis. After drying, the glass slides were exposed to a phosphor-imager plate for 72 hours and assessed with ScanAlyze software (http://rana.lbl.gov/EisenSoftware.htm). Spot density and individual background intensities were analysed using grid tools and data from two individual experiments were exported to an excel sheet for further analysis. Individual results for each technical replicate were first subjected to Markov state analysis (i.e. is the peptide significantly more phosphorylated than background) and the number of reactions in which this is the case (an integer number between zero [in none of the 6 reactions significant radioactivity in incorporated into the substrate peptide] and six [in all 6 reactions significant radioactivity was incorporated into the substrate peptide]) is listed for each peptide for each of the experimental conditions (listed as Markov score). These Markov scores were collapsed on signal categories that provide insight into the difference in signaling between the *Tsc2* proficient and deficient cells.

### Expression vectors

pcDNA-Myc-Rheb was kindly provided by Liz Henske (Division of Pulmonary and Critical Care Medicine, Department of Medicine, Brigham and Women’s Hospital, Boston, U.S.A). pLKO.1-shRheb was generated in the Biomics Center of the Erasmus Medical Center (Rotterdam, The Netherlands) using the MISSION^tm^ shRNA library (Sigma-Aldrich). The shRheb targeting sequence is CCTCAGACATACTCCATAGAT. pGEX4T-PAK-Crib vector contains the RAC-binding domain of PAK2 and was cloned using BamHI and EcoRI restriction sites.

### Cell culture and transfection

*Tsc1*^*−/−*^, *Tsc2*^*−/−*^ and wild-type MEFs were kindly provided by David Kwiatkowski (Division of Pulmonary and Critical Care Medicine, Department of Medicine, Brigham and Women’s Hospital, Boston, U.S.A). *Rheb1*^*−/−*^ and wild-type MEFs were prepared as described before^47^. All MEFs were cultured in DMEM with high glucose content (Lonza), supplemented with 10% fetal calf serum (Sigma-Aldrich) and 1% penicillin/streptomycin (Gibco). Cells were cultured at 37^o^C and 5% CO_2_. For transient transfection, 300. 000 cells were seeded in 6 well plates. Transfections were performed 24 hours after seeding using Lipofectamine 2000 (Invitrogen). For the siRNA experiments, a SMARTpool: Accell siRNA specifically targeting Pak2 and a SMARTpool: Accell siRNA control were used (GE Healthcare, Dharmacon). Transfection was performed according to the manufacturers’ instructions 24 hours after seeding.

### Treatments, cell lysates, and Western blot analysis

Before lysis, cells were incubated for 2 hours in serum free medium, after which they were treated with rapamycin (1 h, 20 nM) and/or NSC23766 (30 min, 20 μM, Tocris Bioscience). Stimulations were terminated by an ice-cold phosphate buffered saline wash and cells were incubated with lysis buffer (m-PER containing protease and phosphatase inhibitors) for 30 minutes on ice. Cell lysates were collected by scraping and cleared by centrifugation at 14 000 rpm for 10 minutes in a pre-cooled (4 °C) centrifuge. Cell lysates were stored at −80 °C before they were processed further for SDS-PAGE and immunoblotting. The following antibodies were used: TSC2 and actin (Santa Cruz Biotechnology); TSC1, MYC, p-PAK2, p-p70S6K and p-S6 (Cell Signaling); Rheb antibody was kindly provided by Richard Lamb (Department of Oncology, University of Alberta, Cross Cancer Institute, Edmonton, Alberta, Canada). Secondary antibodies used were IRDye 800CW Goat anti-Rabbit and IRDye 680RD Goat anti-Mouse (Li-Cor).

### Pull-down assays

RAC activation^48^ was assessed using pull-down assays. Cells were lysed with 1X GST buffer (10% glycerol, 50 mM Tris-HCl pH7.4, 200 mM NaCl, 1% NP-40, 2 mM MgCl_2_, 1X protease inhibitors and 1X phosphatase inhibitors) and kept on ice. GST-PAK-Crib coupled beads were prepared by incubating GST-PAK-Crib with GST-beads (GE Healthcare) and 1X GST lysis buffer for 45 minutes at 4 °C. Beads were then washed 5 times with 1X GST buffer and incubated with 750 μg of total protein lysate for 45 minutes at 4 °C on a rotating wheel. Samples were washed 3 times with 1X GST buffer and the beads were resuspended in 20 μl Laemmli buffer followed by denaturation at 95 °C for 5 min. Samples were stored at −80 °C before being processed by SDS-PAGE followed by western blot analysis.

### Microscopy and image analysis

For immunofluorescence analysis^49^, cells were cultured on cover slips. Two percent paraformaldehyde (PFA) was used as a fixative and the cells were permeabilised with 1% BSA and 0.1% Triton X-100 in PBS. Phalloidin-rhodamin (Santa Cruz Biotechnology) was added at a concentration of 1:200. Fluorescent images were made using a Leica (AOBS) microscope.

### Migration Assays

Cells were plated in six-well plates and maintained in complete medium. Once the cells reached 80–90% confluence, a scratch was made in the center of the cell monolayer using a sterile plastic pipette tip. Cells were washed immediately with PBS to remove floating cellular debris and incubated for an additional 24 hours. The ability of the cells to migrate into the scratch area was assessed in standard growth conditions after 24 hours by comparing the 0- and 24-hour phase-contrast micrographs of six marked points along the scratched area on each plate. The total remaining scratch area was determined using ImageJ software (Rasband, W.S., ImageJ, U.S. National Institutes of Health, Bethesda, Maryland, USA). Results are presented as the difference percentage of each time point. For the siRNA experiments, the scratch was made 72 hours after transfection, and cells were analysed as described above.

### RNA extraction and RT-PCR

RNA extraction was performed using a RNA extraction kit (Qiagen) according to the manufacturers’ instructions. 500 ng of RNA was used as template for cDNA preparation with the iScript cDNA Synthesis Kit (Bio-Rad). Quantitative PCR was performed in a IQ5 cycler PCR machine (Bio-Rad), using the Kapa SYBR Fast qPCR kit (Kapa Biosystems). Primers used are described in Supplementary table 1. Expression levels were corrected for expression of two house-keeping genes, *Actb* and *Gapdh,* averaged and presented as fold changes.

### Tissue preparation and immunohistochemistry

Immunohistochemistry was performed on tissue samples from five TSC patients (M/F: 3/2; TSC1/TSC2: 1/4). All patients included in this study fulfilled the diagnostic criteria for TSC^50^ and were obtained from the archives of the Departments of Neuropathology of the Academic Medical Center (University of Amsterdam) and the University Medical Center Utrecht (UMCU). Tissue was obtained in accordance with the Declaration of Helsinki and the Academic Medical Center (AMC) Research Code.

Tissue was fixed in 10% buffered formalin and embedded in paraffin. Paraffin-embedded tissue was sectioned at 6 μm, mounted on organosilane-coated slides (Sigma, St. Louis, MO) and used for immunohistochemical staining. Paraffin-embedded sections were deparaffinized, re-hydrated, and incubated for 20 min in 0.3% H_2_O_2_ diluted in methanol to quench endogenous peroxidase activity. Antigen retrieval was performed by incubation for 10 min at 121 °C in citrate buffer (0.01 M, pH 6.0). Sections were washed with PBS and incubated for 30 min in 10% normal goat serum (Harlan Sera-Lab). Incubation with anti p-PAK2 antibody (Cell Signalling, 1:50) was done overnight at 4 °C. Thereafter, sections were washed in PBS and stained with ready-to-use Powervision peroxidase system (Immunologic) and 3,3′-diaminobenzidine (DAB; Sigma) as chromogen. Sections were counterstained with haematoxylin, dehydrated and coverslipped.

For double-labeling, after incubation with p-PAK2 antibody, sections were incubated with Brightvision poly-alkaline phosphatase (AP)-anti-Rabbit (Immunologic) for 30 minutes at room temperature. Sections were washed with PBS and the pH was adjusted with Tris-HCl buffer (0,1 M; pH 8,2). AP activity was visualized with the AP substrate kit I Vector Red (SK-5100, Vector laboratories Inc.). Sections were then incubated at 121 °C in citrate buffer (10 mM NaCi, pH 6.0) for 10 minutes, in order to remove the first primary antibody. Incubation with the second primary antibody (p-S6, Cell Signaling Technology, 1:50) was performed overnight at 4 °C. AP activity was visualized with the AP substrate kit III Vector Blue (SK-5300, Vector laboratories). Controls performed with depletion of the primary antibody showed no staining in any of the material tested (data not shown).

## Additional Information

**How to cite this article**: Alves, M. M. *et al.* PAK2 is an effector of TSC1/2 signaling independent of mTOR and a potential therapeutic target for Tuberous Sclerosis Complex. *Sci. Rep.* **5**, 14534; doi: 10.1038/srep14534 (2015).

## Supplementary Material

Supplementary Information
