# Supplementary material for: PAK2 is an effector of TSC1/2 signaling independent of mTOR and a potential therapeutic target for Tuberous Sclerosis Complex
Source: Sci Rep. 2015 Sep 28;5:14534. doi: 10.1038/srep14534 (PMC4585940; doi:10.1038/srep14534)
Supplement: Supplementary Information [file srep14534-s1.pdf]

Supplementary Information belonging to:

PAK2 is an effector of TSC1/2 signaling independent of mTOR and a potential therapeutic target for Tuberous Sclerosis Complex

Maria M. Alves[1#], Gwenny M. Fuhler[1], Karla C.S. Queiroz[2], Jetse Scholma[1], Susan Goorden[3], Jasper Anink[4], C. Arnold Spek[2], Marianne Hoogeveen-Westerveld[5], Marco J. Bruno[1], Mark Nellist[5], Ype Elgersma[3], Eleonora Aronica[4,6] and Maikel P. Peppelenbosch[1*]

Contains:

Supplementary Figure 1: Correlation between kinome profiles of Tsc 2-/- and wild type MEFs.

Supplementary Figure 2: Verification of *Pak2* downmodulation upon siRNA treatment in *Tsc2*$^{-/-}$ MEFs

Table S1: PCR primers

Supplementary Figures 3A-E: Full blots belonging to Figure 2.

Supplementary Figures 4A-C: Full blots belonging to Figure 3.

Table S2: Kinomic changes in TSC-/- vs wild type MEFs

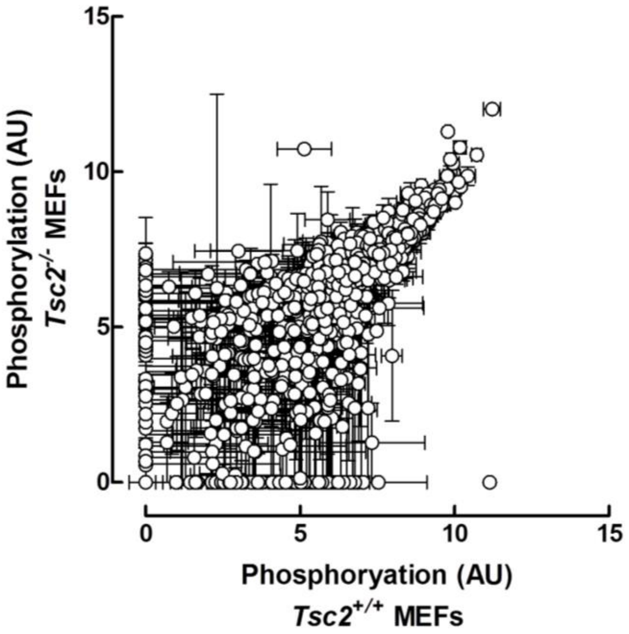

Supplementary Figure S1: Correlation between kinome profiles of Wild-type (Tsc2$^{+/+}$) and Tsc2$^{-/-}$ MEF. Phosphorylation of individual protein substrates are depicted. Mean of two independent experiments and technical triplicates is shown per substrate. Only reliable ‘on’ or ‘off’ spots are used for calculations.

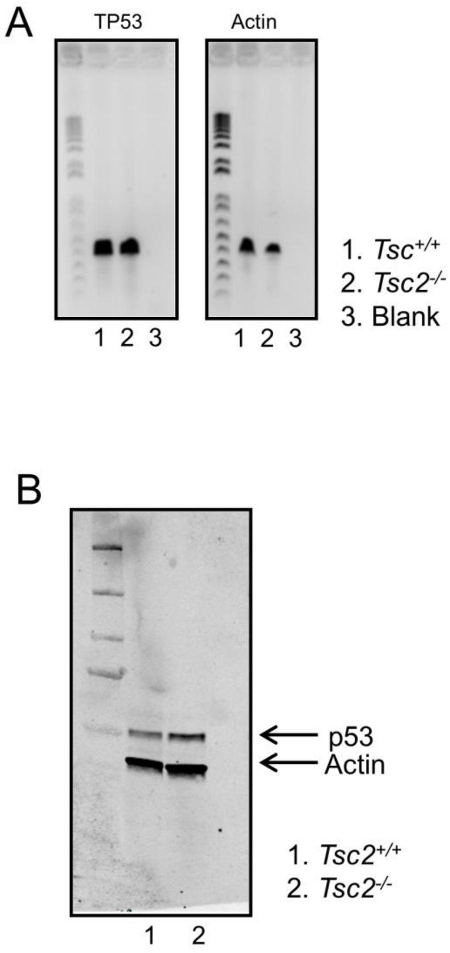

Supplementary Figure S2: Confirmation of p53 expression in *Tsc2$^{-/-}$* MEFs. A) mRNA was isolated from *Tsc2$^{+/+}$* and *Tsc2$^{-/-}$* MEFs, and rt-PCR was performed (see **Supplementary Table 2** for primer details)**,** showing similar TP53 expression in *Tsc2$^{+/+}$* and *Tsc2$^{-/-}$* MEFs (left panel). PCR for actin demonstrated equal mRNA input (right panel). **B)** Proteins were extracted from *Tsc2$^{+/+}$* and *Tsc2$^{-/-}$* MEFs, and p53 was detected by western blot analysis demonstrating the presence of p53 in both cell types. Equal loading was confirmed by probing the same membrane with anti-actin antibodies.

Supplementary table 1: PCR primers

Primer name	Sequence (5'-3')
Pak2qPCRF	ATGATGAAGACGCTGCTCCT
Pak2qPCRR	TCTGAAGACTTGGCACCACT
ActbqPCRF	CCTCTATGCCAACACAGTGC
ActbqPCRR	CACACAGAGTACTTGCGCTC
GapdhqPCRF	CCTTCCGTGTTCCTACCCC
GapdhqPCRR	GTCCTCAGTGTAGCCCAAGA
Tp53F	ACATAGCAAGTTGGAGGCCA
Tp53R	GACAAAAGATGACAGGGGCC
ActbF	ACGGCCAGGTCATCACTATT
ActbR	CACCCTCACCAAGCTAAGGA

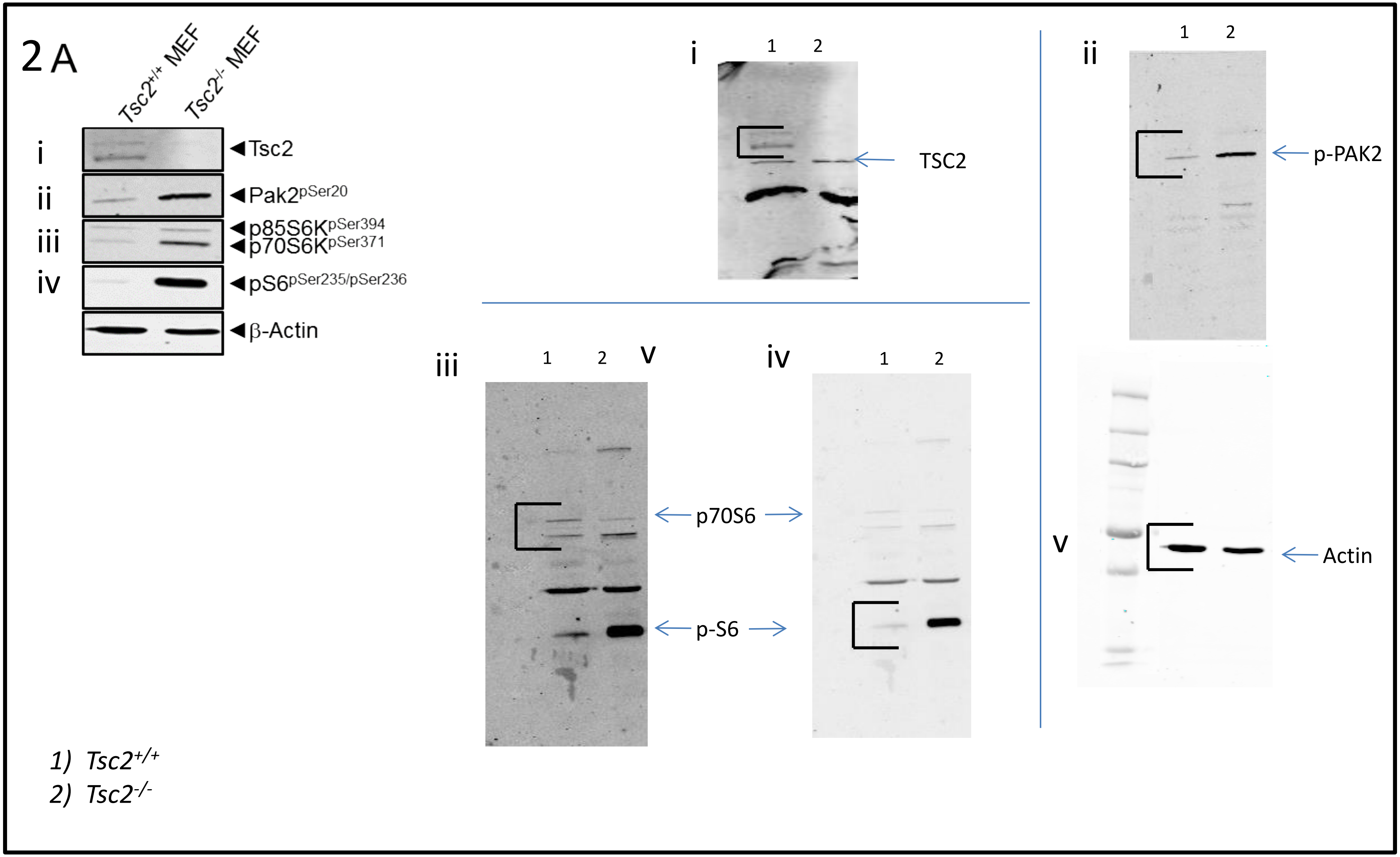

Supplementary Figure S3A: Full length blots belonging to Figure 2A. Top left corner: figure as presented in paper. Full lenght blots of individual panels are shown, croplines are indicated in black. Cell lysates were applied to three individual gels. One gel was used to blot p-P70S6 and p-S6 simultaneously. However the image selected for p-P70S6 resulted from a longer exposure of the same blot. p-PAK2 and TSC2 were blotted on the other two gels. The p-PAK blot was reprobed for actin to confirm equal protein concentration in the lysates. The other blots were also reprobed for actin, with identical results (not shown). All the blots were performed under the same conditions and the data was analysed in the same way for all the antibodies used. Croplines are indicated in the full length blots.

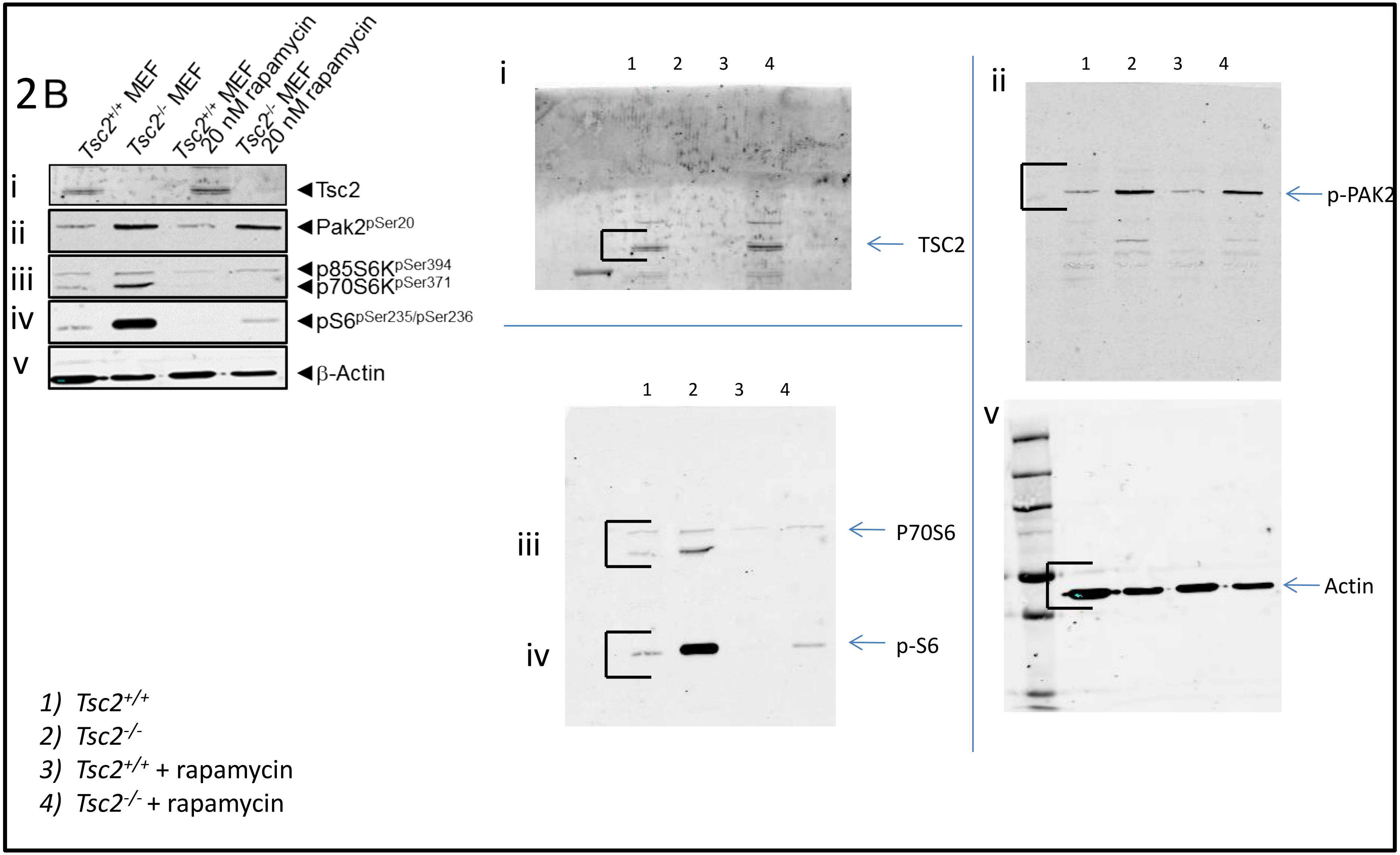

Supplementary Figure S3B: Full lenth blots belonging to Figure 2B. Top left corner: figure as presented in paper. Cell lysates were applied to three individual gels. One gel was used to blot p-P70S6 and p-S6 simultaneously. p-PAK2 and TSC2 were blotted on the other two gels. The p-PAK blot was reprobed for actin to confirm equal protein concentration in the lysates. The other blots were also reprobed for actin, with identical results (not shown). All the blots were performed under the same conditions and the data was analysed in the same way for all the antibodies used. Croplines are indicated in the full length blots.

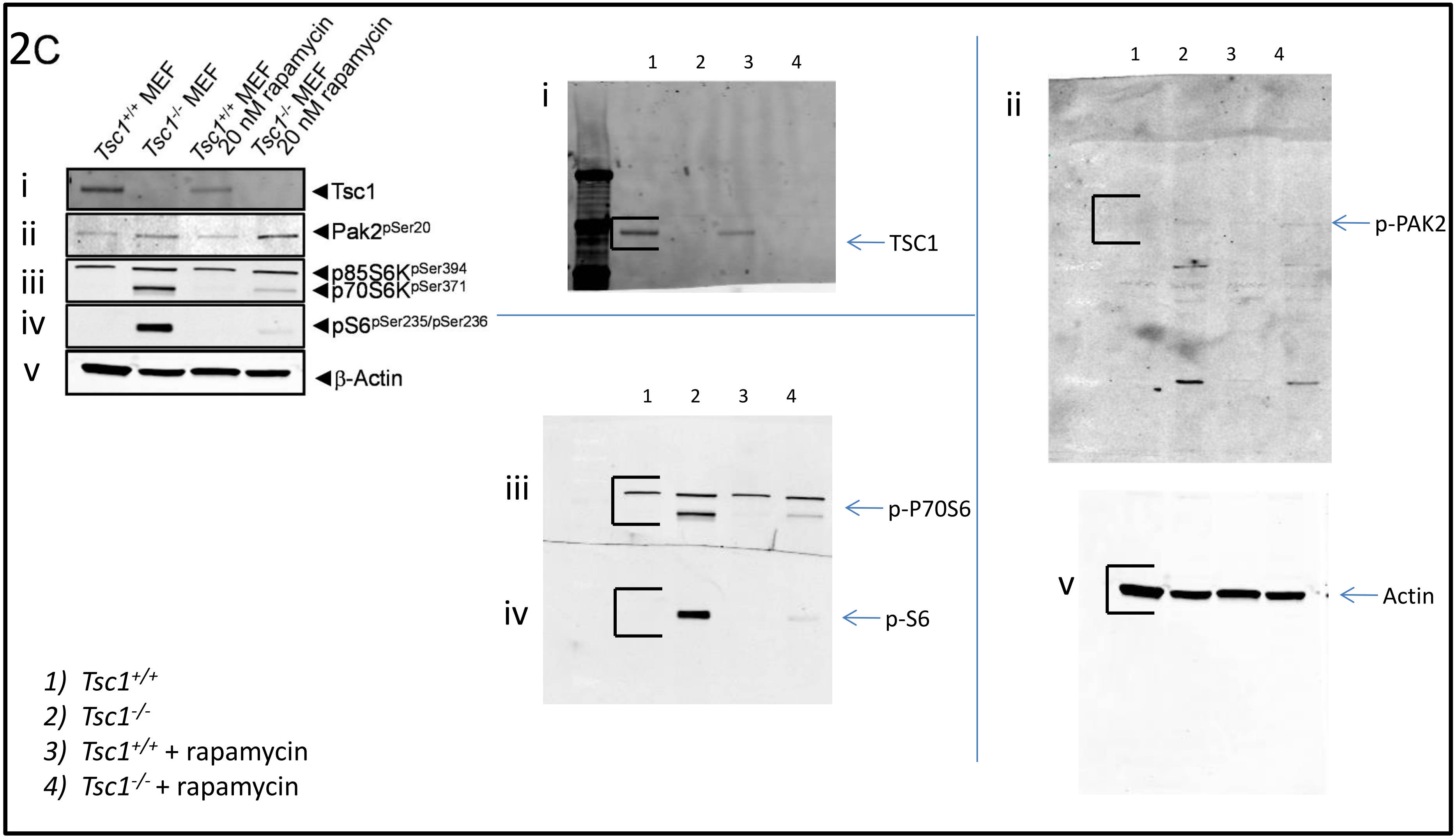

Supplementary Figure S3C: Full lenth blots belonging to Figure 2C. Top left corner: figure as presented in paper. Cell lysates were applied to three individual gels. One gel was used to blot p-P70S6 and p-S6 simultaneously. p-PAK2 and TSC2 were blotted on the other two gels. The p-PAK blot was reprobed for actin to confirm equal protein concentration in the lysates. The other blots were also reprobed for actin, with identical results (not shown). All the blots were performed under the same conditions and the data was analysed in the same way for all the antibodies used. Croplines are indicated in the full length blots.

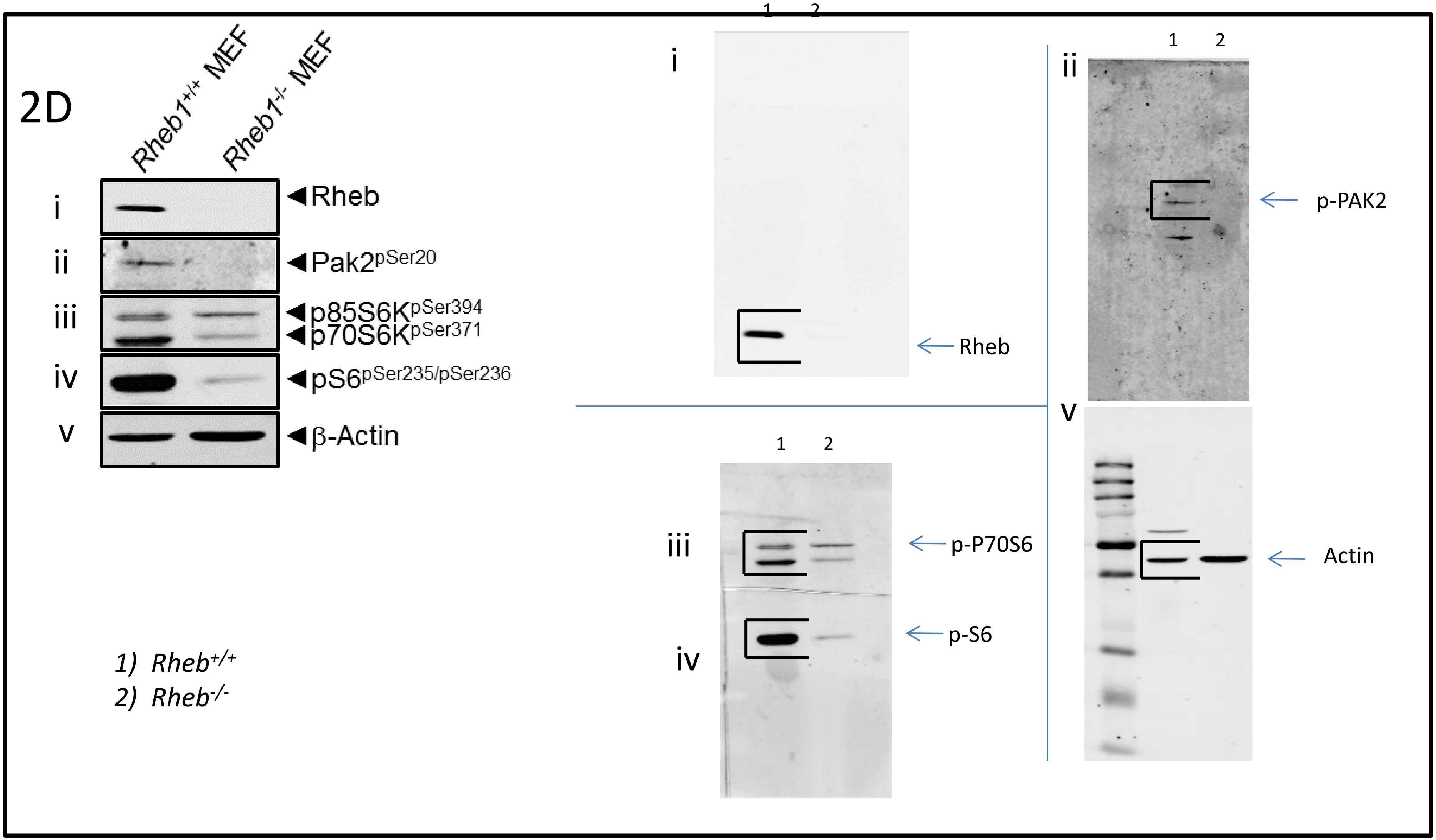

Supplementary Figure S3D: Full lenth blots belonging to Figure 2D. Top left corner: figure as presented in paper. Cell lysates were applied to three individual gels. One gel was used to blot p-P70S6 and p-S6 simultaneously. p-PAK2 and Rheb were blotted on the other two gels. The p-PAK blot was reprobed for actin to confirm equal protein concentration in the lysates. The other blots were also reprobed for actin, with identical results (not shown). All the blots were performed under the same conditions and the data was analysed in the same way for all the antibodies used. Croplines are indicated in the full length blots.

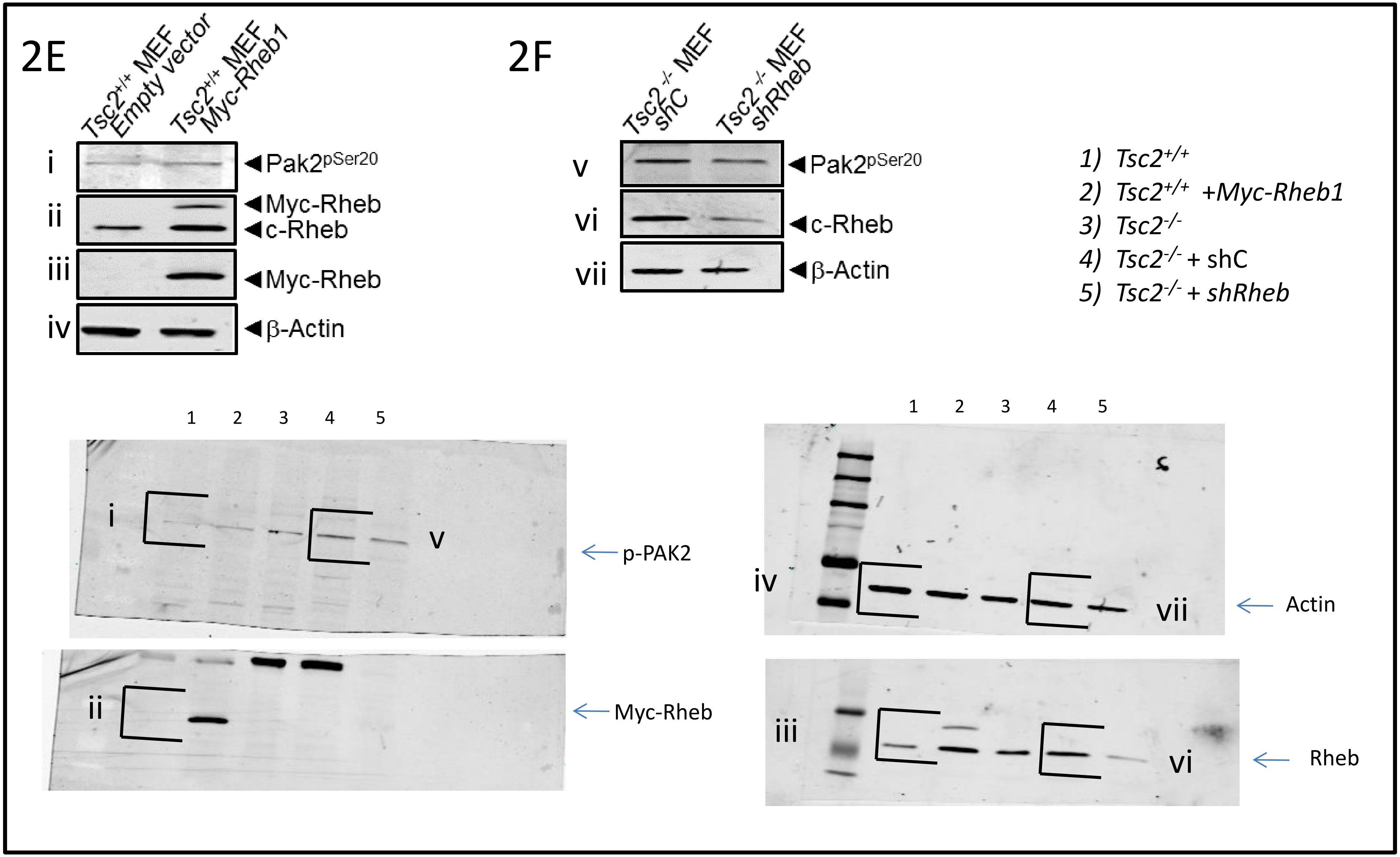

Supplementary Figure S3 E: Full lenth blots belonging to Figures 2 E and F. Top left corner: figure as presented in paper. Cell lysates were applied to one gel. The blot was cutt below the 50kDa band of the marker and the upper part was blotted with p-PAK2, and reprobed for actin, and the lower part was blotted with Myc and reprobed for Rheb. All the blots were performed under the same conditions and the data was analysed in the same way for all the antibodies used. Lane 3 is not used for Figure 2 E and F. Croplines are indicated in the full length blots.

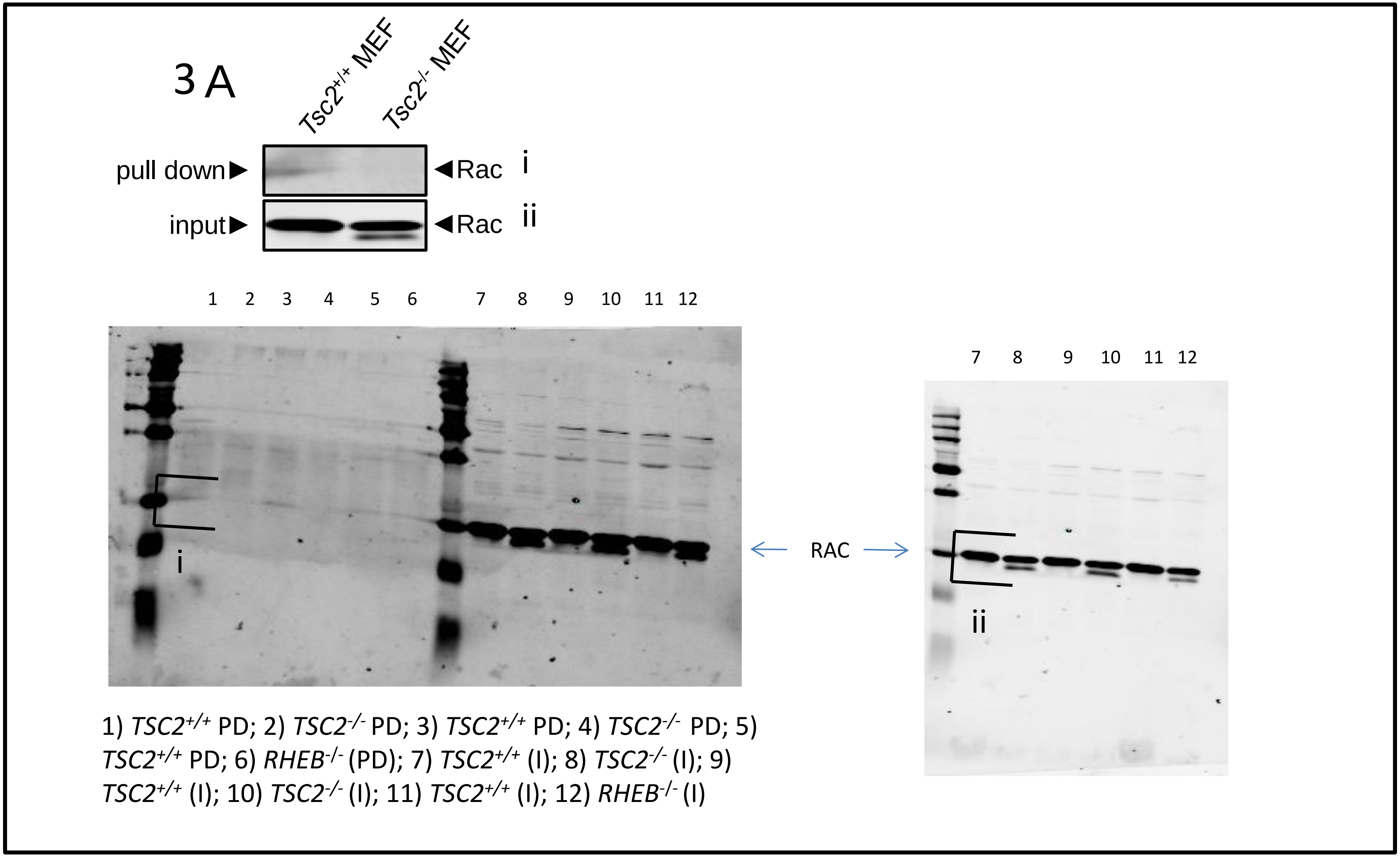

Supplementary Figure S4A: Full lenth blots belonging to Figures 3A. Top left corner: figure as presented in paper. Pull down and input samples were run on the same gel, which was subsequently blotted for Rac. The blot on the right is a lower exposure of the blot on the left. The lower exposure is used in the manuscript. Lanes 1, 2, 7 and 8 were used in the manuscript. Croplines are indicated in the full length blots.

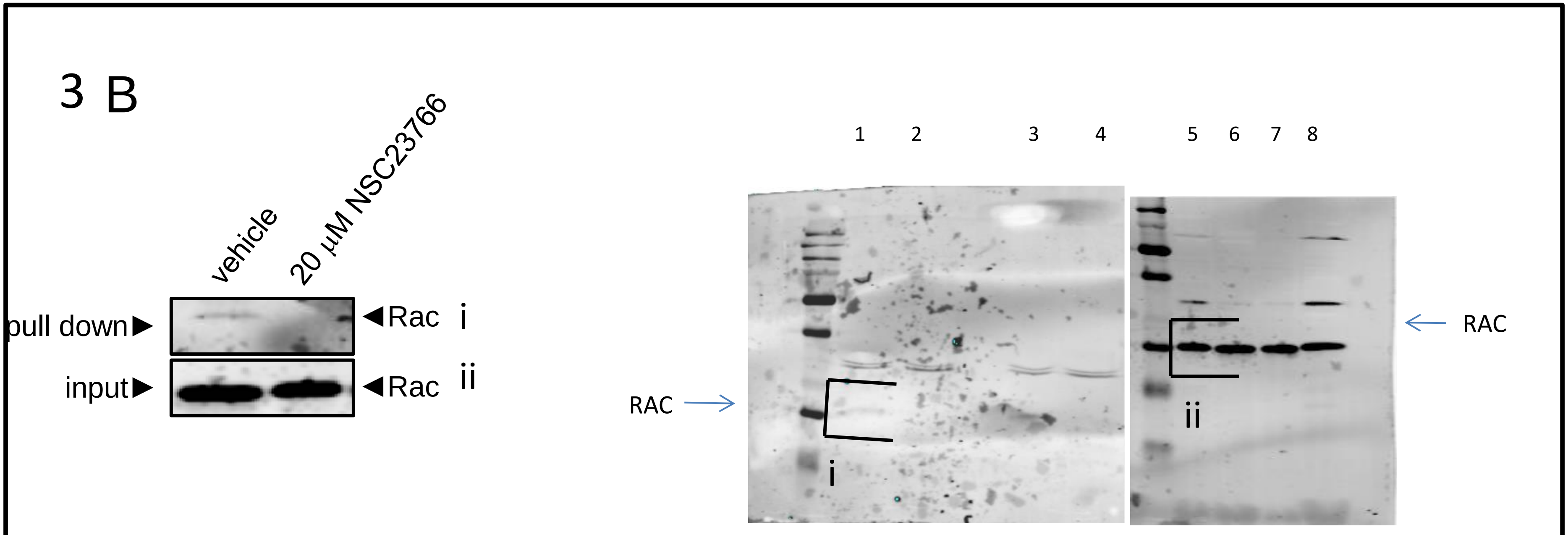

1) TSC2+/+ PD; 2) TSC2+/+ + NSC23766 (PD); 3) TSC2+/+ PD; 4) TSC2+/+ + NSC23766 (PD); 5) TSC2+/+ (I) ; 6) TSC2+/+ + NSC23766 (I); 7) TSC2+/+ (I) ; 8) TSC2+/+ + NSC23766 (I)

Supplementary Figure S4B: Full lenth blots belonging to Figures 3B. Top left corner: figure as presented in paper. Pull down and input samples were run on separate gels, which were subsequently blotted for Rac. The same incubation times and conditions were used for both blots. Lanes 1,2, 5 and 6 were used in the manuscript.

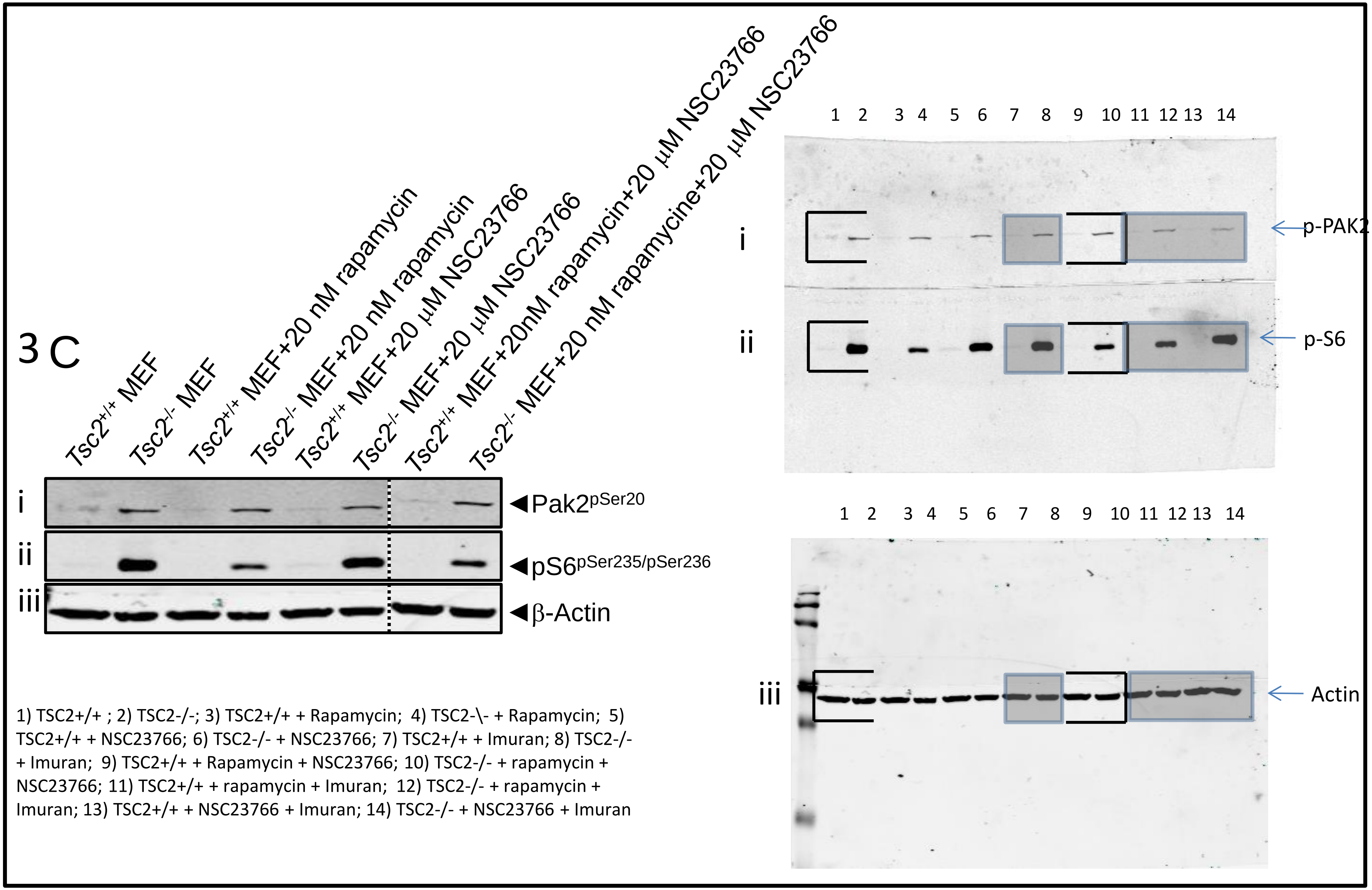

1) TSC2+/+ ; 2) TSC2-/-; 3) TSC2+/+ + Rapamycin; 4) TSC2-\- + Rapamycin; 5) TSC2+/+ + NSC23766; 6) TSC2-/- + NSC23766; 7) TSC2+/+ + Imuran; 8) TSC2-/- + Imuran; 9) TSC2+/+ + Rapamycin + NSC23766; 10) TSC2-/- + rapamycin + NSC23766; 11) TSC2+/+ + rapamycin + Imuran; 12) TSC2-/- + rapamycin + Imuran; 13) TSC2+/+ + NSC23766 + Imuran; 14) TSC2-/- + NSC23766 + Imuran

Supplementary Figure S4C: Full lenth blots belonging to Figures 3C. Left: figure as presented in paper. p-PAK2, actin and p-S6 were blotted on the same gel. The gel was cut below the 50kDa band of the marker and the upper part was blotted with p-PAK2, and the lower part was blotted with actin and p-S6. As p-S6 and actin antibodies were from different sources, we blotted them together and developed using two differently labelled secondary antibodies. In the manuscript lines 7, 8, 11, 12, 13, and 14 were not shown (indicated by dotted line and grey bars in full length blots.)

Peptide #	MOTIF	PROTEIN	UP_KIN	PSITE	Wild type MEF Σ(x1,x2,..,xn)/n	Wild type MEF σ(x1,x2,..,xn)	Wild type MEF Markov score	TSC2-/- MEF Σ(x1,x2,..,xn)/n	TSC2-/- MEF σ(x1,x2,..,xn)	TSC2-/- MEF Markov score
1	ELDQGSLCTSF	IKK beta	IKKs NAK / NIK;MAP3K7;MEKK1;_IKK_alpha	S177	6,365	2,395	2	7,02	0,13	2
2	VDLACTPTDVR	Cyclin D1	GSK3beta	T286	2,726666667	1,930964065	3			0
3	TTNEEYLDLSQ	Fibroblast growth factor receptor 2	FGFR2 (to PLCg)	Y770	4,92		1	6,295	0,995	2
4	SSLDVYDGRFL	Acetylcholinesterase	PKA(?) / nd	S164	5,595	1,323905208	4			0
5	ALVHSYMTGRR	SH2D3A	nd	Y95	8,436666667	0,217536715	6			
6	NegCtrl	Mixed peptides without STY	na					8,628	0,48217839	5
7	SptCtrl	Used for production/QC	na		8,423333333	0,474856704	6	8,643333333	0,480370228	6
8	PosCtrl	Mixed kinase substrate peptides	na		6,56	0,13	2	6,51		1
9	FDDPSYVNVQN	Shc	TRK-T3;Insulin_receptor;MAP_kinase;Fyn;Lck	Y427	7,84	0,937656654	3	7,55	0,063770422	3
10	HHIDYYKKTTN	FGF receptor 1	FGFR1 (imp for catalytic activity)	Y654	8,715	0,598240476	6	8,778333333	0,436593505	6
11	TATEGQYQQQP	Lyn	Csk_homologous_kinase	Y508	2,135	2,135	2			0
12	QGVRTYVDPFT	EphA4	EphA4	Y596	5,946666667	0,910433352	3			0
13	KTRDQYLMWLT	Phosphatidylinositol 3 kinase, regulatory subunit, alpha	Insulin_receptor	Y580	2,403333333	3,398826595	3	3,746666667	2,878950426	3
14	FMCKVYSDPQP	FGF receptor 1	ligt extracellulair FGFR1	Y280	5,695	0,005	2	5,15		1
15	TSSVLYTAVQP	PDGF receptor, beta	PDGFRbeta to PLCg	Y1009	2,95	2,95	2	1,663333333	2,352308559	3
16	NADDSYEPPPV	B-cell linker protein	SYK / SLP-76	Y96			0	4,305	2,509287748	4
17	KERRRTESINS	HAND1	PKA;PKCalpha	T107	4,796666667	3,707940429	3			0
18	ALRISTPLTGV	Retinoblastoma like 2	CDK4	T401	5,8	0,75	2			0
19	NSRPGTPSAEG	Transcription factor IIF, alpha subunit	cdk9 / TFIIFalpha_subunit	T389			0			0
20	ENTLPTPMVLQ	Mnk2	ERK ? / nd	T379			0	5,966666667	0,897935905	3
21	EPVQLTPDDED	Protein kinase C, zeta	PKCzeta	T560	6,973333333	0,695908679	3	3,68	3,68	2
22	GSGTATPSALI	Transcription factor Sp1	ERK1	T736			0	6,53		1
23	ISEGTTLKDLI	TGF beta receptor, type I	TGF_beta_receptor,_type_II	T176	7,106	0,142632395	5	7,782	0,221756623	5
24	GSAAPYLKTKF	STAT3	JAK1;c-Src	Y705	7,708333333	0,355453544	6	7,5425	0,322286751	4
25	NYQVPSPGPSH	T-Cell acute lymphoblastic leukemia 2	Erk / nd	S100	3,62		1	7,0325	0,154171171	4
26	AFEEGSQSTTI	ATM	auto	S1981	6,32		1	8,056666667	0,453161732	3
27	NGLPGSRPGSP	HIV 1 Tat interacting protein	Cyclin_B1;CDC2	S119			0	4,74		1
28	EERKASGPPKG	CRIP2	cGMP_kinase_I	S104	5,34	0,28	2			0
29	KRQYGTISHGI	Amyloid precursor like protein 2	PKC	T723	8,881666667	0,387359293	6	8,812	0,382015706	5
30	LIEPDTPGRVP	Plectin 1	CDC2	T4536	6,51		1	3,365	3,365	2
31	CGSQKYAYFNG	Connexin 43	c-Src	Y265	7,376666667	0,263986532	3	7,314	0,486275642	5
32	GVTTTTFCGTP	Protein kinase C epsilon	autophos / nd	T566	4,51	0,54	2	4,1075	2,011471787	4
33	AFRAPSIHGGS	Keratin 19	p38 MAPK / nd	S35	7,53	1,57	2			0
34	MDSSISKQALS	Syntaxin 1A	DAP kinase (linked to autophagia) / Syntaxin_1A	S188	6,14	0,43	2	7,0275	0,499818717	4
35	SRRPKSSLPPV	Myosin light chain kinase	PAK2	S1208	6,5675	0,848391861	4	6,925	0,990416579	4
36	RSRSRSPRPRG	ErbB3	Phospholipid_kinase_Phosphoinositide_kinase	S1123	7,84	0,43099884	5	8,225	0,253229145	4
37	VMVTASHNPEE	Phosphoglucomutase 3	PAK1 / nd	S64	5,995	0,305	2	7,7	0,91	2
38	PGADLSQYKMD	CtIP	ATM	S664	6,335	1,115	2	5,925	1,185	2
39	GLYSRSGSLSG	PKN	PKN	S374	7,2075	0,20716841	4	4,773333333	3,526190894	3
40	ARPPASPSPQR	Synapsin I	CDK5	S551	6,495	0,537842914	4	7,43	0,513225097	3
41	EILGDSQHADV	Fanconi anemia, complementation group D2	ATM / nd	S222	7,855	1,125	2	5,7675	1,225446347	4
42	VLAQPSTSRKR	CHK2	ATM / PKA;PKG	S19	9,051666667	0,132842848	6	9,303333333	0,270903837	6
43	ISRQHSYDNIV	Glutamate receptor ionotropic, N-methyl D-aspartate subunit 2A	CaMKII	S1291	7,89	0,248274042	5	8,078333333	0,506833854	6
44	QARANSFVGTA	3-Phosphoinositide dependent protein kinase 1	PDK1	S241	3,965	2,49259403	4	6,256	1,102734782	5
45	RIRTQSFSLQE	Nitric oxide synthase 3	PKG / AKT1	S1176	6,4575	0,717229914	4	7,6325	0,18753333	4
46	AVHEDSGDEDG	Histone deacetylase 2	CK2	S394	6,18	0,193045763	3	6,83		1
47	KEEPPSPPQSP	Heat-shock transcription factor 1	GSK3	S303	4,985	1,15140132	4	6,253333333	0,883037686	3
48	QTPPVSPAPQP	Cortactin	ERK, EphB2	S405	3,2		1			0
49	YVSILSPKEVS	Regulator of G protein signaling 19	ERK2	S151	7,886	0,639205757	5	8,428333333	0,413135033	6
50	LDRFLSLEPVK	Cyclin D1	associated with cyclin D degradation / PKA	S90	8,506666667	0,26898988	6	9,038333333	0,173341346	6

51	KPKDASQRRRS	c-Src	PKC	S12	8,675	0,132003788	6	8,686666667	0,452130758	6
52	GIWKASFTTFT	Caveolin 1	c-Src	Y14	8,051666667	0,473301055	6	8,1	0,351738539	5
53	KALPLSPRKRL	CDC6	CDK2	S54	9,121666667	0,221842036	6	8,984	0,257340242	5
54	YSQGASPQPQH	Cut like 1	CDC2	S1237	5,6525	0,465315753	4	6,905	0,518531581	4
55	LQRQASPSIVI	Ras associated protein Rab5B	CDC2	S123	5,89	1,033956801	3	8,463333333	1,274528235	3
56	NSNVFSMFEQT	Myosin light chain 2, regulatory, cardiac, slow	MLCK	S19	7,266666667	0,528919233	6	8,0225	0,621143099	4
57	AGPALSPVPPV	Bcl 2	JNK1;TNF-alpha	S87	4,49	4,49	2	6,62		1
58	EPQRRSARLSA	HMG17	Ribosomal_protein_S6_kinase_alpha_3;PKA;PKC	S25	8,791666667	0,215825289	6	8,926666667	0,2171533	6
59	IRESESTAGSF	Lck	PKC	S158	7,32	0,312089731	5	7,816666667	0,368766713	6
60	RTAKDSDDDDD	Syntaxin 1A	CK2	S14	7,125	0,413793427	4	7,584	0,186611897	5
61	RDSPLSSRLLD	HSP22	PKC	S14	7,7275	0,437171305	4	8,3025	0,517801844	4
62	YQRRASDDGKL	RAF1	PKA (neg. to Raf)	S43	6,674	0,249687805	5	7,765	0,470983015	4
63	SRKGYSRKGFD	Peptidylglycine alpha amidating monooxygenase	PKC	S930	9,42	0,253771551	6	9,208	0,185191792	5
64	GFTEESGDDEY	TCFL1	CK2	S41	9,796666667	0,447052073	6	9,671666667	0,301519301	6
65	GLAEFYHPGQE	CK2, alpha 1	CDC2	S370	3,48		1	5,885	0,375	2
66	PSPKSYENLWF	Colony-stimulating factor 3 receptor	c-fms to shc / Hck	Y814	4,68		1	6,63	0,12	2
67	VGTARYMAPEV	TGF beta receptor, type II	TGF_beta_receptor_II	Y424	5,17		1	3,08	2,37492456	3
68	AEKPFYVNVEF	BCR	Lyn, Abl BCR	Y177	6,235	0,275	2	6,05	0,85390866	5
69	FKTEGPDSD	p53	CDK7 / CK2alpha_1;p38_MAP_kinase;PKR;CDK2	S392	0		1	6		1
70	NegCtrl	Mixed peptides without STY	na							
71	SptCtrl	Used for production/QC	na		8,2425	0,32911814	4	7,61	0,504380809	5
72	PosCtrl	Mixed kinase substrate peptides	na		5,913333333	0,505459087	3			0
73	TAECPSVLELG	Crystallin beta A4	nd	S35	5,68	0,29	2	6,975	0,211719154	4
74	VLDDEYVSSFG	Protein tyrosine kinase TXK	Lyn, Fyn	Y420	7,7225	0,667134732	4	7,7325	0,478193214	4
75	TLVIAYLMMRQ	Dual specificity phosphatase 3	ZAP70_	Y138	3,085	3,085	2	6,3425	0,24190649	4
76	TIPPKYRELLA	Calpastatin	c-Src	Y205	5,223333333	0,928702799	3	6	0,19	2
77	FDAHIYEGRVI	Protein kinase C delta	c-Src / PDGF;Lyn_kinase	Y64	6,19		1	6,638	0,517470772	5
78	AEDSTYDEYEN	Cortactin	c-Src	Y486	6,6575	0,571505687	4	6,77	0,42	2
79	PGLRTYVDPHT	EphA3	EphA3	Y596	6,43	0,47	2			0
80	CSLGQYTSIGG	GABA transporter	nd	Y107	5,822	0,267761088	5	5,85	0,14	2
81	VDPMLTPEERH	Amyloid precursor like protein 2	JNK / CDC2-kinase	T736	6,88		1			0
82	SSYRRTFGGAP	Desmin	Rho_Kinase	T17	7,645	0,515	2	6,6825	0,53072474	4
83	IEQFSTVKGVE	G protein coupled receptor kinase 6	G_protein_coupled_receptor_kinase_6	T485	6,32	0,45	2	5,86	0,313793988	3
84	FNNPAYYVLEG	Inositol polyphosphate phosphatase like 1	Lyn kinase / c-Src	Y986	7,253333333	0,614563983	6	7,45	0,331119314	5
85	VTKLLTDVQLM	Heat-shock transcription factor 1	CK2	T142	3,67	0,04	2	5,22		1
86	TATNSTLPSAE	Formyl peptide receptor 1	Beta-adrenergic_receptor_kinase1	T339	3,75		1			0
87	NPCTETFTGTL	ASK1	ASK1	T+T778838	4,145	1,275	2	2,63	2,63	2
88	PGAGHYEDTIL	ALK	ALK	S1604	6,855	0,416563321	4	7,7225	0,379300343	4
89	CLSPASSGSSA	NFAT1	GSK3 ? / nd	S171	4,98	0,56	2	0		1
90	NQKYMSFTSGD	PAK3	PAK3	S139	5,893333333	0,637930682	3	7,71	0,31	2
91	PSPPPSPSQQI	Serum/glucocorticoid regulated kinase	ERK5	S78	0	0	2	0	0	2
92	PGKAESVASLT	Period circadian protein 2	CK1epsilon	S662	6,335	0,645	2	3,85		1
93	FLSSPTYRALL	Regulator of G protein signaling 19	PKC	T201	7,71	0,294278779	3	6,645	0,745	2
94	RLRQGTLRRDL	Epithelial calcium channel 2	PKCalpha	T702	7,596	0,652766421	5	7,05	0,345543051	3
95	REWHKTTQMSA	MAP3K11	MAP3K11 MLK3	T277	6,31		1	6,705	0,605	2
96	AEEKEYHAEGG	EGF receptor	c-Src	Y869	6,6	0,42	2	5,985	0,752778188	4
97	LQRSSSFKDFA	SH3 protein expressed in lymphocytes	nd	S27	7,3575	0,536534016	4	8,176666667	0,504667107	6
98	SDEVQSPVRVR	PCTAIRE protein kinase 1	CDK_5	S95	2,705	2,705	2			0
99	PFRRPSTYGIP	TFII-I	PRKG1	S412	8,302	0,508936145	5	8,3225	0,269849495	4
100	FMRRTSLGTEQ	EP4 receptor	PKA	S222	5,03	1,18	2	5,245	0,845	2
101	PGGDRSPSRML	Runt related transcription factor 2	Erk(?) / nd	S451	4,2	0,24	2	5,93		1
102	PQSDPSVEPPL	p53	CK1alpha_1	S9	4,695	0,945	2	2,87	2,227599605	3

103	GSPSIRCSSVS	SMAD3	MAP3K7 / TAk1	S422	2,37	3,351686143	3			0
104	MRKKISNAQLQ	Cullin 5	PKA	S730	5,8		1			0
105	RLRTHSIESSG	MAP2K4	AKT1	S80	5,9		1	4,38		1
106	PSPPQSPRVEE	Heat-shock transcription factor 1	MAPK	S307	2,58	2,58	2	2,525	2,525	2
107	MIHNRSKINLQ	HMG CoA reductase	PKA	S872	8,13	0,370243163	5	7,87	0,234008547	5
108	AIRRASTIEMP	Phospholamban	PKA	S16	5,91	1,245993579	4	6,356666667	0,664596787	3
109	RRRAVSMDNSN	Forkhead box protein O3A	AKT1	S253	5,47		1	2,42	2,42	2
110	VPSYDSFDSED	C ets 1 protein	CaMK2	S285	7,898	0,448972159	5	7,748	0,40076926	5
111	RSRHSSYPAGT	BCL2 antagonist of cell death	AKT	S75	7,645	0,693006734	6	7,588333333	0,498979514	6
112	GFTRKSVRKAQ	Protein phosphatase 2, regulatory subunit B56, alpha	PKR	S28	8,278333333	0,228940798	6	8,088	0,233443783	5
113	HAVRESQVELR	SNARE associated protein	PKA	S50	3,44	2,445581049	3	6,026666667	0,118977122	3
114	LRRRLSDSNFM	Synapsin I	CaMKI;PKA	S9	6,16		1	7,27	0,675327081	3
115	LLRSGSSPNLN	E1A binding protein p300	PKCdelta	S89	7,97	0,514703798	5	7,922	0,731447879	5
116	SEEPDSRGGSP	eIF-2B epsilon	GSK3	S540	3,69	1,175273018	3	5,89		1
117	RRRAASMDSSS	AFX 1	AKT1	S196	6,72	0,797684148	4	6,51	0,01	2
118	DAPPLSPYPFV	Retinoblastoma like 2	CDK4	S1035	8,92	0,318538328	6	9,566666667	0,139004396	6
119	LPGALSPLYGV	Paxillin	JNK1	S178	6,815	1,007980655	6	7,201666667	0,565815537	6
120	SSTPVSPLHHA	ETS variant gene 1	ERK / ERBB2	S146	6,172	0,843336232	5	7,495	0,446682214	4
121	PSRKGSGFGHR	Connexin 32	PKC	S229	8,261666667	0,278473019	6	7,886666667	0,428978114	6
122	RLRRDSKEANA	RAS related associated with diabetes	PKA, PKC,CKII	S257	2,16	2,211345744	4	4,07	3,267486292	3
123	EVFDFSQRRKE	NFE2L2	PKC / nd	S40	7,482	0,635496656	5	7,36	1,032724552	5
124	EDGTGSPQLNN	Beta arrestin 1	GRK2/BARK / ERK1;ERK2	S412	6,22		1	4,75		1
125	SSPRHSEAATA	Heterogeneous nuclear ribonucleoprotein D	PKA	S87	6,04	0,99	2	3,506666667	2,481213861	3
126	VKRRPSPYEME	T cell acute lymphocytic leukemia 1	erk1 / T_cell_acute_lymphocytic_leukemia_1	S172	7,333333333	0,357055862	3	6,931666667	0,653692503	6
127	KFRTPSFLKKN	Adducin 3	PKCalpha	S693	7,614	0,229486383	5	7,706	0,301370204	5
128	STPAPSRTASF	ATP citrate lyase	GSK3	S450	8,485	0,276691766	6	7,298333333	0,893633345	6
129	EGDEIYEDLMR	VAV1	Lck	Y174	8,22	0,513906606	6	8,403333333	0,629620697	6
130	IESDIYAEIPD	Focal adhesion kinase 2	FAK2	Y402	4,8825	2,904775163	4	6,635	0,505	4
131	ILDREYYSVQQ	Ron	Ron&c-Met (imp for catalytic act.)	Y1238	6,215	0,215	2	0		1
132	TAPEYYAPEVH	Titin	nd	Y24917	5,57	0,33436507	3	6,58	0,08	2
133	CSGLSSQSDIL	BRCA1	ATM	S1387	1,933333333	2,734146221	3	3,5725	2,072310003	4
134	NegCtrl	Mixed peptides without STY	na							
135	SptCtrl	Used for production/QC	na		8,478333333	0,480049187	6	8,315	0,636468119	6
136	PosCtrl	Mixed kinase substrate peptides	na		5,228333333	2,256999606	6	3,72	2,771726297	3
137	IGEGTYGTVFK	Cyclin dependent kinase 5	ABL	Y15	6,965	0,467894219	4	7,186	0,632664208	5
138	FEGFSYVNPQF	Protein kinase C alpha	SYK	Y658	6,78	0,407615014	4	7,82	0,67	2
139	TSLAQYDSNSK	Bone marrow kinase BMX	BTK;ITK;Tec	Y216	4,58	0,06	2	6,38		1
140	SANAIYSLAAR	CBL	tyrosine kinases EGFR	Y774	0		1	5,31		1
141	LTIDRYLAIVH	CCR2	JAK2	Y139	7,855	0,246018292	4	7,555	0,405801676	4
142	PSEEGYQDYEP	Synuclein alpha	SYK	Y136	6,79	0,446631839	5	7,462	0,70799435	5
143	MEDYDYVHLQG	p130CAS	phosph as consequence of rac activation (src?) / FAK	Y666	4,455	2,596155812	4	5,01	0,949368211	4
144	IDAFSDYANFK	Protein tyrosine phosphatase, receptor type, alpha	Src, Fyn, Yes / nd	Y798	6,876	0,547415747	5	6,4625	0,516109242	4
145	PVVTCTPSCTA	c-Fos	MAPK	S374	2,67		1	5,09		1
146	RSRRLTFRKNI	Endothelial differentiation gene 1	AKT	T236	6,27		1			0
147	ENPNFTGKKME	Crystallin, beta B2	nd	T118	6,635	0,455	2	5,48	1,90579817	3
148	LGSQSYEDMRG	CD19	Lyn_kinase;Bruton's_tyrosine_kinase	Y531	5,696666667	0,989421829	3	5,855	0,583159498	4
149	SSSPSTPVGSP	Transcription factor 3	MAPK	T355	2,4375	2,823387814	4	0	0	2
150	TLQTRTQEGSL	DNA-dependent protein kinase catalytic subunit	DNA-dependent_protein_kinase_catalytic_subunit	T2620	3,426666667	2,57295334	3	5,235	1,025	2
151	MSTESMIRD	TNF alpha	Casein_Kinase	S2	5,963333333	0,322937903	3	5,64	0,360832371	3
152	ERGQEYLILEK	Tec tyrosine kinase	Tec_tyrosine_kinase	Y206	7,26	0,547768199	4	7,36	0,4	2
153	GYLVDSVAKTI	MAP2K6	MLK3 / nd	S207	7,4675	0,546917498	4	7,268	0,433976958	5
154	RYHGHSMSDPG	Pyruvate dehydrogenase complex, E1-alpha polypeptide 1	Pyruvate_dehydrogenase_kinase,_isoenzyme_1_PDP	S293	6,1		1	7,27	0,75	2

155	HFTMRSPFKCD	Glutamate receptor ionotropic, N-methyl D-aspartate subunit 2A	CDK5	S1232	7,42	0,663438015	4	7,01	1,18	2
156	FELAFSLDQPD	Mnk2	nd	S27	6,835	0,209582919	4	7,0175	0,402142699	4
157	SILPFTPPVVK	SMAD2	ERK	T8	7,91	0,352230985	3	7,223333333	0,343931518	3
158	PLSQETFSDLW	p53	CK1;DNA-dependent_protein_kinase_catalytic_subunit_ATR	T18	9,13	0,47003546	6	8,721666667	0,309807288	6
159	AGSRLTLSLRG	NPR-B	autophosphorylation / nd	T516	7,52	0,517880295	6	7,754	0,27426994	5
160	PTHSPYAQPSS	p73	ABL	Y99	1,005	1,005	2	0	0	2
161	AQKSSSPAPAD	Ras-GTPase-activating protein SH3-domain-binding protein	nd	S232	3,512	2,881606496	5	0		1
162	QEEGSSQGEDS	SMC1	ATM	S957	6,9475	0,450187461	4	7,053333333	0,247969532	3
163	LRRSLSRSMSQ	Telethonin	nb / Titin	S157	4,28	2,549941176	4	0		1
164	YSPPGSPPPGD	Ribosomal protein S6 kinase alpha 4	p38MAPK / MAPK14;MEK1	S347	6,3225	0,803939519	4	6,386666667	0,750880964	3
165	VGKIFSNVRIN	Caveolin 1	CK2	S88	7,065	0,626438345	4	7,365	0,413370294	4
166	FDGPLSPPLSI	Neurogenic differentiation factor 1	EphB2	S274	5,195	1,304259049	6	6,764	2,213401003	5
167	GPRRRSRKPEA	Centromeric protein a	Aurora_kinase_B	S7	7,143333333	0,376637167	6	7,096	0,653011485	5
168	GQTPSSSSIPS	MRVI1	cGKI-beta	S696	0		1	1,215	1,215	2
169	DIEVESDEEQP	MAX protein	CK2	S11	7,852	0,644279442	5	7,368	0,562437552	5
170	KFSKFSLYKQL	PLD1	PKCalpha	S561	6,81	0,519374624	4	5,22	2,667995502	5
171	PTTPLSPTRLS	Lamin B1	CDC2	S23	7,6975	0,543984145	4	6,946666667	0,18372685	3
172	GNIYISPLKSP	Retinoblastoma 1	pRB_kinase	S807	8,684	0,190850727	5	8,633333333	0,303131801	6
173	KGGAASGSDSA	Peptidyl prolyl cis/trans isomerase nima interacting, 4	CK2	S19	2,18	2,017037431	5	3,425	1,865	2
174	AVRLRSSVPGV	Vimentin	Rho_kinase	S72	8,153333333	0,411487815	6	7,973333333	0,574504618	6
175	NIIHGSDSVES	Nucleoside diphosphate kinase A	Nucleoside_diphosphate_kinase_A	S120	6,306	0,331638357	5	5,7325	1,260800837	4
176	EPNPNSPANSQ	Ubiquitin conjugating enzyme E2A	CDK2	S120	2,68	2,68	2	5,76		1
177	NQNSSSDSEAE	T-cell transcription factor 4	CK2	S60	7,245	0,253031619	4	7,646666667	0,27716822	3
178	ELRRMSDEFVD	BCL2 antagonist of cell death	PKA;AKT1	S118	5,926666667	0,77172246	3	6,5025	0,479811161	4
179	IRYIESLQELL	Myogenic factor 5	CK2	S130	9,088333333	0,607986202	6	9,031666667	0,213652106	6
180	LPSTQSLNIKS	Myocyte specific enhancer factor 2C	Big_MAP_kinase_1	S387	6,87	0,486703195	5	7,292	0,47097346	5
181	EEKQKSDAEED	Calnexin	CK2	S564	7,55	0,595063022	6	7,29	0,437949769	5
182	LVDTASPSPME	Cyclin B1	Polo_like_kinase	S126	3,606666667	2,557203334	3	3,45	3,492699529	4
183	YGGLTSPGLSY	Keratin 8	p42_kinase	S431	8,332	0,233700663	5	8,336666667	0,469100795	6
184	QQLQLSPLKGL	Ribonucleotide reductase M2 subunit	CDC2	S20	2,8625	2,871710074	4	3,47	2,556041862	4
185	GTRRGSPLLIG	GFAT	PKC	S205	8,118333333	0,433105709	6	8,085	0,300596629	6
186	GMDYRSCILRP	Thimet oligopeptidase 1	PKA	S643	8,301666667	0,27491918	6	8,036	0,208096132	5
187	DSGIHSGATTT	Beta-catenin	GSK3beta	S37	2,95	2,091426945	3	1,746666667	2,58412246	3
188	ASRMESTGVMG	Adenylate cyclase 3	CaMK-II	S1076	5,21		1	6,5225	0,590778089	4
189	RKKKVSSTKRH	Cytohesin-1	PKCdelta	S394	10,17	0,442116124	6	10,794	0,429446155	5
190	AERRNSILTET	CFTR	PKA;PKG	S660	2,6075	2,65606264	4	6,102	0,254589866	5
191	AESGKSASTEV	Axis inhibitor 1	GSK3beta	S614	8,5725	0,789948574	4	9,016	1,263813277	5
192	SDSLSSPTLLA	c-Fos	EphB2;ERK2	T325	7,354	0,52591254	5	7,2775	0,504300258	4
193	PELARYLNRNY	Hrs	tyrosine kinases / EGFR	Y329			0			0
194	INGNNYVYIDP	KIT	KIT to shc/src	Y568	4,955	0,195	2	5,015	1,085	2
195	EPENDYEDVEE	Hematopoietic cell specific LYN substrate 1	Lyn / nd	Y378	6,33	0,35	2	3,3	3,3	2
196	PCTTIYVAATE	SLAM	Fyn	Y307	4,88		1	5,513333333	0,314783876	3
197	GVITTTPTPPG	Oncogene Jun-B	JNK;MAPK	T102			0	4,09		1
198	NegCtrl	Mixed peptides without STY	na							
199	SptCtrl	Used for production/QC	na		7,858333333	0,439447254	6	8,726666667	0,944610443	6
200	PosCtrl	Mixed kinase substrate peptides	na		3,406666667	2,75208204	3			0
201	AQDEFYRSGWA	Csk	Csk	Y184	6,823333333	0,200720923	3	6,585	0,415	2
202	IKDDEYNPCQG	Fgr	Fgr	Y412	4,1	1,37	2	3,536666667	2,509453238	3
203	GGDDIYEDIIK	VAV2	lck / nd	Y172	8,79	0,311822599	6	8,833333333	0,765716803	6
204	NGRPDYIIVTQ	Aryl hydrocarbon receptor	Src /auto	Y378	5,53	0,65	2	4,286666667	1,746812971	3
205	SFLQRYSSDPT	EGF receptor	EGFR to Grb2	Y1069	0		1	7,03		1
206	YDKEYYSVHNK	Hepatocyte growth factor receptor	c-Met (catalytic)	Y1235	7,04	0,451884941	3	6,804	0,442520056	5

207	PGMKIYIDPFT	EphB1	EphB1	Y594	3,5025	2,310620858	4	3,543333333	1,941834757	6
208	TLSTLYPSTSS	STAM1	EGFR? /nd	Y198	3,73		1	2,68		1
209	SASPYTPEHAA	p73	Cyclin_A;Cyclin_B;CDC2;CDK2;Cyclin_E	T86	5,075	0,255	2	6,693333333	1,46000761	3
210	IRRASTIEMPQ	Phospholamban	Ca2+Calmodulin_dependent_protein_kinase	T17	4,825	0,165	2	5,9575	3,204250107	4
211	VVANGTGTQGQ	Beta glucuronidase	Beta-G1	T422	5,685	0,595	2	7,75	1,78	2
212	DGKEIYNTIRR	RasGAP	EGFR;Lck nota bene negatively regulates GAP	Y460	5,216666667	0,918126111	3	2,27	2,27	2
213	RPRSCTWPLQR	Forkhead box protein O3A	Serum/glucocorticoid_regulated_kinase;AKT1	T32	8,058333333	0,902430361	6	7,056666667	0,50910597	6
214	GDAAETPPRPR	MEK1	CDK5	T286	4,553333333	3,288569429	3	4,02		1
215	AKYMETVKLLD	Vaccinia related kinase 1	nd	T305	7,993333333	0,634262476	6	8,388	0,646541569	5
216	NDDDVYRSLEE	VAV2	Tec (EGFR) / EGFR	Y142	7,036	0,953742104	5			0
217	SHLRNSPEDKR	Eukaryotic translation initiation factor 4E binding protein 1	Dual-specificity_tyrosine_phosphorylation-regulated_kinase_2	S101	6,5	0,73774431	3	7,423333333	0,913248171	3
218	DPRLLSPQQPA	Myocyte specific enhancer factor 2D	ERK5	S180	2,305	2,305	2	6,25	6,25	2
219	EKKEESEESDD	Ribosomal phosphoprotein large P2	nd	S102	5,14	1,75097687	5	10,74		1
220	TGLYKSQRPCV	BTEB2	PKC	S153	0		1	1,736666667	2,456017553	3
221	LETVSTQELYS	CHK2	CHK2	T68	7,676	0,421074815	5	6,88	0,581205643	4
222	TVAENTADELA	AF6	BCR-kinase	T893	6,012	0,825430797	5	5,41	0,15	2
223	HPPVLTPPDQE	Protein kinase C, beta-1	autophos / nd	T641	2,79	0,79	2	5,556666667	0,721587756	3
224	PATDLYQVPPG	p130CAS	c-Src	Y165	4,62	0,1	2	6,41		1
225	SPHNPISSVS	SMAD1	Activin_A_receptor,_type_I_TGF_beta_receptor,_type_I	S463	6,51	0,08	2	6,08		1
226	GATMKTFCGTP	AKT1	PDK1	T308	6,396666667	0,315101395	3	4,45	2,636218883	4
227	SAQGSDVSLTA	HLA-B	nd	S359			0	6,765	0,255	2
228	DIDNYSEEEEE	Phosphofurin acidic cluster sorting protein 1	CK2	S278	7,542	0,68045279	5	7,931666667	0,379447698	6
229	VPKRKSLVGTP	p21 activated protein kinase 6	nd	S560	7,448333333	0,413860551	6	6,696	1,007543547	5
230	GSRRSSAIGIE	RAP1 GTPase activating protein 1	PKA (neg. to Ras)	S499	0		1	5,133333333	3,637529687	3
231	WPWQVSLRTRF	Plasminogen	nd	S597	6,09		1	6,43	0,63	4
232	PPVPATPYEAF	SPIB transcription factor	ERK1	T56	8,414	0,759489302	5	8,258333333	0,508377047	6
233	LSRHSSPHQSE	Glycogen synthase 1	GSK3ß / CK2alpha_1	S653	6,0025	0,464132255	4	6,895	0,175	2
234	YRERMSSNRPN	Meprin,beta subunit	PKC	S687	6,655	0,197040605	4	6,525	0,653509755	4
235	DDLMLSPDDIE	p53	Homeodomain_interacting_protein_kinase_2	S46	6,415	0,643603139	4	6,783333333	0,337276675	3
236	KQQPGSPRRIS	Retinoblastoma like 1	nd	S975	8,926666667	0,299592316	6	8,516666667	0,455582655	6
237	RVRASSDGEGT	IRS 1	CK2beta	s330	1,745	1,745	2	4,6825	1,001807741	4
238	PESIHSFIGDG	mTOR	mTOR	S2481	6,99	0,348425028	3	6,4475	0,506871532	4
239	WKTRLSYFLQN	RGS2	PKGIalpha	S46	8,271666667	0,271625764	6	8,515	0,393033077	4
240	AGALASSSKEE	Cystatin 4	nd	S21	7,334	0,107814656	5	7,56	0,815434445	6
241	SVRRASSADDI	Potassium channel, voltage gated subfamily H, member 2	PKA	S285	7,21	0,804288505	5	7,615	0,978991828	4
242	LRRQHSYDTFV	NR2B	CaMKII;PKC	S1303	7,465	0,434961684	6	7,414	0,387071053	5
243	MQPDNSSDSDY	CD5	? / PKA	T434	8,2	0,814902857	6	8,3075	0,730971101	4
244	LSRMGSLRAPV	E2F transcription factor 1	CHK2	S364	3,384	2,937744713	5	4,89	0,985122666	3
245	PEFPLSPPKKK	Stathmin 1	p34cdc2	S38	8,016666667	0,628163107	6	8,251666667	0,446632461	6
246	RERLASTNDKG	Estrogen receptor, alpha	AKT2	S167	2,29	2,698944238	4	4,64	2,184162692	6
247	PEKRASPSKPA	Microtubule associated protein 4	CDC2_kinase	S787	2,6725	1,545645092	4	4,1525	2,461263649	4
248	GRLRKSLPSLL	Complement component 5 receptor 1	nd	S314	8,266666667	0,439987374	6	8,446	0,670450595	5
249	LARRPSYRKIL	Activating transcription factor 1	CaMKII / CaMKI;PKA;S6K	S63	9,924	0,191791554	5	9,424	0,477434812	5
250	TRVPPSRRGPD	Alpha 2A adrenergic receptor	PKC	S232	9,138333333	0,206754014	6	8,4325	0,501765633	4
251	FWSTLSPIAPR	ELK 1	p38MAPK / MAPK14;p46SAPK;p54SAPK	S383	8,866666667	0,076084748	6	7,996	0,590071182	5
252	GNGYISAAELR	Calmodulin 1	CK2	S82	8,188333333	0,416629998	6	7,518	0,381439379	5
253	NKARLSITGSV	Glutamate receptor ionotropic, AMPA 4	nd	S862	9,675	0,385605584	6	9,038333333	0,631939255	6
254	GFSRKSHTFLP	CNPase	PKC	S9	10,025	0,336043152	6	9,33	0,606575085	6
255	RLKSISERLSV	Aquaporin 0	PKA	S331	9,151666667	0,408020288	6	8,708333333	0,669114257	6
256	GSEGDSESGEE	Serum response factor	P90RSK / MAPKAP/ CaMK4 / CK2	S83	8,4875	0,223425043	4	8,052	1,084424271	5
257	ADGMLTFCGPK	Crystallin, alpha A	nd	T140	7,325	1,715	2	1,28	1,28	2
258	DNEYFYVDFRE	FMS-related tyrosine kinase 3	FLT - (catalytic) FMS-related_tyrosine_kinase_3	Y591	5,3325	3,095217076	4	7,026	0,581914083	5

259	TTVELYSLAER	Protein kinase C, theta	Lck	Y90	6,03	0,952575456	3	2,063333333	2,917993984	3
260	PRQLNYIQVEL	FRS3	TrkB couples to shc	Y417	5,4		1	5,063333333	2,014982768	3
261	QRLFRSPSMPC	CDC 25B	Chk1/Chk2 Eg3_kinase	S342	3,136666667	2,240124005	3	0	0	2
262	NegCtrl	Mixed peptides without STY	na							
263	SptCtrl	Used for production/QC	na		7,514	0,278539046	5	7,864	0,665329993	5
264	PosCtrl	Mixed kinase substrate peptides	na		0	0	3	0,5575	0,965618325	4
265	HPFLRRNSGC	Aralkylamine N-acetyltransferase	PKA	S205	7,565	1,435	2	5,62		1
266	AVSEEYLDLRL	FGF receptor 4	FGFR4 to PLC	Y754	7,23	0,568272235	6	7,8525	0,14703316	4
267	TIEDSYTKICS	Ras-related protein	EphB2	Y66	7,2		1	7,23	1,09	2
268	TDNEDYEHDDE	3BP2	SYK	Y174	7,1275	0,772508091	4	7,1	0,486894924	3
269	MTRDIYETDYY	IGF-I receptor	Insulin_receptor	Y1185	7,7475	0,362999656	4	7,832	0,637350767	5
270	IKEDVYLSHDH	Protein tyrosine kinase 6	Lck/Protein_tyrosine_kinase_6	Y342	6,3825	0,609974385	4	6,86	0,358161975	5
271	DESVDYVPMLD	PDGF receptor, beta	PDGFR auto to PI3K / SHP2	Y751	5,906666667	0,471192341	3	5,77	1,278775456	3
272	KEDPIYDEPEG	Docking protein 1	Insulin_receptor	Y362	6,7		1	4,42	4,42	2
273	QELRKTFKEII	Melanocortin 4 receptor	PKA_GRK	T312	8,1475	0,455871418	4	8,268333333	0,582563778	6
274	SFAPSTPLTGR	Retinoblastoma like 1	CDK4	T369	1,29	2,029837432	5	3,065	3,065	2
275	LKGPGTPAFPH	C/EBP Epsilon	MAPK	T74	7,24	0,04	2	7,913333333	1,001509971	3
276	MAKRNTVIGTP	Serine/Threonine protein kinase4	Serine/threonine_protein_kinase_3	T180			0	7,17		1
277	LNRIQTQIRVV	ATP2B1	PKC	T1117	7,41	0,741754676	5	7,958	0,281382302	5
278	RGDVFTMPEDE	Vitronectin	CK2	T69	5,23		1	4,88	2,58	2
279	NPCAYTPPSLK	Protein phosphatase 1, regulatory subunit 1B	CDK5	T75			0	6,92	1,61	2
280	QQTNDYMQPEE	Actinin alpha 1	FAK	Y12	7,06		1	6,214	0,975327637	5
281	IGADSSEEKFL	Statherin	nd	S22	8,513333333	0,345478734	6	8,851666667	0,466955268	6
282	EKCSDSQSWED	Tumor protein p53 binding protein 1	ATM	S784	8,486666667	0,195334471	6	8,635	0,255065351	6
283	GSPSVRCSSMS	SMAD2	Activin_receptor_like_kinase_4_TGF_beta_receptor,_type_I	S464	4,16	2,942481946	3	4,24	0,97	2
284	QTARKSTGGKA	H3 histone, family 3A	JNK2	S29	7,868	0,224802135	5	7,734	0,560271363	5
285	IPRRTTQRIVA	Collapsin response mediator protein 2	Rho-kinase	T555	6,8975	1,097323448	4	7,3375	0,938972177	4
286	IPTGTTPQRKS	Kinesin like protein 1	p34cdc2;cyclin_B	T927	6,42	0,779384372	5	7,845	0,275	2
287	MSSPPPARSG	MAPK12	p38MAPKgamma / MAPK12	S3	6,595	0,645	2	7,25		1
288	HQYFMTEYVAT	ERK5	mkk5 / nd	T218			0	2,99		1
289	GYMPMSPGVAP	IRS 1	mTor / nd	s616	7,556	0,375691363	5	7,99	0,508684578	5
290	PEPYASPPQPG	Steroidogenic factor 1	ERK2	S203	3,562	2,920468456	5	1,963333333	2,776572627	3
291	KKWKQSVRLIS	Death associated protein kinase 1	Death_associated_protein_kinase_1 / is Ca-Calmodulin regulated	S308	6,3425	0,900982103	4	6,555	0,785	2
292	TSSSSSPPGTP	CCAAT/Enhancer binding protein, beta	p90RSK	T266	3,34	2,26	2	2,98	2,98	2
293	IKQGEKHSA	Phosphoinositide-3-kinase, catalytic subunit, gamma	nd	S1100	1,79		1	5,18		1
294	LLMIISQKDTF	Telomeric repeat binding factor 1	ATM	S219	7,358	0,330901798	5	7,535	0,408686922	4
295	DSPPASPLQRQ	Syntrophin alpha 1	p38MAPKgamma/MAPK12	S193	3,9675	3,105546449	4	5,35		1
296	ARKKFSGLEIS	Sucrase isomaltase	? / PKA	S7	7,596	0,334879083	5	8,08	0,256904652	4
297	LSPLPSQAMDD	p53	DNA-dependent_protein_kinase_catalytic_subunit_208900_ATR	S37	6,568	1,179955931	5	7,24	0,465295605	4
298	TQRQNSAQLGM	CDC 25A	Cell_cycle_checkpoint_kinase	S123	5,885	0,525	2	7,335	0,705	2
299	SSRRQSVLVKS	Glutamate receptor, ionotropic kainate 2	PKA	S715	8,518	0,381701454	5	8,156	0,130015384	5
300	SALLSQISS	NFKB3	IKKbeta	S536	6,01	1,235826309	3	6,974	0,322279382	5
301	RPRTTSFAESC	Glycogen synthase kinase 3 beta	Akt/PKB / PKA	S9	4,96		1	2,095	2,095	2
302	GISQESSEEEQ	High mobility group AT-hook 1	CK2	S102	7,19	0,538330753	4	7,28	0,546900357	6
303	LNRTLSMSSLP	Glycogen synthase 1	PKA	S8	7,59		1	7,16	0,435775171	4
304	RGQRDSSYYWE	RAF1	PAK1;PAK3	S338	8,678333333	0,14088017	6	8,71	0,180831413	6
305	VNPSISPAHGV	SRC1	ERK2	S395	6,86	0,51	2	6,94	0,389444048	3
306	AHRKGSSSNEP	c-Fos	MAPkinase;EphB2;ERK2	T232	5,825	0,515	2	5,36		1
307	AGIQTSFRTGN	DDX5	PKC	S557	8,163333333	0,319617827	6	8,1325	0,466120961	4
308	AVGSLSGAEGV	MAZ	CK2	S480	6,176666667	0,863416984	3	6,26	1,053494186	4
309	EAKRKSPKKKE	Coilin	CDK2-cyclin_E	S184	8,513333333	0,910762806	6	8,614	0,305129481	5
310	TTTAPSLSGKG	Beta-catenin	Gsk3 (on T41) / CK1	S45	4,023333333	2,865034419	3			0

311	TACTPSDGPGG	Opioid receptor	Beta-adrenergic_receptor_kinase1	S363			0	0	0	2
312	GDSDASSPRSN	Myogenic differentiation antigen 1	Cyclin_dependent_kinase	S200	5,05	0,746904724	3	3,155	3,155	2
313	PVARTSPLQTP	Bcl 2	JNK1	S70	8,326666667	0,61239058	3	6,79	0,532353266	4
314	KVRFNSVSSYS	Nitric oxide synthase 1	PKG / CAMKIIalpha;CAMKIalpha;CAMKIV;PKA;PKC	S852	8,09	0,315277655	5	7,851666667	0,309053214	6
315	LSPSPSSRVTV	Lamin B1	PKC	S395	8,74	0,445600718	5	8,643333333	0,565734527	6
316	DTLSDSDDEDD	N-myc	CK2	S263	7,921666667	0,503501627	6	7,392	0,320711709	5
317	RSGLCSPSYVA	c-Myc	JNK1	S62	8,3	0,389717847	5	8,272	0,593578975	5
318	ERKSSSSSEDR	B-Raf	AKT1	S427	2,8425	2,898934071	4	5,575	0,877909449	4
319	HSLPFSLPSQM	CBL	PKC	S619	7,303333333	0,50341059	3	7,565	0,732114517	6
320	GTGDTSSEEDE	Transcription factor IIA, 1	CKI / CK II / TFIID;CK2	S280	7,37	0,328207252	5	7,41	0,284165445	4
321	VNLINYQDDAE	Beta-catenin	FER;Fyn	Y142	6,833333333	0,408275506	3	6,775	0,465	2
322	GWMVHYTSKDT	Protein kinase C, mu	c-Src;ABL	Y432	6,275	0,22961925	4	7,425	0,765196053	4
323	STEPQYQPGEN	c-Src	Csk;Fgr_PTPase	Y530	5,865	0,825	2	2,83	2,83	2
324	FTRRASVCAEA	Protein kinase, cAMP dependent, regulatory, type II, beta	Protein_kinase,_cAMP_dependent,_regulatory,_type_II,_beta	S114	2,815	2,815	2	0		1
325	MPDNLYTFVLK	LNK	ZAP70	Y273	3,1	3,111623371	4	4,01	2,846412947	3
326	NegCtrl	Mixed peptides without STY	na							
327	SptCtrl	Used for production/QC	na		9,306666667	0,384649913	6	9,23	0,324756319	6
328	PosCtrl	Mixed kinase substrate peptides	na		3,1425	3,148955184	4	4,48		1
329	ETNNDYETADG	CD32	Lyn;Blk;Fyn;SYK	Y279	5,955	0,125	2	6,936	0,237957979	5
330	EELAEYAEIRV	SIGLEC4A	Fyn	Y620	6,01		1	7,2	0,07	2
331	VYESPYSDPEE	ZAP70	ZAP70	Y319	8,63	0,42	2	8,716	0,246300629	5
332	MNEVTYSTLNF	Carcinoembryonic antigen-related cell adhesion molecule 1	MLK2 / MAP3K10	S508	2,59		1	5,47		1
333	TAEPDYGALYE	Phospholipase C, gamma1	Lyn	Y771	8,548	0,538159828	5	8,986666667	0,403553659	6
334	LEDNDYGRAVD	AKT1	c-Src/RTPK	Y326	4,1	1,61	2	6,053333333	0,919142112	3
335	LVNRHYAKISD	ZAP70	autophosporylation / SHC	Y474	5,735	0,595	2	3,055	3,055	2
336	LRRGEYPDYQQ	Crystallin gamma B	nd	Y63	6,113333333	1,253537217	3	5,32	1,14	2
337	ESRASTFCGTP	Protein kinase C delta	PDK1 / Lck	T507	2,145	2,145	2	0		1
338	LLNVFTPQKTL	MAPK10	CDK5	T131	7,333333333	0,665624185	6	7,626	0,71673147	5
339	CERPLTQEELL	TCFL1	CAMKII	T168	4,64	2,283873902	5	5,5625	0,586232676	4
340	EDSTYYKASKG	FAK	c-Src	Y577	7,808	0,76229653	5	8,091666667	0,383945164	6
341	LALSLTADQMV	Estrogen receptor, alpha	p38MAPK MAPK14	T311	5,345	0,795	2	5,84	0,535972636	3
342	IEQFSTVKGVN	G protein coupled receptor kinase 5	G_protein_coupled_receptor_kinase_5	T485			0	6,19		1
343	LTTGGTLKISD	LKB1	LKB1	T189			0	0	0	2
344	RRGDSYDLKDF	VAV1	ZAP70 / PKA	Y441	7,49	0,06	2	7,3	0,443001129	4
345	EPLPPSYVACS	Erythropoietin receptor	JAK2	Y504	3,765	0,635	2			0
346	ASRPSSSRSYV	Vimentin	PAK	S26	8,242	0,348218322	5	8,3325	0,727886495	4
347	PERRDSQDGSS	LASP1	cGKIalpha;cGKIbeta;cGKII;cAK	S146	3,292	2,79741595	5	4,005	2,556917089	4
348	RGRLRSADSEN	MEKK3	PKA	S337	4,9125	2,985074329	4	4,196666667	2,783646689	3
349	KKKICTRKPRF	Metabotropic glutamate receptor 1	PKC	T695	10,42	0,5483308	6	9,868333333	0,672716301	6
350	ESLESTRRILG	SNAP23	PKCalpha	T24	0		1	6,693333333	0,898344156	3
351	FPAPQTPGRLQ	ADAM 17	EphB2	T735			0	5,276666667	1,162076685	3
352	KDGNGYISAAE	Calmodulin 1	CK2	T118	7,41	0,16	2	6,605	0,180762275	4
353	RTAASSLALVS	Uridine nucleotide receptor	nd	S334	5,313333333	1,25947961	3	6,44		1
354	SDTVTSPQRAG	c-Src	Cdk5	S75	2,27	2,27	2	0		1
355	RKRRWSAPESR	Ataxin 1	Akt / ATK1	S776	8,641666667	0,212557182	6	8,735	0,308693483	6
356	KKRKRSRWNQD	Splicing factor 1	PKG-1	S20	9,343333333	0,131993266	6	8,916666667	0,161932771	6
357	SAIRQSPSPPP	cAMP-specific 3',5'-cyclic phosphodiesterase 4A	nd	S447	5,29	1,31828171	3	3,725	3,725	2
358	RTRTDSYSAGQ	mTOR	PI3K pathway (autophos?) /AKT	S2448	0,755	1,30769836	4	6,3	0,681909085	3
359	TNNKGSAAWMA	MAP3K7	TAK1/MAP3K7	S192	7,241666667	1,330907168	6	7,784	0,42349026	5
360	SDTTTTFCGTP	Serum/glucocorticoid regulated kinase 3	3-Phosphoinositide_dependent_protein_kinase_1	T320	3,495	3,495	2	3,005	1,757903581	4
361	RERGESPTTPP	MLK3 / MAP3K11	AKT1	S674	2,455	2,465405646	4			0
362	YFRYLSEVASG	14-3-3 Beta	PKCzeta	S131	7,816666667	0,677191914	6	7,823333333	0,600878986	6

363	WRRKSSDRKGG	HLA-A	Protein_kinase,_cAMP_dependent,_regulatory,_type_I,_alpha	S337	8,53	0,322645316	6	8,438333333	0,358395343	6
364	TEDQYSLVEDD	Phosphatidylinositol 3 kinase, regulatory subunit, alpha	Phosphoinositide-3-kinase,_catalytic_subunit,_gamma	S608	9,051666667	0,472314043	6	8,8	0,303644529	6
365	RGRGSSVGGGS	ASK1	AKT1	S83	7,918	0,576936738	5	7,218	0,630916793	5
366	MGQAGSTISNS	Connexin 43	CK1delta	S325	2,28	3,376033175	5	1,2475	2,160733382	4
367	RLRPLSYPQTV	Ras related C3 botulinum toxin substrate 1	AKT1	S71	8,673333333	0,136096371	6	8,301666667	0,501112651	6
368	GQLRGSATRAL	Casein kinase 1, epsilon	CK1epsilon	S323	8,513333333	0,405777717	6	8,056666667	0,398692307	6
369	RKRKNSRVTFS	NIPP1	PKA	S199	7,746666667	0,233071281	6	8,065	0,316899038	6
370	IKRQLSMTLRG	PCTAIRE protein kinase 1	PKA	S12	7,53	0,345012077	6	7,886	0,497899588	5
371	LMKSDSYPRFI	Regulator of G protein signaling 7	PKCalpha	S434	8,598333333	0,419381158	6	8,736666667	0,313138237	6
372	GHSNSSPRHSE	Heterogeneous nuclear ribonucleoprotein D	GSK3beta	S83	6,432	1,004497885	5	6,252	2,156166969	5
373	RARSTSLNERP	Tuberin	AKT1	S939	8,93	0,279284801	6	8,6	0,134350288	4
374	DAPPLSPFPHI	Retinoblastoma like 1	CDK4	S964	9,785	0,186971477	6	11,295	0,418917255	6
375	DYDDMSPRRGP	Heterogeneous nuclear ribonucleoprotein K	JNK3 / MAPK10	S284	7,036666667	0,448540844	6	6,908	0,328475265	5
376	GGARASPATQP	Sam68	ERK	S58	2,158	2,711135555	5	0,983333333	1,390643336	3
377	NSRRPSRAMWL	Vitronectin	PKA	S397	7,846	0,164754363	5	7,785	0,092330927	4
378	QERRGSNVALM	Protein tyrosine phosphatase nonreceptor 7	PKA	S83	9,531666667	0,372532623	6	9,526666667	0,192498196	6
379	VHRDLSRDRPL	Kell blood group protein	PKC	S63	8,26	0,195703858	6	7,933333333	0,709921748	6
380	LEVSDSESDEA	SPIB transcription factor	CK2	S146	8,174	0,211149236	5	8,08	0,504579032	5
381	LRRRLSDSSFI	Synapsin II	PKA	S10	9,421666667	0,334535333	6	8,7	0,166232769	6
382	RTRRISQTSQV	Ryanodine receptor 2	PKA	S2808	8,883333333	0,241499942	6	8,334	0,187147001	5
383	APKAPSKKEKK	DAB2	PKC	S24	6,82	0,816374097	3	6,92	0,731334397	4
384	KVEPASPPYYS	Peroxisome proliferator activated receptor, gamma	ERK2_JNK1	S112	9,455	0,19636276	6	9,361666667	0,305364081	6
385	HVDNEYSQPPR	CD5	CK2	S483	4,014	2,464196421	5	5,45	1,827147139	3
386	ETESPYQELQG	DAP12	SYK	Y91	5,316666667	0,793361344	3	2,885	2,885	2
387	HVEDLYVEGLP	TFII-I	Bruton's_tyrosine_kinase	Y398	7,115	0,475	2	6,786666667	0,210607587	3
388	RNDQVYQPLRD	CD3 Delta	nd	Y149	1,6475	1,157829327	4	3,61	2,16	2
389	ATLDVYNPFET	SCAMP3	EGFR	Y41	6,476666667	0,471475226	3	7,04	0,17	2
390	NegCtrl	Mixed peptides without STY	na							
391	SptCtrl	Used for production/QC	na		9,508333333	0,785099074	6	8,95	0,471769718	6
392	PosCtrl	Mixed kinase substrate peptides	na		1,5775	2,387806263	4	0,7975	1,381310519	4
393	DEELHYASLNF	CD33	Src_kinase	Y340	7,95	0,454972527	5	7,792	0,339611543	5
394	GKKATQASQEY	H2AX histone	ATM / nd	S140	5,696666667	0,259015229	3	6,23	0,58	2
395	IENPQYFSDAC	TRKA	TRKA couples to Shc	Y490	3,11		1			0
396	QGVDTYVEMRP	Colony stimulating factor 1 receptor	c-Fms couples to Shc / Colony_stimulating_factor_1_receptor	Y809	2,665	2,665	2	5,39		1
397	AATMKTFCGTP	AKT3	PKCzeta	T305	0		1	5,395	0,695	2
398	DNGGFYISPRI	Lck	SYK	Y192	3,544	2,133772247	5	5,22	0,55	2
399	KTNLSYYEYDK	Bone marrow kinase BMX	FAK	Y40	8,254	0,12563439	5	8,564	0,27861084	5
400	VSNFNYFYHRE	Potassium voltage gated channel subfamily A member 2	nd	Y415	7,578	0,259028956	5	7,27	0,531068106	6
401	LEPLCTPVVTC	c-Fos	RSK;PKA	S362	4,535	2,736864081	4			0
402	EEIYLTPVQRP	MAPK8 interacting protein 1	JNK1	T284	6,34		1	6,5	0,01	2
403	PVRTYTHEVVT	Cyclin dependent kinase 2	cdk7 / nd	T160	2,323333333	1,818503658	3	5,26		1
404	EEGEGYEEPDS	CD19	ABL	Y508	7,0725	0,235199384	4	6,648	0,459451847	5
405	QPGHPTPPPTP	C/EBP alpha	GSK3	T229	5,96	0,74	2	6,213333333	0,536987482	3
406	ALDFRTPRNAK	Opioid receptor mu 1	Beta-adrenergic_receptor_kinase_2	T182	7,018	0,19436049	5	6,832	0,890626746	5
407	PEETQTQDQPM	HSP 90A	dsDNA-activated_protein_kinase	T7	5,638	0,857633955	5	5,46	0,39	2
408	EGKHLYTLDGG	RACK1	Src	Y228	6,2		1	5,51		1
409	WPQPLSTLPTS	OCT binding factor 1	nd	S184	5,015	0,515	2			0
410	EFRRLSISAES	Phosphorylase kinase, muscle, alpha-1 subunit	Phosphorylase_kinase,_muscle,_alpha-1_subunit	S1018	7,01		1	6,57		1
411	PAAASSEDIER	Galanin	nd	S117	5,34	0,968194195	3	6,54	0,623912387	3
412	DHIEVSDDEDE	Hsp90 co-chaperone Cdc37	CK2	S13	6,9875	0,543110256	4	6,828333333	0,420452798	6
413	PEKPKTPQQLW	Upstream binding factor	ERK1/2	T201	6,7	0,682104586	3	6,566	0,29608107	5
414	GAGGYTQSPGG	RPA2	DNA-dependent_protein_kinase_catalytic_subunit	T21	0	0	2	3,25	2,561262709	3

415	GPHRSTPESRA	Presenilin 1	CDK5	T354	2,64	2,31	2			0
416	KVVALYDYMPM	Bruton's tyrosine kinase	BTK;ABL	Y223			0	5,37		1
417	EDTTSTFCGTP	Serum/glucocorticoid regulated kinase 2	3-Phosphoinositide_dependent_protein_kinase_1	T253	6,776666667	0,131993266	3	6,0475	1,19572938	4
418	GKNRPSSGSLI	Quinoid dihydropteridine reductase	CAMKII	S223	7,426	0,516782353	5	6,978	1,123804253	5
419	MSETVPPAPAA	Histone 1, H1a	CDC2	T4	2,754	1,743669693	5	2,24	2,24	2
420	APRQSSPSKSS	DAB1	CDK5	S491	7,556	0,438889508	5	6,973333333	0,430993684	3
421	DVDQGSLCTSF	IKK alpha	NIK	S176	6,293333333	0,34422215	3	4,9	1,266353031	4
422	TKALQSPKRPR	TFII-I	EphB2	S668	8,536666667	0,176225865	6	7,976666667	0,658702428	6
423	PTQPTSASPSL	Carcinoembryonic antigen-related cell adhesion molecule 1	c-Src	Y493	1,444	1,775033521	5			0
424	AAPASSSDPAA	EIF3S5	CDK11	S46	0		1	0		1
425	GSDSSSESEPE	MAX protein	CK2	S142	7,996	0,341736741	5	7,676666667	0,486335504	6
426	AKIQASFRGHM	Neurogranin	PKCalpha;PKM;PKCgamma;PKCbeta-1	S36	9,325	0,283299018	6	9,095	0,469387899	6
427	SSTPLSPTRIT	Lamin A/C	CDC2-kinase	S22	7,57	0,267656496	5	7,006	0,688958634	5
428	ETPAISPSKRA	dUTP pyrophosphatase	CDC2	S99	6,898	0,938901486	5	6,1075	1,015907845	4
429	PQSPGSPLEEE	p47-phox	CK2	S348	7,084	0,673634916	5	7,564	0,600353229	5
430	GSRRGSFDATG	Desmoplakin	PKA	S2849	6,36	0,09	2	6,66	0,375566239	4
431	LVRSASSDTSE	Solute carrier family 9, isoform A3, regulatory factor 1	GRK6A	S290	6,97	0,06	2	6,416666667	0,849208782	3
432	NETMLSPREKI	Retinoblastoma like 2	CDK	S1068	5,734	0,92607991	5	6,6425	0,280568619	4
433	SDSRKSMRQST	TCFL1	PKC	S132	6,6475	1,335035112	4	7,176666667	0,762029454	3
434	EAKRSSADKGV	Brain sodium channel 2	PKA	S525	3,825	2,268705578	4	5,842	0,496362771	5
435	PSAYGSVKAYT	Annexin II	PKC	S25	8,411666667	0,314134614	6	7,953333333	0,441990447	6
436	NLLPMSPEEFD	STAT1	MAPkinase;KIT	S727	9,156666667	0,410960934	6	8,983333333	0,287556758	6
437	KVGVSSRINEW	CALDESMON 1	CDC2_Kinase	S724	7,231666667	0,881048933	6	7,502	0,568204189	5
438	PVRCYSAEVVT	Cyclin dependent kinase 5	CK1	S159	6,483333333	1,481256974	6	6,34	0,775983247	4
439	SERRGSHPYID	Phosphodiesterase 7A cAMP-specific high-affinity	Protein_kinase	S58	7,834	0,27861084	5	7,734	0,165481117	5
440	GGYTQSPGGFG	RPA2	CDC2	S23	6,553333333	0,102740233	3	6,91	0,286705424	5
441	SWKENSPLNVS	Coagulation Factor III	PKC	S285	7,546	0,450670611	5	7,45	0,64136573	4
442	GSPRVSVTDDS	Nuclear Factor Of Activated T Cells,Calcineurin-Dependent 1	DYRK / PKA	S294	5,625	0,835	2	6,82		1
443	RSTPESRAAVQ	Presenilin 1	GSK3beta	S357	3,68	2,633983042	3	3,803333333	2,690728939	3
444	SVRMLSGSKEK	Opioid receptor mu 1	CAMKII	S268	8,973333333	0,534529908	6	8,038	0,231637648	5
445	EKARLSYSDKN	Protein kinase C delta	PKCdelta	S645	7,735	0,687671191	6	7,59	0,574920284	6
446	EKARLSYSDKN	LKB1	PKA/ p90RSK;MSK1;S6K1;PKA;LKB1	S424	8,873333333	0,337424889	6	8,095	0,563404828	6
447	NRSFLSLKHTP	Notch2	GSK3beta / nd	S2070	6,232	0,369507781	5	6,654	0,514066144	5
448	LVSPDSPRSID	Leukemia inhibitory factor receptor, alpha	MAPkinase	S1044	7,056	0,443513247	5	7,636	0,439390487	5
449	KAVDGYVKPQI	STAT5A	JAK2	Y694	2,91	2,064477335	3	0,236666667	0,33469721	3
450	IEDNEYTARQG	c-Src	c-Src	Y419	4,88	1,331029677	5	2,2375	2,622693034	4
451	SNDKVYENVTG	FAK	integrin etc / GRB2	Y925	3,482	2,106545988	5	2	2,828427125	3
452	TEENIYQVPTS	DAB1	c-Src	Y220	3,574	1,951087902	5	4,92	0,423398945	3
453	ATKSGSTTKNR	Eukaryotic translation initiation factor 4E	Mnk1;PKC	S209	0,69		1	1,963333333	1,81819202	6
454	NegCtrl	Mixed peptides without STY	na							
455	SptCtrl	Used for production/QC	na		1,15		1	3,39	3,39	2
456	PosCtrl	Mixed kinase substrate peptides	na		3,04	3,04	2	5,25		1
457	LNEEWYVSYIT	SLP 76	Lck	Y423	7,216666667	0,298421775	6	6,728	0,703147211	5
458	RRCKHYVELLV	Vanilloid receptor like channel 2	Lyn	Y253	8,518333333	0,165067326	6	8,303333333	0,241982552	6
459	LSNPAYRLLLA	Discoidin domain receptor	Discoidin_domain_receptor	Y513	7,69	0,355058681	3	7,6675	2,187457142	4
460	RGQEVYVKKTM	TEK receptor tyrosine kinase	autophosphorylation / nd	Y992	9,818	0,302483057	5	9,306666667	0,23127665	6
461	DGENIYIRHSS	Erythrocyte membrane protein band 41	EGFR	Y660	6,45	0,435660418	4	5,906666667	1,192765228	3
462	QESEDYSQPST	Oncoprotein Mdm2	c-abl_kinase	Y394	2,11	2,11	2	4,68		1
463	WGCGNSLRTAL	Lamin A/C	PKCalpha	S525	1,68	2,375878785	3			0
464	IDPFTYEDPNE	EphB3	EphB3	Y614	6,825	0,360173569	4	6,55	0,991261822	4
465	GSVEQTPKKPG	Cyclin-dependent kinase inhibitor 1B	cyclin_E-CDK2	T187	4,33	0,99	2	4,8475	2,850520786	4
466	ICSVNTPREVT	Protein tyrosine phosphatase nonreceptor 7	Erk;ERK2;MAPK14	T105	5,46		1	0	0	2

467	RSQLETPETLL	Tumor necrosis factor receptor 2	nd	T436	4,513333333	1,116820885	3	3,416666667	2,393077981	3
468	LPVPEYINQSV	EGF receptor	EGFR	Y1092	7,122	0,264907531	5	6,735	0,552697928	4
469	RKRPATDDSST	Cyclin-dependent kinase inhibitor 1B	AKT1	T157	1,623333333	2,295740016	3	0	0	2
470	TRSKGTLRYMS	PKR	PKR	T451	8,368333333	0,16045941	6	7,743333333	0,516225618	6
471	IDEPSTPYHSM	Protein phosphatase inhibitor 2	GSK3beta;NCLK	T73	5,423333333	0,299369708	3	3,975	2,205	2
472	NELKKKASLF	Dematin	? / PKA	S403	8,79	0,372066302	6	8,114	0,841893105	5
473	GDDEASATVSK	Rhodopsin	RK	S334	6,64	0,590355825	5	7,023333333	0,809334432	3
474	LQGFNSPGMLS	Myocyte specific enhancer factor 2A	ERK5	S355	6,31	0,165126214	3	6,722	0,253092868	5
475	AKKEESEESDD	Ribosomal phosphoprotein large P1	nd	S101	7,335	0,223215143	4	7,366	0,503134177	5
476	GTNPGTPPAST	SRC1	ERK2	T1179	0,915	0,915	2	5,02		1
477	KEEPQTVPEMP	c-Jun	CK2;DNA-dependent_protein_kinase_catalytic_subunit	T231	5,316666667	0,555117605	3	5,385	0,285	2
478	QLLCSTPNGLD	CDC25C	Chk1, Chk2 , Cytokine_inducible_kinase_Cell_cycle_checkpoint_kinase;CHK2;MAPK14;MARK3	S216	5,88	0,87	2	5,673333333	1,25659151	3
479	GSKCSTWPLPQ	Prolactin receptor	nd	T415	6,98		1	4,17	0,67	2
480	PRSTHTAYIK	ADAM 12	c-Src	Y907	9,8	0,468045582	6	9,235	0,222167354	6
481	PQPPKSPGPHS	RAD9	nd	S336	4,116	2,254458693	5	5,763333333	0,199053315	3
482	KRRGASDLSSE	Spermidine/spermine N	CK2	S146	0		1	1,945	1,945	2
483	QGSDVSLTACK	HLA-A	PKC	S359	4,874	2,459159206	5	5,605	0,875	2
484	YPFALSKSSMY	Kinetochore associated 2	Never_in_mitosis_gene_A-related_kinase_2	S165	6,02	0,23	2	5,853333333	0,596899396	3
485	KSEPSSPDHGS	Phospholipase C, beta 1	ERK1	S982	6,225	0,285	2	6,023333333	0,485958389	3
486	LGRTQSAPLPQ	Histone deacetylase 4	CaMKIV	S467	0		1			0
487	RVWYVSNIDGT	Phosphohexose isomerase	CK2	S185	5,16	0,39	2	3,795	0,145	2
488	TTCVDTRWRYM	HIR	Protein_kinase_C_delta	T53	6,8475	0,755194511	4	4,953333333	1,8694087	3
489	PSGLWSPAYAS	NUP210	CyclinB;p34cdc2	S1881	9,408333333	0,249694257	6	9,118333333	0,458999516	6
490	YTRVQSMALPP	Forkhead box protein L2	PAK1	S263	6,488	0,338904116	5	6,46	1,025012195	4
491	RKAKRSLAPRF	PKR	PKR	S242	8,568333333	0,458236353	6	8,29	1,276698868	5
492	SLLRLSLYNNC	MHC class II transactivator	Protein_kinase,_cAMP-dependent,_catalytic,_alpha	S1050	4,328	2,302515147	5	5,986666667	0,48230926	3
493	SASQLSAEEEE	HOX B6	CK2	S214	7,8425	0,335289054	4	7,298	0,772978654	5
494	ILRKVSGHPNI	Phosphorylase kinase muscle gamma-1	Phosphorylase_kinase_muscle_gamma-1	S82	6,745	0,055	2	7,343333333	0,281937739	3
495	VLNAFSQAPST	Serum response factor	DNA-dependent_protein_kinase_catalytic_subunit	S435	6,09	0,352798337	3	6,395	0,295	2
496	NNEVGSMKIQS	Crystallin, beta A3	nd	S160	7,5925	1,494813952	4	6,543333333	0,210765799	3
497	LERQLSLEQEV	Arachidonate 5 lipoxygenase	MAPKAPK2 / MAPK2	S271	8,493333333	0,32749894	6	7,868	0,531315349	5
498	LIPQQSINEAI	Glutamate receptor ionotropic, AMPA 1	PKC;CAMKII	S849	7,528333333	0,693912018	6	7,4675	0,561087114	4
499	RDKEVSDDEAE	HSP 90A	CK2alpha_1	S231	7,84	0,368465286	6	6,9925	1,073274778	4
500	GARRSSWRVIS	14-3-3-Eta	SDK1 is proteolytic fragment of PKC delta	S59;S60	9,636666667	0,164485731	6	8,921666667	0,446781702	6
501	VSSAASVYAGA	Keratin 18	p34cdc2	S33	7,28	0,638592202	5	6,77	1,220013661	6
502	LQRYSSDPTGA	EGF receptor	Calcium/Calmodulin_dependent_protein_kinase_II_gamma	S1071	6,005	0,856081188	4	6,225	0,492163591	4
503	QAKVGSLDNVG	Microtubule associated protein 4	MARK	S1073	4,636666667	0,773491363	3	6,53	0,68	2
504	KTKFASDDEHD	Autoantigen La	nd	S366	8,645	0,376552343	6	8,0625	0,439623418	4
505	PLVQRGSANGL	Beta-adrenergic receptor kinase1	PKA	S685	6,823333333	0,720940743	3	7,2875	0,728023866	4
506	RPRVTSGGVSE	Cell cycle checkpoint kinase I	ATR	S280	6,855	0,604586636	4	7,03	0,466958242	4
507	LYRSPSMPENL	CDC25C	cdc2-cyclin_B_kinase	T48	6,195	0,485	2	6,156	0,779297119	5
508	NIVLLSAEEKK	CD20	CK2	S231	8,573333333	0,704099109	6	8,19	0,456990153	5
509	ETKGKSFEEIA	Solute carrier family,member 2	PKA	S491	9,261666667	0,245249306	6	8,615	0,512404463	6
510	GTLRTSISVER	Bradykinin receptor 2	PKC	S373	8,705	0,269675731	6	7,806	0,660502839	5
511	LEKRASGQAFE	Stathmin 1	PKA	S16	7,074	0,536492311	5	5,95	1,14166545	3
512	PIDMESQERIK	c-Jun	c-Abl;JNK	Y170	7,17	1,158166655	4	7,63		1
513	VAYHPYPEDYG	IRS 2	Insulin_receptor	y629	8,321666667	0,213183541	6	8,345	0,581857657	6
514	AEGSAYEEVPT	Phospholipase C, gamma1	FGFR1	Y472	4,9	2,877611857	4	6,48	0,415331193	4
515	KEVSKYSDIQR	PDGF receptor, alpha	PDGFRalpha to PI3K	Y754	5,605	0,835	2	7,01	0,567230112	4
516	EEPSIYESVRV	DAPP1	Lck;PI3-kinase	Y139	7,016666667	0,371513421	3	6,535	0,245	2
517	LGQRIYQYIQS	DYRK1B	DYRK1B	Y271	7,97	0,744222189	3	7,523333333	0,30619529	3
518	NegCtrl	Mixed peptides without STY	na							

519	SptCtrl	Used for production/QC	na		7,328333333	0,321217303	6	8,325	0,24074537	6
520	PosCtrl	Mixed kinase substrate peptides	na		3,245	3,245	2	1,163333333	1,645201778	3
521	PDEILYVNMDE	AXL receptor tyrosine kinase	auto	Y821	7,42	1,066239498	3	6,61	0,59	2
522	NVVPLYDLLLE	Estrogen receptor, alpha	c-Src;Lck	SY537	9,363333333	0,60895174	6	9,433333333	0,221860817	6
523	SDDVRYVNAFK	VEGF receptor 1	VEGF_receptor_1	Y1213	7,708333333	0,501179165	6	8,26	0,447034674	5
524	EPAHAYAQPQT	CrkL	BCR	Y207	6,77	0,671912693	3	0		1
525	SSLKAYGNGYS	Beta-2-adrenergic receptor	Insulin_receptor	Y350	5,19	0,03	2	6,7225	0,420260336	4
526	NKPTVYGVSPN	ABL	nd	Y245	1,785	1,785	2	2,842	2,44157654	5
527	RNPGFYVEANP	Phospholipase C, gamma1	PKA;PKC	Y783	6,376666667	0,181169043	3	7,485	0,415301096	4
528	FMMTPYVVTRY	JNK1	MAP2K4	Y223	9,214	0,401427453	5	9,088333333	0,25215185	6
529	SAWPGTLRSGM	HSP22	ERK1	T87	4,08	2,452305038	4	2,376666667	3,361114233	3
530	PIRVYTHEVVT	CDC2	Tita: PK161 WEE_1_tyrosine_kinase	Y15	0	0	2	4,726	2,659139711	5
531	SSPPGTPSPAD	CCAAT/Enhancer binding protein, beta	CaMKII	S325	6,89		1	7,008	0,924497701	5
532	GSVLSTACGTP	CAM kinase 1	Calcium-calmodulin_dependent_protein_kinase_I_kinase	T177	5,76		1			0
533	RRRRPTPAMLF	Protein phosphatase 1, regulatory subunit 1B	PKA	T34	7,165	0,462466215	4	7,255	0,245	2
534	FESIESYDSCD	C ets 1 protein	CaMK2	S257	7,393333333	0,2153808	6	7,802	0,63857341	5
535	EPLPVTPTRDV	PAK1	CDK5	T212	0	0	3	2,545	2,545	2
536	GKLFYTFCGTI	PAS kinase	PAS_kinase	T1161	8,1	0,233380948	6	8,215	0,313089444	6
537	IHMVYSKRSGK	Small nuclear ribonucleoprotein 70 kD	nd	S137	9,955	0,430996906	6	10,27166667	0,440280845	6
538	DSQPESQVLED	Tumor protein p53 binding protein 1	ATM	S29	5,866666667	0,625157758	3	6,993333333	0,103387083	3
539	CEEEFSDSEEE	Histone deacetylase 1	CK2	S421	7,463333333	0,349555273	6	8,0725	0,480019531	4
540	KAARKSAPSTG	H3 histone, family 3A	nd	S10	5,895	0,475	2	6,91	0,71	2
541	QQREKTRWLNS	Adducin 1	Rho-kinase	T445	5,665	0,735	2	6,845	0,105	2
542	PTAAGTPNKET	CALDESMON 1	CDC2_Kinase	S759	2,935	2,935	2	0		1
543	RDKYKTLRQIR	Moesin	Rho-Kinase	T564	7,22	0,828299463	5	8,04	0,366878727	5
544	NSIAKTYVGTN	MAP2K5	nd	T315	3,2	2,264862027	3	3,9525	2,681057394	4
545	KETNESPWRSD	Glucocorticoid receptor	nd	S211	7,0725	0,929041845	4	6,8425	1,105540026	4
546	LARETSVDPDM	Sepiapterin reductase	Ca2+/calmodulin-dependent_protein_kinase_II	S213	6,9625	0,898703928	4	6,543333333	0,776759651	3
547	CDERTSLMSAE	Presenilin 2	CK1;CK2	S19	5,53		1	5,38		1
548	IAVRKSRDKAK	CCAAT/Enhancer binding protein, beta	ERK2	T235	9,186	0,48384295	5	8,66	0,364142829	4
549	DDRHDSGLDSM	I-Kappa-B-alpha	nd	S32	4,26		1			0
550	RLLDSSQIVII	E2F transcription factor 1	ATM;ATR	S31	6,156666667	1,049962962	3	7,2275	0,704818239	4
551	SIYTRSVIDPV	PAK2	PAK2	S197	7,015	0,996155108	4	6,79	0,32	2
552	ASGYISSLEYP	Complement component 1, subcomponent r	CK2	S206	8,858333333	0,311684491	6	9,286666667	0,307119231	6
553	VEPPLSQETFS	p53	DNA-dependent_protein_kinase_catalytic_subunit_Campt_208900_ATR	S15	7,946666667	0,346538438	3	7,962	0,406664481	5
554	LKRSHSDSLDH	CDC 25A	CDC2;PIM1	S115	7,013333333	0,151070256	3	7,305	0,744686735	6
555	ATTGVSQETSE	Glucocorticoid receptor	DNA-dependent_protein_kinase_catalytic_subunit	S508	3,648333333	2,596083054	6	6,073333333	0,322937903	3
556	LMDNAYFCEAD	Growth hormone receptor	nd	Y534	7,343333333	0,180431581	6	7,826	0,325367485	5
557	KRRQTSMTDFY	Cyclin-dependent kinase inhibitor 1A	AKT1	S146	7,782	0,568380154	5	7,386	0,313853469	5
558	GGADDSAEEGD	Heterogeneous nuclear ribonucleoprotein C	CK2	S260	8,206666667	0,081377037	6	7,828333333	0,407979438	6
559	LKRSLSEMEIG	Serum response factor	Potassium_voltage_gated_channel_subfamily_A_member_2;pp90rsk;CaMKII__	S103	7,113333333	0,615241597	6	7,85	0,456333212	5
560	PGRPLSSYGMD	MEK1	PAK1	S298	6,936	0,746527963	5	6,833333333	1,269575607	3
561	LSYLQSPITTS	Dual specificity phosphatase 1	ERK2	S359	7,9775	0,386417844	4	7,853333333	0,351220096	3
562	INRSASEPSLH	RAF1	PKA;RAF1;AMPK	S621	6,36	0,591819229	4	6,35	0,50039984	3
563	DFEGFSYVNPQ	Protein kinase C alpha	PKCalpha	S657	9,796666667	0,609061208	6	9,866	0,360643869	5
564	GDRTSTFCGTP	PKN	3-Phosphoinositide_dependent_protein_kinase_1	T774	1,195	1,195	2	2,66	2,66	2
565	GVERSSPSKCP	BRCA1	CDK2	S1497			0	7,37	0,465403051	3
566	DVHMVSDSDGD	FAS associated factor 1	CK2beta	S289	6,954	0,263256529	5	7,335	0,215	2
567	ALTEDSTQTSD	Formyl peptide receptor 1	Beta-adrenergic_receptor_kinase1	S328	4,6225	2,595490079	4	6,565	0,725	2
568	RRRMASMQRTG	E1A binding protein p300	AKT;p70S6_kinase;pp90Rsk	S1834	7,336666667	0,306521705	6	7,44	0,750919436	5
569	SRKRLSQDAYR	p47-phox	PKCalpha;PKCbetaII;PKCdelta	S320	9,125	0,273358739	6	9,293333333	0,315893794	6
570	GEKLHSDSGIS	Nerve growth factor receptor	PKA	S303	6,943333333	0,3044485	3	7,036	0,396716523	5

571	EHRKSSKPIME	Transcription factor HES-1	PKC	S38	5,235	0,765	2	4,97	1,047067333	4
572	GEFLRTSCGSP	AMPK alpha 2	AMPKK	T174	6,95	0,01	2	3,66	2,618752884	3
573	PTPPLSPSRRS	c-Myc	CK2;GSK	T58	9,05	0,212524508	6	8,96	0,256748905	5
574	QLRRPSDRELS	NFKB3	PKA	S263	6,1025	0,841200779	4	7,47		1
575	LTNRHSLPFSL	CBL	INSR	Y371	7,796	0,347136861	5	8,078	0,506770165	5
576	VEDNRSQVETD	AQP4	CK2	S285	6,5525	0,640912436	4	7,46	0,125698051	3
577	DDEDCYGNYDN	3-Phosphoinositide dependent protein kinase 1	c-Src	Y373	6,2475	1,079615093	4	7,331666667	0,875336443	6
578	DVLKFYDSNTV	PAK2	Lck, Fyn, Hck / Src	Y130	6,96	0,408493166	3	6,945	0,846241691	4
579	EADGVYAASGG	FES tyrosine kinase	FES_tyrosine_kinase	Y713	0	0	2	6,57	0,71	2
580	FLFNMYLTRER	Homeo box A10	nd	Y343	0	0	2	4,4475	0,429789193	4
581	RPDHIYDEPEG	DOK2	Abl / nd	Y345	4,4275	2,283094118	4	3,206	2,647728083	5
582	NegCtrl	Mixed peptides without STY	na		6,931666667	0,832934905	6	6,746	0,786907873	5
583	SptCtrl	Used for production/QC	na		0		1	2,895	2,245	2
584	PosCtrl	Mixed kinase substrate peptides	na				0	5,56		1
585	EDENLYEGLNL	CD79A	nd	Y188	7,228333333	0,473019262	6	7,924	0,347942524	5
586	RESSVYDISEH	NR2B	CaMKII	S383	7,5025	0,672880933	4	8,2975	0,279676152	4
587	CERRFSRSDQL	WT1	PKA	S365	5,04	0,14	2	6,51		1
588	TDEDIYLLGKA	Sialyltransferase 1	nd	Y391	7,17	0,292438028	5	6,956666667	0,803713603	3
589	ASKRSYQFWDT	N-Myristoyl transferase 1	Lyn	Y117	8,284	0,384686886	5	7,594	0,532413373	5
590	EVERTYLKTKS	Glutamate receptor ionotropic, N-methyl D-aspartate subunit 2A	Src	Y1105	9,951666667	0,354608548	6	9,32	0,390170903	6
591	TGSVDYLALDF	GAB2	ZAP70	Y614	7,61	0,17907168	3	7,42	0,297097066	6
592	YKVILYELENF	Crystallin beta B3	nd	Y29	6,663333333	0,430219582	3	6,2875	1,362761443	4
593	VDAAVTPEERH	Amyloid beta A4 protein	GSK3beta	T743	4,0475	1,939527971	4	6,62	2,98	2
594	KDDKLTPKIGF	Ezrin	CDK5	T234	6,841666667	0,438650076	6	8,05	0,382709638	3
595	DNTPHTPTPFK	B-Myb	CDK2	T518	5,91	0,438482231	3	6,886	0,790913396	5
596	PDHQYYNDFPG	Shc	SYK;c-Src	Y350	5,49	0,43481797	3	4,963333333	2,200065656	3
597	IVADQTPTPTR	Activating transcription factor 2	JNK2;MAPK14	T51	0,85	1,7	5	2,22	2,269988987	4
598	GETRFTDTRKD	Eukaryotic translation elongation factor 2	eEF2_kinase	T57			0	3,03	3,03	2
599	LHRDKTPLHQK	B-Myb	CDK2	T494	4,825	0,405	2	2,84	2,84	2
600	TTSQLYDAVPI	3-Phosphoinositide dependent protein kinase 1	c-Src	Y9	6,526666667	0,207578633	3	6,703333333	0,322937903	3
601	LPPGASPQRSR	NFAT1	nd	S268	7,04	2,6468094	3	7,416	0,606089102	5
602	DDGEFSDDSGA	eIF-2B epsilon	CK1	S466	6,226666667	0,671830005	3	7,64	0,336080348	4
603	HKGHLSEGLVT	MAPK6	MAPK6	Y189	7,17	0,954840301	5	7,93	0,308274769	6
604	QEQESSGEEDS	Protein phosphatase inhibitor 2	CK2	S121	6,695	0,418897362	4	6,92	0,392513269	3
605	RLRPRTRKVKS	Neutrophil cytosolic factor 4 40kDa	PKC	T154	8,973333333	0,263037809	6	8,588333333	0,278891656	6
606	MEEGQTQKGCF	Glycoprotein M6A	PKCalpha	T10	4,73		1	4,97	0,853268227	3
607	QSKRSTMVGTP	PAK3	Pyruvate_dehydrogenase_kinase,_isoenzyme_1	T423	7,3525	0,742205329	4	7,315	0,288574081	4
608	QERRKYLKHRL	STAT2	JAK1	Y690	7,76	0,472821319	5	7,1025	0,328814157	4
609	RFIIGSVSEDN	Acetyl-CoA carboxylase alpha	cdpk	S23;S62	6,773333333	0,862567228	3	7,262	0,688139521	5
610	DPWGGSPAKPS	Epsin 1	CDC2	S357	3,25	3,25	2	6,85	0,536982309	4
611	LDSRLSPPAGL	STAT5A	IL-2	S780	2,54	2,54	2	1,536666667	2,173174841	3
612	MASGVTVNDE	Cofilin 2	LIMK1	S3	4,853333333	1,24756652	3	3,413333333	2,420266285	3
613	RGKKKSGCLVL	RhoA	PKA	S188	8,8	0,441663522	6	8,405	0,357246414	6
614	MASGVAVSDG	Cofilin 1	LIMK1;TESK1;TESK2;LIMK2	S3	5,703333333	0,718578844	3	4,62		1
615	YQQRNSPGVPT	Golgin 95	CDC2;PP2A	S25	4,43		1	1,066666667	1,508494467	3
616	DEDACSDTEAT	Protein phosphatase inhibitor 2	CK2	S87	5,9475	0,321743298	4	5,536666667	0,960185167	3
617	RAREASGAPTS	Androgen receptor	AKT1	S213	2,275	1,975	2	4,985	0,025	2
618	KLFSSSVSEGF	Kell blood group protein	CK2	S383	7,713333333	0,431225643	6	8,445	0,224925914	6
619	GLMQQQKSFR	SHP2	PKCalpha;PKCbeta1;PKCbeta2;PKCeta	S591	8,711666667	0,164966327	6	8,504	0,076052613	5
620	LSTEGSDQEKE	Peptidylglycine alpha amidating monooxygenase	P-CIP2	S947	7,576	0,137200583	5	8,083333333	0,16569718	6
621	RRRFSSLHFMV	Caspase 9	ERK1;ERK2;MEK1;MAP2K2	T125	7,01	0,6303967	3	7,148	0,476210038	5
622	PLTPESPNDPK	PPAR gamma coactivator-1	MAPK14	S266			0	4,236666667	1,418951099	3

623	RRRAISETEEN	Oncoprotein Mdm2	AKT1	S166	6,1		1			0
624	IIGEKSFRRSV	Protein kinase C, mu	PKCmu	S738	7,86	0,288530761	4	7,0925	0,503754653	4
625	ALRRESQGSLN	Regulator of G protein signaling 14	PKA	S260	4,104	2,28807867	5	0	0	2
626	FRRQLSEPCNS	ETS variant gene 1	Ribosomal_protein_S6_kinase_alpha_5;ERBB2;Ribosomal_S6_kinase_1;Potassium_voltage_gated_channel_subfamily_A_member_2;PKA	S191	6,68	1,17940663	5	7,92	0,285744641	4
627	RKKRISVKKKQ	Cytohesin 2	PKC	S392	11,215	0,606458298	6	12,02166667	0,29700823	6
628	ENNVLSPLPSQ	p53	GSK3beta;Tumor_endothelial_marker_8_DNA-dependent_protein_kinase_catalytic_subunit	S33	2,715	2,739785576	4	5,27	0,24	2
629	RERKSSSSSED	B-Raf	PKA	S428	6,07		1	6,563333333	0,438583579	3
630	HERYSSPTAGS	Retinoblastoma like 1	CDK4	S650	6,39	0,87044816	5	6,844	0,593619407	5
631	FWSSLSPVAPL	ELK3	JNK	S357	7,486666667	0,323556624	3	7,4375	0,554137844	4
632	RRGSDSSEDIY	Protein phosphatase 1, regulatory subunit 3A	Ribosomal_S6_kinase_1	S48	10,71666667	0,350412455	6	10,55	0,22353225	6
633	LSRRPSYRKIL	cAMP response element-binding protein 1	PKA;MAPK;CaMK;RSK2	S133	9,693333333	0,223507395	6	9,341666667	0,424437536	6
634	KKKKPSRLKGD	APC	PKA	S2054	9,563333333	0,473661858	6	8,916666667	0,38663793	6
635	LKPGSSHRKTK	Bruton's tyrosine kinase	PKCbeta	S180	9,771666667	0,355734764	6	9,456666667	0,395502493	6
636	PCLPQYPHING	Fibroblast growth factor receptor 2	Fibroblast_growth_factor_2	Y813	3,832	3,182598938	5	6,12	0,913272504	3
637	SQRRESFLYRS	cAMP-specific 3',5'-cyclic phosphodiesterase 4B	PKA	S133	6,62		1			0
638	KARKKSSCQLL	Ras related protein Rap 1B	PKA	S179	8,861666667	0,335082908	6	8,396	0,42715805	5
639	GLQMGSNRGAS	Transgelin	PKC	S181	5,934	0,715055243	5	5,985	1,008476574	4
640	RMVQLSPPALA	T cell acute lymphocytic leukemia 1	ERK1	S122	2,575	2,61205379	4	5,86	1	2
641	FEEDDYESPND	SLP 76	ZAP70_Protein_tyrosine_kinase_TXK	Y113	7,43	0,699857128	3	6,913333333	0,726651376	3
642	VSFNPYEPELA	SYK	SYK	Y323	2,61	2,61	2	5,86		1
643	KEVKRYQCTFE	Metal regulatory transcription factor 1	Tyrosine_kinase	Y140	5,32		1	5,16		1
644	KMAEAYSEIGM	T-cell antigen receptor, zeta	lck / nd	Y123	4,69	0,71	2	6,37		1
645	PGLDEYNPFSD	SCAMP1	EGFR	Y37	7,636666667	0,260810702	3	6,688	0,521244664	5
646	NegCtrl	Mixed peptides without STY	na							
647	SptCtrl	Used for production/QC	na		8,981666667	0,171212993	6	8,693333333	0,383304347	6
648	PosCtrl	Mixed kinase substrate peptides	na		1,493333333	2,111892253	3	5,3	1,692911693	4
649	ITEEDYQALRT	Clathrin, heavy polypeptide	Src_kinase	Y1477	5,242	2,637062002	5	6,3025	0,83714918	4
650	KDGWVYYANHT	WW Domain containing oxidoreductase	JNK1	Y33	8,42	0,128840987	6	7,93	0,400915619	6
651	APEDLYKDFLT	VEGF receptor 2	VEGF_receptor_2	Y996	7,256	0,499143266	5	7,301666667	0,393760361	6
652	SRLSAYPALEG	CD5	Lck;Fyn	Y487	2,2125	2,249548566	4	5,28		1
653	HAQDTYLVLDK	Erythropoietin receptor	JAK2	Y368	6,98	0,345639504	3	6,99	0,641612552	3
654	EDLSAYASISF	IRS 1	Phosphatidylinositol_3-kinase,_catalytic_subunit,_alpha	y1229	6,82	0,252269697	5	6,8675	0,301278526	4
655	NQLFLYDTHQN	Phospholipase C, gamma 2	Bruton's_tyrosine_kinase	Y1217	5,45		1	5,776666667	0,756585899	3
656	SADSGYIIPLP	PDGF receptor, alpha	PDGFRalpha	Y1018	5,32	0,33	2	6,445	0,115	2
657	VLRPETPRPVD	Caspase 9	AKT1	S196	0,695	0,695	2	1,29	1,29	2
658	DATGDTPGAED	MAPK8 interacting protein 1	JNK1	T103			0	6,12	0,861510302	5
659	IGEGTYGVVYK	CDC2	Tita: WEE/MYT PK161	T161	8,078333333	0,460702965	6	7,74	0,407144528	6
660	RNEGVYTAIAV	PSTPIP1	ABL	Y345	6,135	0,165	2	6,65	0,398580816	3
661	RTAPYTPNLPH	SMAD4	ERK2	T277	2,435	2,435	2	5,27		1
662	AVHPLTPLITY	Lymphoid enhancer-binding factor1	Nemo-like_kinase	T155	6,216666667	0,526392334	3	6,9725	0,149394612	4
663	NNAIETVSINN	Dopamine receptor D1	nd	T360	3,57	1,513737097	3	4,42	0,15	2
664	GVDGDYEDAEL	BCR	FES_kinase	Y246	8,075	0,082006097	4	7,946	0,452574856	5
665	EKKAYSFCGTV	Ribosomal S6 kinase 1	Pyruvate_dehydrogenase_kinase,_isoenzyme_1	S227	8,591666667	0,286264252	6	7,881666667	0,296277835	6
666	KGGKYSVKDKE	L1 cell adhesion molecule	p90rsk	S1152	7,345	0,215	4	7,15	0,495008417	6
667	TWRRGSTAGGC	Calpain, large polypeptide L1	nd	T80	6,855	0,677292404	4	5,49		1
668	CQRRHTLPASE	Aralkylamine N-acetyltransferase	PKA	T31	4,4125	2,903130853	4	3,775	2,183054053	4
669	LVEPLTPSGEA	EGF receptor	ERK1;ERK2	T693	6,345	0,605	2	6,446666667	1,039914526	6
670	APLLSTPKRER	Kinesin like 4	Cyclin_B2	T463	7,96	0,492950302	5	6,84	0,481144469	4
671	DYIPGTETHMA	NIK	Tpl2	T559	5,885	0,415	2	0		1
672	PEPGPYAQPSV	CRK	ABL;EGFR;IGF-I_receptor	Y221	4,555	0,185	2	1,423333333	2,012897304	3
673	QPRCTSLDSAL	MAP3K8	AKT1	S400	1,01	1,55	2	0		1
674	DIKSDSILLTS	p21-activated kinase 7	PAK5	S573	7,07	0,096263527	3	6,89	0,46070598	4

675	EVPRRSGLSAG	Methyl CpG binding domain protein 3	Aurora_kinase_A	S24	5,943333333	0,867192148	3			0
676	LTRIPSAKKYK	PEA15	PKC	S104	10,20333333	0,180061718	6	9,523333333	0,245877115	6
677	LKQGASPNVQD	Cyclin dependent kinase inhibitor 2D	nd	S66	5,895	0,475	2	2,405	2,598090645	4
678	KTPKDSPGIPP	Ribosomal protein S6 kinase alpha 3	EphB2	S369	4,68		1	0		1
679	HGSRHSLASTD	Low density lipoprotein receptor-related protein 1	PKA	S4520	3,125	3,125	2	4,57		1
680	SPYSLSPVSNK	HCDH1	CDK2	S151	7,442	0,541161713	5	7,345	0,101118742	4
681	RKRKPSTSDDS	DNA topoisomerase II alpha	CK2	S1469	5,993333333	0,962023331	3	4,8425	1,433167384	4
682	QNLMQSVKETV	Vinculin	PKCalpha	S1101	4,59	3,461184768	3	6,035	1,557473916	4
683	LLREASARDRQ	Vanilloid receptor 1	PKCepsilon	S801	2,5425	2,547473013	4			0
684	PSPLPSPTASP	Amphiphysin	CDK4;CDC3	S272	3,35	2,386475784	3	3,965	0,675	2
685	IRSSMSGLHLV	Acetyl-CoA carboxylase alpha	AMPK	S80	6,875	0,185	2	6,6225	0,632905009	4
686	ENTFPSPKAIP	Nude like protein	CDK5/p35	S231	3,516666667	2,257673335	3	3,395	3,395	2
687	GERKKTLCGTP	Polo like kinase	nd	T210	3,64	2,596613179	3	0		1
688	YKFPSSPLRIP	Retinoblastoma 1	CDK4	S795	9,306666667	0,346201611	6	8,59	0,365695684	6
689	RSNPPSRKGSG	Connexin 32	PKC;PKA	S233	8,393333333	0,096896279	6	7,506666667	0,396344407	6
690	AAKRASRIYNT	Regulator of G protein signaling 10	PKA	S176	9,038333333	0,220258686	6	8,445	0,317477033	6
691	PSSRASSRASS	Connexin 43	nd	S367	8,205	0,259855729	6	7,428333333	0,504625824	6
692	KQEVESPTDKS	c-Myb	CK2alpha_1	S11	2,5725	2,575823897	4	5,8575	0,436255372	4
693	RSRDPSLMVDF	Fanconi anemia, complementation group A	AKT1	S1149	5		1	4,36		1
694	LERGNSGLGFS	Synapse associated protein 97	CaMKII	S232	7,06	0,394144644	4	6,963333333	0,263860232	3
695	RPRNYSVGSRP	6-phosphofructo-2-kinase/fructose-2,6-biphosphatase 2	AKT	S483	9,218333333	0,13234005	6	8,583333333	0,36385284	6
696	TKWYRSPRLLL	MAPK4	MAPK4	S196	6,028	0,437419707	5	6,215	0,348532638	4
697	PAARASKKILL	Beta-adrenergic receptor kinase1	PKC	S29	8,561666667	0,243681258	6	8,126	0,565423735	5
698	LERNLSFEIKK	Protein tyrosine phosphatase, nonreceptor-type, 12	PKA;PKC	S435	8,131666667	0,385461484	6	7,943333333	0,208619803	6
699	LLSELSRRRIR	eIF2 alpha	PKR;GCN2;Eukaryotic_translation_initiation_factor_2-alpha_kinase_3;dsRNA-PK;CK2;HCR;DSL;PKC	S52	9,173333333	0,149740516	6	8,37	0,443959458	6
700	GPRLVSNHSLH	Filamin A	CAMKII	S2523	8,313333333	0,257466287	6	8,186666667	0,296123098	6
701	SSDDDSDSEEP	CD45	CK2alpha1;CK2alpha2	S973	7,202	0,992560326	5	7,404	0,327511832	5
702	ELEGISPDELK	Glia maturation factor beta	PKA	S53	8,968333333	0,37262209	6	8,13	0,564304882	5
703	SSTDRSPYEKV	Mucin 1 transmembrane	GSK3beta	S1227	8,67	0,198158186	6	8,648	0,244409492	5
704	SQSPHSPDSSQ	CHOP	CK2alpha_1	S14	3,45	1,910968341	3	5,59		1
705	LKSPAYRDLAA	Regulator of G protein signaling 16	EGFR	Y177	7,48		1	4,88		1
706	SPQPEYVNQPD	ErbB2	ERBB2	Y1139	5,445	0,025	2	4,965	1,355	2
707	EEEHVYSFPNK	Paxillin	FAK	Y118	0		1	6,053333333	0,173269219	3
708	INPYHYKRVES	SMAD5	nd	Y128	6,973333333	0,411204194	3	7,95	0,532916504	4
709	EQETFYEQPPL	HIP55	ZAP70	Y345	6,32		1	5,606	1,189480559	5
710	NegCtrl	Mixed peptides without STY	na							
711	SptCtrl	Used for production/QC	na		5,634	1,678661371	5	5,11	0,26	2
712	PosCtrl	Mixed kinase substrate peptides	na		7,12		1	5,37		1
713	ADDSYYTARSA	ZAP70	Lck	Y493	4,335	0,555	2			0
714	FSGGLYGGLPP	NIPP1	Lyn	Y264	5,77		1	5,33		1
715	RGDKGYVPSVF	STAT4	IL-12;MAP2K6;STAT4	Y693			0	5,253333333	0,958343478	3
716	YGHPVYIVQEM	Ephrin B1	nd	Y329	3,43	3,43	2	5,26	0,83150466	3
717	QQQEVYGMMPR	Spectrin	Csk	Y1176	5,376666667	0,677757741	3	6,365	0,685	2
718	PNDSVYANWML	Ret	Ret	Y1096	5,895	0,205	2			0
719	IENPQYFGITN	TrkB tyrosine kinase	TrkB	Y532	5,94		1	6,46	0,58	2
720	QPFVKYATLIS	Leptin receptor	nd	Y986	8,85	0,268402683	5	8,456666667	0,857431565	6
721	SEGLPTPTKMT	Retinoblastoma 1	CDK2-cyclin_A	T821	5,395	0,585	2	6,73	0,4	2
722	MVRTQTESSTP	3-Phosphoinositide dependent protein kinase 1	PDK1	T33	1,505	1,505	2	3,47	0,81	2
723	TGDTYTAHAGA	ABL	nd	Y412	3,1		1	2,735	2,735	2
724	LRLQDYEEKTK	Ezrin	EGFR	T353	7,881666667	0,35913863	6	7,461666667	0,669288594	6
725	RPRSCTWPLPR	FKHR	AKT1	T24	6,84	0,503467973	5	6,5175	1,323468454	4
726	PPTGATANRLR	Casein kinase 1, epsilon	CK1epsilon	T407	3,56	3,56	2	0,985	0,985	2

727	SSTSVTPDVSD	PTEN	CK2	T366	3,93		1	6,755	0,295	2
728	EEEADSCFGDD	Cell division cycle 34	CK2	S222	6,9475	0,628346043	4	7,375	1,152876836	4
729	KLVQASEELLR	Nucleoside diphosphate kinase 3	nd	S61	7,2075	0,412090706	4	7,78	0,423398945	6
730	FSEITSPSKRS	ORC1	CDK2	S273	7,7	0,409243204	5	7,66	0,195703858	6
731	SLESLYSACSM	Grb10	Fyn	Y61	7,308333333	0,622452586	6	6,9425	1,591514609	4
732	EFTSRTPKDSP	Ribosomal S6 kinase 1	ERK2	T359	5,814	0,672981426	5	5,005	1,534000761	6
733	RKMKDTDSEEE	Calmodulin 1	Insulin_receptor;EGFR;c-Src;PTK_III	Y100	6,35	0,255049015	4	6,625	0,425998826	4
734	ILSGGTPKCCL	CDC25C	cdc2-cyclin_B_kinase	S214			0	4,686666667	0,348361243	3
735	SLFDRTPTGEM	Myosin light chain 1, embryonic muscle/atrial isoform	nd	T66			0	3,335	3,335	2
736	FFVIEYVNGGD	Protein kinase C, iota type	c-Src	Y325	8,073333333	0,26656248	6	7,508333333	0,751984412	6
737	GYLVDSVAKTM	MAP2K3	nd	S223	8,335	0,164595464	6	7,996666667	0,391223153	6
738	QKIHISKKWGF	Ribosomal protein L10	RPL10	S168	3,365	2,123493584	4	5,068	0,758667252	5
739	KKGARSRRLFS	Guanine nucleotide binding protein, alpha 15 subunit	PKCalpha	S336	10,125	0,26316978	6	9,676666667	0,237323033	6
740	LLEDDSDEEED	Vesicle associated membrane protein 4	CK2alpha_1	S30	9,333333333	0,478841194	6	8,686	0,405541613	5
741	DEQFVSLYGTE	TBK1	nd	S172	8,388333333	0,885709069	6	7,58	1,138639539	6
742	KTTQMSAAGTY	MAP3K11	HPK1	S281	6,7225	0,599723895	4	7,445	0,697871765	4
743	LHDALSGSGNP	Galectin 3	CK1	S12	4,93	1,579113675	3			0
744	VPPSSTDRSPY	Mucin 1 transmembrane	PKCdelta	T1224	5,626666667	0,730540135	3	6,97	0,34	2
745	SATIVSPPPSS	E2F transcription factor 1	CDC2	S332	3,6475	2,279817701	4	2,29	2,369367004	4
746	RSKKNSLALSL	Estrogen receptor, alpha	PAK1	S305	6,422	0,768749634	5	6,036666667	1,295745174	3
747	HFPQFSYSASS	AKT1	AKT	S473	8,423333333	0,513571373	6	8,262	0,536671222	5
748	KSFTRSTVDTM	Complement component 5 receptor 1	nd	S338	8,082	0,065848311	5	7,7	0,80867175	4
749	HWQQQSYLDSG	Beta-catenin	CK2beta	S29	6,645	0,408523765	6	5,704	1,90561906	5
750	KDFSSSKRMNT	Syntaxin binding protein 1	PKC	S313	9,211666667	0,494011358	6	8,86	0,490475959	6
751	IKRLRSQVQVS	Hepatocyte nuclear factor 4-alpha	AMPK	S304	5,72	0,312889757	4	4,732	2,590748154	5
752	FFPFHSPSRLF	Crystallin, alpha B	nd	S19	3,94		1	5,37		1
753	MRRSVSEAALA	Lipase hormone sensitive	nd	S554	6,41	0,5	2			0
754	PGTRLSLARMP	Glial fibrillary acidic protein	CaMKII;PKC;Rho_kinase	S38	6,6	0,783677229	4	6,475	0,265	2
755	CMRNFSRSDHL	Early growth response protein 1	CK2	S301	4,4975	2,613153028	4	6,116666667	0,267872275	3
756	MRRQRSAPDLK	Kinesin family member 1C	CK2	S1092	7,42	2,126405418	4	6,5725	2,579334943	4
757	SVPTPSPLGPL	CK2, alpha 1	CK2alpha_1	Y182	3,006	2,848498552	5	7,45		1
758	AEVLPSPRGQR	DNA topoisomerase II alpha	Proline_directed_kinase	S1213	8,28	0,145739494	5	7,236	1,029671792	5
759	KSKIGSTDNIK	Microtubule associated protein 2	MARK	S1679	8,713333333	0,303131801	6	8,683333333	0,377830062	6
760	GSSYGSLMTAH	NPR-B	nd	S526	5,955	0,752512458	4	5,563333333	1,063087746	3
761	VRRRQSVELHS	Aquaporin 2	PKA	S256	7,313333333	0,525441613	6	7,185	0,285	2
762	KKKKGSLDSDN	Solute carrier family 4, sodium bicarbonate cotransporter, member 4	PKA	S982	6,953333333	0,25772509	3	7,5	0,126293309	4
763	TIDPASPQSPE	Solute carrier family 9, isoform A1	MAPK14	S723	6,406666667	0,598293871	3	6,54		1
764	NTGEQSGYHVE	Beta-2-adrenergic receptor	Insulin_receptor	S364	7,986	0,150943698	5	7,7175	0,110085194	4
765	RDTRDSEAQRL	Stomatin	PKA	S10	6,5275	0,551242914	4	6,75		1
766	GLRRSSKFCLK	Beta-2-adrenergic receptor	PKC	S262	10,02	0,154056267	6	9,01	0,367287353	6
767	TKRNSSPPPSP	ATP2B1	PKA	S1178	7,76	0,334065862	6	7,37	0,94669953	5
768	PSMRSSGTDRK	HOX B7	CK2alpha1	S133	8,6575	0,235199384	4	8,026666667	0,613994209	6
769	EDIKSYYTVRQ	PTPN1	Insulin_receptor	Y152	3,41	3,269872577	3	6,736666667	1,118222796	3
770	PDPSKYCGPYK	Aspartylglucosaminidase	UDP-n	Y178	6,055	0,245	2	6,426666667	0,148174072	3
771	RKGHEYTNIKY	SHP2	PDGFRbeta	Y542	9,865	0,312022969	6	10,402	0,422629862	5
772	IYSGDYYRQGR	c-Mer	c-Mer	Y753	0		1	3,09	3,09	2
773	NTTATYAEPYR	Plakophilin 4	nd	Y478	7,2		1	2,395	2,395	2
774	NegCtrl	Mixed peptides without STY	na							
775	SptCtrl	Used for production/QC	na		8,1225	0,359539636	4	7,643333333	0,526772141	6
776	PosCtrl	Mixed kinase substrate peptides	na		0	0	2	5,595	0,075	2
777	ETKSLYPSSEI	STAM2	EGFR	Y192	6,765	0,395	2	7,44	1,015209338	4
778	HVSISYDIPPT	GAB1	EGFR;HGFR	Y307	3,185	3,185	2	5,52		1

779	DSTNEYMDMKP	KIT	Choline_kinase	Y721	5,9375	0,46299973	4	2,83	2,83	2
780	KDGRGYVPATI	STAT6	IL4	Y641	6,03		1	6,64		1
781	EEGEMYEDDEE	Tubulin, beta-4	MAPkinase	Y437	5,746666667	1,033096747	3	3,89		1
782	MECRNSPVTKT	Eukaryotic translation initiation factor 4E binding protein 1	mTOR	S65	4,99	3,529315325	3	2,325	2,325	2
783	GQESEYGNITY	SHP1	c-Abl;Lck	Y536	7,49		1	7,515	0,325	2
784	DDQEVYDDVAE	FYB	Fyn	Y595	5,136666667	0,522961651	3	0		1
785	LSSSNTIRRPR	6-phosphofructo-2-kinase/fructose-2,6-biphosphatase 2	pkc	T475	7,9175	0,305971812	4	7,2675	1,03603511	4
786	AMFPETLDEGM	Beta-catenin	CK2beta	T102	6,31		1	6,453333333	1,102733976	3
787	SSEITTKDLKE	Prothymosin alpha	nb	T14	7,2025	0,211586271	4	7,99	0,374788829	3
788	MHRRHTDPVQL	GRID	nd	T262	5,856666667	0,426953029	3	6,84		1
789	SSRRTTLCGTL	Serine/threonine protein kinase 6	PKA	T288	6,21		1	6,86		1
790	HYLDETEQWEK	Complement component 3	CK	T1031	8,413333333	0,336683168	6	8,702	0,307662152	5
791	EGCACTPERMA	Survivin	CDC2	T34	6,335	0,615	2	7,02		1
792	AVQSGTPEEPE	Nucleolar protein 3	CK2	T149	4,82		1	4,875	0,265	2
793	NVDRVSIRSYR	Polymeric immunoglobulin receptor	nd	S637	7,8825	0,881174642	4	6,5175	0,994971733	4
794	GSSSQSQGISS	CHK2	PKA;PKG	S33	2,17	2,17	2	0,603333333	0,853242183	3
795	GQLVDSIAKTR	MAP2K4	MAP2K4	S257	6,075	0,015	2	5,69	0,58	2
796	CLTFGSPVLMR	Flap endonuclease-1	CDC2_Cyclin_A	S187	3,896666667	2,852721897	3	3,546666667	1,896599296	3
797	LARRPTKGIHE	Guanine nucleotide binding protein, alpha13	PKA	T203	6,6175	0,206926919	4	6,98	0,125698051	3
798	PFRRHSWICFD	cAMP specific 3',5 '-cyclic phosphodiesterase 4D	PKA	S13	6,285	0,581055075	4	5,43	1,52	2
799	AQSLATPVVSV	Myocyte specific enhancer factor 2C	MAPK14	T293	6,3675	0,283140866	4	1,8		1
800	RPESFTTPEGP	HBP	nd	T61	6,576666667	1,051802685	3	2,556666667	2,627373001	3
801	KGMIKSSRPLR	Formyl peptide receptor-like 1	nd	S236	9,19	0,161987654	5	8,433333333	0,434076286	6
802	RRRLSSLRAST	Ribosomal protein S6	PAK2	S236	8,871666667	0,394774901	6	8,091666667	0,616155193	6
803	GSKNKSPSKAA	High mobility group AT-hook 2	Cdc2_kinase	S59	7,758333333	0,309699675	6	8,15	0,197888858	5
804	PKEKDSPHMQD	Nuclear transcription factor Y, alpha	CDK2	S326	5,643333333	0,933856996	3	1,992	2,526803514	5
805	YVEKFSYKSIT	Leukotriene A4 hydrolase	nd	S416	6,3	0,06	2	7,34	0,164164552	4
806	EFISLSPPHEA	E2F transcription factor 1	CDK7	S403	6,678	0,430134863	5	7,408	0,650796435	5
807	PVRMSSTIFST	PAK2	PAK2	S20	3,313333333	2,343577512	3	2,67	2,710479662	4
808	MEEPQSDPSVE	p53	CK1alpha1	S6	7,6425	0,268456235	4	6,555	0,801420614	4
809	PGIDLSQVYEL	ABL	AKT	S465 466	9,766666667	0,309551647	6	9,871666667	0,230464507	6
810	LLFACSPPPAS	CDC 25A	CDC2	S17	1,014	2,028	5	2,5425	2,611765447	4
811	SLYASSPGGVY	Vimentin	CDC2	S56	8,051666667	0,339186117	6	8,096	0,382444767	5
812	TGVETSFRKLS	NFKB1	IKKalpha	S932	7,608	0,293625612	5	6,83	0,777785317	4
813	PGPSLSQGVSV	Nibrin	nb	S343	3,512	2,955499281	5	1	1,414213562	3
814	APDEGSDLFYD	Cell division cycle 34	CK2	S203	9,033333333	0,606483489	6	8,952	0,297751574	5
815	YSRQFSLEHVH	Heat-shock transcription factor 1	CaMKII	S230	8,978333333	0,21528405	6	8,721666667	0,458454529	6
816	PVQQPSAFGSM	Glycogen synthase kinase 3 beta	Serum/glucocorticoid_regulated_kinase_3	S21	5,906666667	0,910103779	3	2,495	2,495	2
817	PPPQLSPFLQP	Estrogen receptor, alpha	ERK2	S118	6,753333333	0,39835774	3	6,781666667	0,612927855	6
818	RQRSTSTPNVH	RAF1	PKA;AKT1	S259	8,883333333	0,342669261	6	8,845	0,202792998	6
819	GSHSNSFRLSN	Protein tyrosine phosphatase, receptor type, alpha	PKCdelta	S189	7,916666667	0,448317844	6	8,026666667	0,344512538	3
820	EDGEETCALAS	Ring finger protein 7	CK2beta	T10	4,876666667	2,369800742	6	6,5575	0,338996681	4
821	SQETFSDLWKL	p53	Campt;Chk1;CHK2;Plk3_;DNA-dependent_protein_kinase_catalytic_subunit_208900;ATR	S20	9,51	0,148772757	6	9,428	0,173827501	5
822	TTGGESCDELE	Occludin	CK2beta	S408	8,191666667	0,531394915	6	8,354	0,286607746	5
823	ISGRLSPIMTE	FKHR	DYRK1A_kinase	S329	6,884	0,439754477	5	3,18	3,196591935	4
824	VETQASQGTLQ	DNA-dependent protein kinase catalytic subunit	DNA-dependent_protein_kinase_catalytic_subunit	S2612	4,09	2,341478166	5	7,13	0,21	2
825	PPRRSSIRNAH	p47-phox	PKCzeta;PKCalpha;PKCbetaII;PKCdelta	S304	9,458333333	0,364207664	6	9,296666667	0,383652041	6
826	IDRTESLNRSI	Lymphocyte specific protein	MAPKAPkinaseII	S204	7,052	0,81641656	5	7,916	0,147594038	5
827	YCSRDSRGHND	Glutamate receptor ionotropic, N-methyl D-aspartate subunit 2A	PKC	S1416	7,0275	0,532511737	4	7,6	0,854254061	4
828	DNSSDSDYDLH	CD5	Lck;Fyn	T453	9,036666667	0,16038149	6	9,168	0,232155121	5
829	LLKLASPELER	c-Jun	JNK1	S73	7,526	0,360255465	5	7,736	0,373502343	5
830	EPKRRSARLSA	HMG14	Ribosomal_protein_S6_kinase_alpha_3;PKA;PKC	S7	9,24	0,18734994	6	8,93	0,360832371	5

831	RPDPSSFSRPR	Opioid receptor	PKC	S344	8,405	0,434424907	6	8,14	0,168720676	3
832	PIGEDEESESD	Vesicular monoamine transporter 2	CK1;CK2	S511	7,925	0,538199158	6	7,838	0,533119124	5
833	DINSLYDVSRM	Phospholipase C, gamma 2	Lyn;Lck;Fyn	Y753	1,615	2,797262054	4	6,09	1,424991228	5
834	PKDEVYSKYYT	STAT5B	c-Src	Y679	7,38	0,244867311	5	8,378333333	0,394943737	6
835	KDEGSYTLEEP	Syndecan 3	Tyrosine_specfic_kinase	Y361	5,2925	0,841200779	4	5,728333333	2,590253634	6
836	TPSAAYLWVGT	Gelsolin	Csk	Y603	7,466	0,422686645	5	7,183333333	0,474435337	3
837	FPDQAYANSQP	SIT	nd	Y188	2,15	2,15	2	3,49	3,49	2
838	NegCtrl	Mixed peptides without STY	na							
839	SptCtrl	Used for production/QC	na		9,2375	0,327060774	4	8,711666667	0,373783211	6
840	PosCtrl	Mixed kinase substrate peptides	na		4,063333333	2,181075168	3	3,61	2,556208129	3
841	DKQVEYLDLDL	GAB1	Insulin_receptor;EGFR;HGFR	Y627	7,814	0,181394597	5	8,391666667	0,531708462	6
**842**	**KAGNLYDISED**	**NR2B**	**Fyn**	**Y1252**	7,688	0,422629862	5	8,526666667	0,319095945	6
843	AEPHTYEEPGR	EphA8	EphA8	Y616	3,5	3,5	2	3,984	3,206696743	5
844	FTNPVYATLYM	Low density lipoprotein receptor-related protein 1	v-Src	Y4507	8,285	0,574536045	6	7,994	0,989638318	5
845	EEDHTYEGLDI	CD79B	Lyn;Fyn	Y196	8,494	0,504325292	5	9,305	0,40198466	6
846	TTEAIYEEIDA	TOM1 like 1	Fyn	Y460	8,306666667	0,648887938	6	8,64	0,379692858	6
847	DGSREYVNVSQ	LAT	ZAP70	Y220			0	6,186666667	0,020548047	3
848	KIGEGTYGVVY	CDC2	Tita: WEE/MYT PK161	T161	7,2	0,93284511	3	6,516666667	1,548554882	3
849	DEYNVTPSPPG	Notch2	GSK3beta	T2074			0	6,836666667	0,431225643	3
850	RKKRYTVVGNP	LIM domain kinase 1	ROCK1	T505	8,72	0,37013511	6	8,466	0,31941196	5
851	TDDEMTGYVAT	MAPK14	MAP2K3	T180	0	0	3	4,38	1,539242238	3
852	VLDDQYVSSVG	Bone marrow kinase BMX	c-Src	Y566	5,63	1,16	2	7,663333333	0,192930615	3
853	RRKAATMRERR	Myogenic differentiation antigen 1	PKC	T115	4,29	3,215182732	3	5,373333333	1,136378263	3
854	QSRRSTQGVTL	Myosin phosphatase target subunit 1	Rho_kinase;DMPK;ILK	T696	4,823333333	3,451032052	3	5,64		1
855	CTHDLYMIMRE	FGF receptor 3	FGFR3	Y724	0		1	6,34		1
856	EQRNVYKDYRQ	Dynamin 1	c-Src;v-Src	Y597	5,713333333	2,169444373	3			0
857	EKPRLSFADRA	Protein kinase C, theta	nd	S676	5,26		1	3,44		1
858	NNTSSSPQPKK	p53	CDK2	S315	5,415	0,485	2	7,285	0,599854149	4
859	LAHNVSKDNRQ	Phosphatidylinositol 3 kinase, catalytic subunit, delta	Phosphatidylinositol_3_kinase,_catalytic_subunit,_delta	1039 / S10	4,76	2,795487435	4			0
860	LAVVGSPYWMA	Testis specific protein kinase 1	Testis_specific_protein_kinase_1	S220	8,326	0,788837119	5	8,775	0,215	2
861	VAARATLRRSN	Calcium sensing receptor	PKC	T888	8,633333333	0,591852084	6	8,27	0,651613382	5
862	YQMALTPVVVT	Cyclin-dependent kinase 4	Discoidin_domain_receptor	T172	5,85	0,369143152	3	7,43	0,27	2
863	RPRGYTISDSA	Tuberin	AKT1	T1462	6,24		1			0
864	PKGTGYIKTEL	STAT1	JAK1;JAK2;TYK2;EGFR;Lck	Y701	11,14		1			0
865	LRSEFSPSVDA	c-Mos	nd	S16	5,97		1	7,14	0,200166597	3
866	QLGPPSPVKMP	Pituitary tumor-transforming protein 1	CDC2	S165	3,39	3,39	2	6,71		1
867	PGLRRSPIKKV	B-Myb	CDK2	S577	8,916666667	0,09049248	6	8,542	0,164972725	5
868	QGISFSQPTCP	Cell cycle checkpoint kinase	ATR	S345	3,045	3,284177979	4	4,57		1
869	TRDHPSTANTV	Opioid receptor mu 1	nd	S377	0	0	3	0,863333333	1,220937709	3
870	DRSDISSTCAN	TGF beta receptor, type II	nd	S228	4,68		1	1,21		1
871	RSGAMSPMSWN	FADD	FIST3_70-kDa_kinase	S194	3,78	3,78	2	5,79	1,08	2
872	FSEHRTQVSLK	Cell cycle checkpoint kinase II	CHK2	T432	0	0	2			0
873	SEGSSSGRARE	Androgen receptor	AKT1	S208	2,72	2,72	2	0	0	2
874	GFFSSSESGAP	Clathrin light polypeptide B	CK2	S11	6,842	0,708728439	5	7,718333333	0,363291282	6
875	STQTPSPPCQA	BCL2-interacting protein BIM	JNK1	S118	2,484	1,463866114	5	0,286	0,572	5
876	DGRGDSVVYGL	Secreted phosphoprotein 1	Phosphotyrosyl_protein_phosphatase	S148	8,733333333	0,319409177	6	9,066666667	0,286627904	6
877	RPRRRSSCVSL	Cyclic GMP inhibited phosphodiesterase B	AKT1	S295	8,455	0,258182752	6	7,996	0,496088702	5
878	AGDLESPLSEE	PPAR	MAPK	S21	8,565	0,409867865	6	8,185	0,571977564	6
879	RSRSGSIVELI	Huntingtin	AKT	S421	7,235	0,447800179	4	7,4925	0,635388661	4
880	RAREGSFESRY	Phospholipase C, gamma1	PKCmu	S1248	8,915	0,342137886	6	8,47	0,287460142	6
881	GEKRASSPFRR	Nucleolar phosphoprotein p130	PKA	S623	9,091666667	0,455170908	6	8,488	0,193948447	5
882	SKRRNSEFEIF	Tryptophan hydroxylase	PRKA1	S58	8,845	0,224555116	6	8,631666667	0,20432137	6

883	ESRSGSNRRER	Wiskott-Aldrich syndrome protein interacting protein	PKCtheta	S488	8,365	0,284648907	6	7,722	0,283365488	5
884	HSLPLSPASTR	NFKB1	GSK3beta	S907	7,816	0,361696005	5	7,221666667	1,017339941	6
885	RDRSSSAPNVH	B-Raf	AKT;SGK	S364	6,475	0,564350659	6	4,895	2,886472761	4
886	RLLGHSPVLRN	CDC 25B	CDC2	S146	6,41	1,113126228	4	6,996	0,894887702	5
887	NQSLLSPLVLE	Keratin 8	p38_kinase;JNK;p42_kinase	S74	8,605	0,483313218	6	8,44	0,422610932	5
888	ERMNCSPTSQI	AKT3	Pyruvate_dehydrogenase_kinase,_isoenzyme_1	S120	3,975	2,318129634	4	3,77	2,669619199	3
889	KSKKYSDVEVP	Adducin 1	PKA	S408	7,968333333	0,900803654	6	7,945	0,513638978	6
890	VVRRLSTQFTA	Perilipin	PKA	S81	4,913333333	3,984782163	3	7,46	2,074162482	4
891	SPPRSSLRRSS	Transcription elongation factor A- like1	PKC;GSK3	S37	9,368	0,34970845	5	8,911666667	0,330928828	6
892	SGDDVSEQDVP	TFIIA-alpha/beta like factor	CK2	S423	6,1625	0,361204582	4	7,153333333	0,615431195	3
893	QHRSSSSAPHH	Calcium channel voltage dependent beta 2 subunit	DNA-dependent_protein_kinase_catalytic_subunit	S478	8,626666667	0,48230926	6	8,011666667	0,837285628	6
894	KKPKKKSCLLL	Ras related protein 1A	PKA	S180	8,855	0,546038765	6	8,494	0,376435918	5
895	GGRGGSRARNL	Heterogeneous nuclear ribonucleoprotein K	PKCdelta	S302	8,766666667	0,308040401	6	7,994	0,409125897	5
896	PTAPLSPMSPP	Connexin 43	ERK1	S279	0		1	4,5075	2,604048531	4
897	QVKALYDFLPR	Protein tyrosine kinase TXK	Protein_tyrosine_kinase_TXK	Y91	7,362	0,481763427	5	7,266666667	0,27329268	3
898	ADENYYKAQTH	SYK	SYK	Y526	5,844	1,148992602	5	5,31	0,303424895	3
899	GTDLEYLKKVR	O-linked N-acetylglucosamine transferase	Tyrosine_kinase	T989	5,26		1	3,993333333	2,880975915	3
900	SSGEWYQNFQP	CD6	nd	Y629	4,97		1			0
901	DADENYFINEE	Phosphatidylinositol 3-kinase, regulatory gamma subunit	nd	Y341	5,838	0,88454282	5	6,775	0,775	2
902	NegCtrl	Mixed peptides without STY	na							
903	SptCtrl	Used for production/QC	na		8,158333333	0,228795153	6	7,742	0,478430768	5
904	PosCtrl	Mixed kinase substrate peptides	na		1,95	1,95	2	2,86		1
905	DIDGQYAMTRA	Beta-catenin	pp60-src	Y86	5,5		1	1,585	1,585	2
906	IPSVPYAPFAA	Guanine nucleotide releasing factor 2	Hck	Y522	6,175	1,585	2	5,98		1
907	RQGKDYVGAIP	VEGF receptor 2	VEGF_receptor_2	Y951	5,61		1			0
908	PYRSPPPYVPP	Dystrophin associated glycoprotein 1	c-Src	Y892	0		1			0
909	LTTGVYVKMPP	CD152	JAK2,_Protein_tyrosine_kinase_TXK	Y201	5,975	0,905	2	2,645	2,645	2
910	SSDDDYDDVDI	HPK1	SYK	Y381	6,355	1,025	2	6,56	0,414487636	3
911	NSKRDYTGCST	PZR	SHP2	Y200	0,99	0,99	2	0		1
912	DKSSAHSY	Peripherin	nd	Y470	0		1	2,78	2,78	2
913	NYIPETPPPGY	SMAD2	ERK	T220	7,165	0,4140954	4	7,63	0,424578222	6
914	APAAPTPAAPA	p53	JNK1	T81	0	0	2	2,2	2,2	2
915	PNSSKTYGIKW	Calpain, large polypeptide L1	nd	S360	6,8425	0,249937492	4	7,3425	0,336999629	4
916	IGDELYLEPLE	RAD9	ABL	Y28	7,2425	0,665558976	4	7,11	0,15	2
917	IEQWFTEDPGP	p53	ERK2	T55	7,25	0,64	2	7,08	0,479040708	5
918	PSEVPTPKRPR	High mobility group AT-hook 1	CDC2_kinase	T52	5,616666667	1,230401922	3	4,626666667	1,806752765	3
919	PSGLLTPPQSG	Cyclin E1	nd	T395			0	5,18	1,42	2
920	GKRHRYRVLSS	Antisense ERCC1	PKA	Y80	6,916666667	0,926726617	3	7,2175	0,74045172	4
921	SYSHHSGLEYA	Caveolin 2	c-Src	Y19	7,236	0,554818889	5	7,458	0,203607465	5
922	APAYLSSPLAL	IKK Gamma	IKKbeta	S376	6,545	1,064459957	4	6,36	0,818901704	4
923	AWTADSGEGDF	Fibrinogen, alpha chain	nd	S22	8,384	0,230616565	5	8,16	0,379209705	5
924	ESLDQSMEEEE	CTD phosphatase, subunit 1	CK2	S575	6,572	0,342426634	5	4,87		1
925	SVSVETQGDDW	Hematopoietic cell specific LYN substrate 1	CK2alpha_1	T16	7,87	0,266983146	5	7,69	0,556309267	5
926	CRIGGSRRERS	EP4 receptor	PKC	S354	5,595	0,325	2	7,48		1
927	ALVRGTPVRGA	Sam68	CDC2	T317	4,86	1,8	2	0		1
928	MRGILYAAPQL	CD19	Lyn_kinase	Y409	7,975	0,335	2	4,076666667	2,984117215	3
929	CSSLSSLSSAE	APC	CK1epsilon	S1279	6,36		1	6,76		1
930	SQKYMSFTDKS	PAK1	PAK1	S144	7,9075	0,110312057	4	7,881666667	0,394648226	6
931	PTRKISASEFD	Phosphodiesterase 5A	cGMP_dependent_protein_kinase	S102	2,563333333	0,48230926	3	4,1175	2,398003075	4
932	IIRQPSEEEII	PEA15	Akt;CaMKII	S116	7,262	0,36035538	5	7,62	0,351454122	5
933	RCRLLSDCANV	Crystallin beta A2	nd	S31	2,158	1,909569585	5	4,83		1
934	ALSTDSIERLP	Pyruvate dehydrogenase kinase, isoenzyme 1	Pyruvate_dehydrogenase_kinase,_isoenzyme_1	S393	5,536666667	0,726100697	3	7,345	0,185	2

935	GALSNSESIPT	AMSH	Bone_morphogenetic_protein_receptor,_type_IB	S245	5,033333333	0,250377493	3	0	0	3
936	IFRRPSLPCIS	Cyclic GMP inhibited phosphodiesterase B	PKA	S318	5,84	1,71866227	4	4,95		1
937	LQEVLSSDENG	CHOP	MAPK14	S79	7,26	0,350257048	5	7,855	0,729743105	4
938	KTVNESASLRE	Insulin receptor	Protein_kinase_C_alpha	S1064	2,1125	2,114986702	4	1,576666667	2,229743383	3
939	VSRSTSFRGGM	Keratin 18	CAMK;Ribosomal_protein_S6_kinase_alpha3;PKCepsilon	S52	7,4125	0,087856417	4	6,926	0,443738662	5
940	IVPGKSPTRKK	RAP1 GTPase activating protein 1	CDC2	S484	8,33	0,49149432	6	7,7	0,470956474	6
941	HSIYSSDEDDE	c-Myb	ERK2	S532	7,593333333	0,348982648	6	7,5025	0,227637321	4
942	KSEPISPPRDR	Myocyte specific enhancer factor 2A	CDK5	S408	5,812	1,758083047	5	5,1975	1,001333486	4
943	APERASSVYTR	CCR5	GRK	S336	8,616666667	0,305923011	6	7,845	0,582773541	4
944	RPPTLSPIPHI	Retinoblastoma 1	Cyclin_D1;Cdk4	S780	7,391666667	0,326211417	6	7,4275	0,630133914	4
945	RKGYRSQRGHS	Vitronectin	PKC	S381	9,193333333	0,251639071	6	8,473333333	0,425936876	6
946	EKRKNSILNPI	CFTR	PKC	S686	7,405	0,205608852	4	6,91	0,43	2
947	RDRHLSFSGSG	CD44	PKC	S291	7,723333333	0,284468315	6	6,58	1,45	2
948	SMPAFSPGPGI	Sterol regulatory element binding transcription factor1	Sterol_regulatory_element_binding_transcription_factor1	S117	4,49	1,710511619	4	4,36	3,086886241	3
949	RERKSSAPSHS	GAB2	AKT1	S159	6,41	0,461605893	5	5,16	0,06	2
950	LEPQKSLGDEG	Phospholipase C, beta 3	CaMKII	S537	6,8	0,324037035	3	7,075	0,025	2
951	VSEDNSEDEIS	Acetyl-CoA carboxylase alpha	CK2	S29;S66	7,861666667	0,368303166	6	8,02	0,18547237	3
952	DSMANSFVGTR	MEK1	p74RAF-1	S222	6,616666667	0,084983659	3	7,68		1
953	DIQQLSSEEND	Asialoglycoprotein receptor 2	PKC	S12	8,0125	0,706235619	4	7,89	0,335797558	5
954	MQRKLSVALAF	ATP-binding cassette, subfamily A, member 1	PKA	S1042	4,855	1,235	2	3,8525	2,304467129	4
955	AKKTLSEVERD	Sperm associated antigen 1	PKC	S317	8,11		1	6,61	0,22	2
956	KERWGSNELPA	Sarcoplasmic/endoplasmic reticulum calcium ATPase 2	CAMKII	S38	5,67		1	5,5		1
957	HLRSESQRQRR	Guanine nucleotide binding protein, alpha Z polypeptide	PKC	S27	8,468333333	0,195056972	6	8,01	0,349342239	5
958	DDGRVSYPLCF	Glia maturation factor beta	PKC	S72	8,68	0,433282048	6	7,881666667	0,504823952	6
959	ASVPPSPSLSR	Glycogen synthase 1	GSK3beta	S645	8,59	0,328481354	6	7,852	0,160299719	5
960	KNKPRSPVVEL	Beta-adrenergic receptor kinase1	MAPkinase	S670	7,16	0,765462823	6	5,978	3,008444116	5
961	AENPEYLGLDV	ErbB2	ERBB2;EGFR	Y1248	6,473333333	0,10402991	3	6,72		1
962	FIGEHYVHVNA	Hepatocyte growth factor receptor	HGFR	Y1349	5,6	0,93	2	6,67		1
963	SETDDYAEIID	FAK	FAK	Y397	7,273333333	0,7825741	3	6,966666667	0,650350333	3
964	VDSGVYAVPPP	p130CAS	nd	Y410	5,305	0,055	2	3,553333333	2,54172295	3
965	SSTHYYLLPER	ACK	nd	Y858	6,941666667	0,367033907	6	6,9475	0,549198279	4
966	NegCtrl	Mixed peptides without STY	na							
967	SptCtrl	Used for production/QC	na		6,62	0,743740546	4	6,23	0,553714728	3
968	PosCtrl	Mixed kinase substrate peptides	na		5		1	0,14		1
969	EANSHYGHNDD	CD31	Lck;Csk	Y663	6,24		1	6,35	0,227596134	3
970	IEDNEYTAREG	Lck	nd	Y411	5,885	0,555	2	5,6		1
971	SEMTGYVVTRW	MAPK12	MAP2K3	Y185	8,023333333	0,434843522	6	7,313333333	0,541130504	3
972	SSGADYPDELQ	Trypsin 1	nd	Y154	6,806666667	1,179896417	3	6,05		1
973	SAEPQYQPGDQ	Fgr	Csk	Y523	5,59	0,98	2	6,29	0,19	2
974	AFPVSYSSSGA	Ret	Ret	Y687	2,455	2,021045522	4	4,856666667	1,468703132	3
975	ENFDDYMKEVG	Fatty acid binding protein 4	Tyrosyl_kinase	Y20	2,756666667	2,828431053	3	2,625	2,625	2
976	YYEGYYAAGPG	LPP	nd	Y301	6,77		1	2,395	2,395	2
977	YLTQETNKVET	Parathyroid hormone related protein	CDK2	T108	3,86	2,97	2	7,09		1
978	DVPLLTPSSKE	C ets 1 protein	HGF	T38	7,054	0,463836178	5	7,038	0,682067445	5
979	ASKMDTCSSNL	Protease activated receptor 1	nd	T410	2,94	2,94	2	5,825	0,045	2
980	MFPRNYVTPVN	Grb2	EGFR;Bcr/Abl	Y209	4,22	1,699078181	3	6,41		1
981	RKRRPTSGLHP	BRCA1	AKT1	S509	7,423333333	0,10143416	3	6,77	1,26	2
982	LRGSATRALPP	Casein kinase 1, epsilon	CK1epsilon	S368	4,54		1	0	0	2
983	VYPTKTFPNLY	Ectonucleotide pyrophosphatase /phosphodiesterase 2	nd	T210	6,48		1	6,21	0,876622306	3
984	TDRSPYEKVSA	Mucin 1 transmembrane	EGFR	Y1229	5,62	0,3	2	5,98		1
985	DHYRYSDTTDS	PTEN	AKT1	S380	6,3725	0,58780843	4	6,61	0,387384391	3
986	LDSAQSPGPSW	CDX2	MAPkinase	S60	6,993333333	0,580880558	3	6,94	0,606259021	4

987	KEEKGSPLNAA	PTPN1	CK2	S352	6,51		1	4,5125	1,748233037	4
988	HTGFLTEYVAT	ERK2	ERK2	T185	7,67	0,221359436	4	7,293333333	0,269917683	6
989	GTLFSTTPGGT	Eukaryotic translation initiation factor 4E binding protein 1	MAPkinase	T45	3,1	2,196421332	3	1,753333333	1,475586512	3
990	PDVPRTPVGKF	CDC25C	Polo_like_kinase	S198	7,69	0,89506983	4	7	0,560133913	4
991	SQPGHTPHTAA	Bcl 2	nd	T56	5,396666667	0,685338521	3	5,1	1,716726536	4
992	EDEESYDTESE	I-Kappa-B-alpha	CK2	S288	7,0475	0,212294018	4	6,89	0,775671752	3
993	QFITNSEEVRL	MAD2 mitotic arrest deficient-like 1	nd	S178	6,43	0,512132795	5	6,816	0,429259828	5
994	GQVIMSIRTKL	Ribosomal protein L10	Ribosomal_protein_L10	S137	8,201666667	0,470014775	6	7,5525	0,322519379	4
995	KFDTNSHNDDA	Growth hormone 1	nd	S176	5,125	1,007906246	4	6,475	0,145	2
996	TTAAHSLVGTP	Serine/threonine protein kinase Nek6	Nek9	S206	5,02		1	2,013333333	2,847283306	3
997	LCQAFSDVILA	Cyclin B1	Polo_like_kinase	S147	8,081666667	0,223339552	6	7,24	0,707837081	6
998	SFGEPSYPEVF	Presenilin 2	CK2	S335	9,325	0,400697309	6	8,722	0,137753403	5
999	MARGSVSDEEM	Lymphocyte cytosolic protein 1	PKA	S5	6,504	0,997047642	5	6,508	0,970492658	5
1000	LSSSESPQRDP	Cell division autoantigen 1	CDK2	S20	0		1	0		1
1001	LRQLRSPRRAQ	Ras associated protein Rab4	CDC2	S204	8,848	0,215907388	5	8,362	0,288956744	5
1002	PLNSVSPSPLM	Estrogen receptor, alpha	MAT1	S104	1,7575	1,757588902	4	5,375	1,195	2
1003	KQFLISPPASP	Down syndrome critical region protein 1	MAPkinase	S112	6,246666667	0,771549667	3	6,76	0,584593876	4
1004	SVVSERISSV	5-hydroxytryptamine receptor 2C	nd	S456	6,762	0,644093161	5	7,11	0,276134025	4
1005	GSDSDSEVDKK	PC4	CK2alpha_1	S19	5,356	0,57822487	5	5,912	0,70169509	5
1006	NASASSLKKKQ	Fascin 1	PKC	S38	7,986666667	0,619399351	6	7,778	0,70556077	5
1007	RKRHNSISEAK	Phospholipase C, beta 3	PKG	S1105	8,665	0,244523346	6	8,481666667	0,43521706	6
1008	ENSPKSPKVTG	Centromeric protein E	nd	S2570	2,19	2,19	2	5,16	0,858108385	4
1009	TLPRNSGAGAS	Glutamate receptor ionotropic, AMPA 1	CAMKII	S863	0	0	4	0,68	1,177794549	4
1010	RSYVSSGEMMV	Glial fibrillary acidic protein	PKC;RHO_kinase;CAMKII	S17	6,77	0,18069311	4	7,09	0,23	2
1011	QVSSLSESEES	Activating transcription factor 1	CK2	S36	8,278333333	0,327994749	6	7,91	0,501238466	5
1012	TDGNRSSHSRL	BH3 interacting domain death agonist	CK1;CK2	S65	4,4	2,238008043	5	3,093333333	2,96963896	3
1013	SRVTFSEDDEI	NIPP1	CK2	S204	7,946	0,356796861	5	7,488	0,424895281	5
1014	SSQRVSSYRRT	Desmin	Aurora_kinase_B	S12	8,9	0,208326667	6	8,293333333	0,688613744	6
1015	RAHGLSLIPST	Microphthalmia associated transcription factor	GSK3beta	S399	2,285	2,447452757	4	2	2,007045922	6
1016	LSGRGSNYGSL	NPRA	nd	S538	8,592	0,391070326	5	8,035	0,870550592	6
1017	RFYPESSYKST	Occludin	PKC	S340	9,583333333	0,259208196	6	9,136666667	0,415237549	6
1018	LPRASSLNENV	PPP1R9B	PKA	S100	6,785	0,663079935	4	6,6475	0,895638739	4
1019	YETFKSIMKKS	NFKB3	PKCzeta	S298	6,595	0,845	2	4,1125	2,702622569	4
1020	ISPPASPPVGW	Down syndrome critical region protein 1	GSK3	S108	7,693333333	0,081785628	3	7,39	0,237592087	4
1021	REKKFSTKSDV	Csk	PKA	S364	8,118333333	0,501411895	6	7,866	0,27861084	5
1022	PSRSYSERDFE	Solute carrier family anion exchange, member 3	PKCepsilon	S67	8,486666667	0,391180549	6	7,992	0,465549138	5
1023	PVAPLSPARLQ	ELK3	MAPK14;EphB2	S363	7,934	0,463922407	5	7,045	1,249916664	6
1024	ARVLGSEGEEE	LIG1	CK2alpha1	S66	7,56	0,242487113	5	7,34	0,331360831	3
